# Supplementary material for: Collective total synthesis of stereoisomeric yohimbine alkaloids
Source: Nat Commun. 2024 Jan 31;15:941. doi: 10.1038/s41467-024-45140-2 (PMC10830567; doi:10.1038/s41467-024-45140-2)
Supplement: Supplementary file 1 — Supplementary Information [file 41467_2024_45140_MOESM1_ESM.pdf]

Supplementary Information for

Collective total synthesis of stereoisomeric yohimbine alkaloids

Meiyi Tang, Haigen Lu, Liansuo Zu*

Corresponding author. Email: zuliansuo@tsinghua.edu.cn

Supplementary Methods

Unless stated otherwise, reactions were conducted in dry glassware using anhydrous solvents (passed through activated alumina columns). All commercially available reagents were used as received unless otherwise specified. Reaction temperatures were controlled using an IKA RCT basic temperature modulator.

Thin layer chromatography (TLC) was conducted on plates (GF254) supplied by Yantai Chemicals (China) and visualized using a combination of UV, anisaldehyde, iodine, and potassium permanganate staining. Silica gel (200-300 mesh) supplied by Tsingtao Haiyang Chemicals (China) was used for flash column chromatography.

All NMR spectra were recorded on Bruker AVANCE III HD 400MHz. Spectra were referenced internally to the residual proton resonance in $CDCl_3$ or DMSO-d6 (δ ^{1}H = 7.26 and 2.46 ppm, δ ^{13}C = 77.0 and 40.0 ppm) with tetramethylsilane (TMS, δ 0.00 ppm) as the internal standard. Data for ^{1}H NMR spectra were reported as follows: chemical shift (δ ppm), multiplicity, coupling constant (Hz) and integration. ^{13}C NMR spectra are reported in terms of chemical shift.

High resolution mass spectrometry (HRMS) data were obtained on a Xevo G2-XS QTof Quadrupole Time-of-Flight Mass Spectrometry by electrospray ionization (ESI) in the positive ion mode from Waters Corporation.

The enantiomeric excesses (ee) of products were determined on a Shimadzu LC-20AT Chiral HPLC. Optical rotations were recorded on a SG WZZ-2B automatic polarimeter.

Supplementary Notes

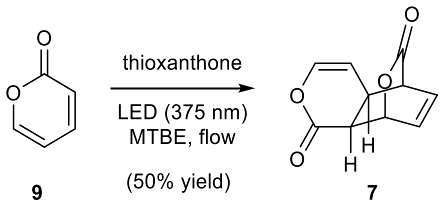

The known compound **7** was prepared in a single step following a previously established protocol[1]: A solution of pyrone **9** (6 g, 62.5 mmol) and thioxanthone (266 mg, 1.25 mmol; 2 mol%) in MTBE (2500 mL; 0.025 M) was degassed by sparging with argon for 15minutes. The resulting solution was pumped to flow through an array of 375 nm LEDs at rt. The eluents were collected and concentrated under reduced pressure. The resulting orange residue was purified by flash column chromatography (petroleum ether/ethyl acetate/dichloromethane=1:1:0.5) to afford dimeric products **7** as the major product (5.94 g, 50%).

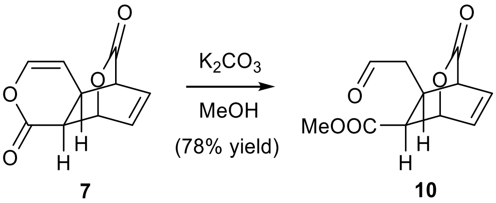

To a stirred solution of **7** (5.4 g, 28.1 mmol) in dry methanol (280 mL; 0.1 M) at 0 °C was added K_2CO_3 (388 mg, 2.81 mmol) under N_2. After being stirred for 10 min, the reaction was quenched with 1N HCl until pH = 6 and concentrated under reduced pressure. The residue was diluted with water (500 mL) and extracted with ethyl acetate (3 x 500 mL). The combined organic phase was washed with water (500 mL) and brine (500 mL), dried over Na_2SO_4, and concentrated under reduced pressure to give the crude residue. The crude product was purified by silica gel column chromatography (DCM / MeOH = 20:1) to afford the product **10** (4.9 g, 78 % yield) as a pale-yellow oil.

^{1}H NMR (400 MHz, $CDCl_3$) δ 9.74 (s, 1H), 6.61 (ddd, J = 8.0, 6.1, 2.0 Hz, 1H), 6.55 (ddd, J = 7.6, 5.0, 1.5 Hz, 1H), 5.40 (d, J = 5.1 Hz, 1H), 3.71 (s, 3H), 3.39 (dt, J = 6.3, 1.9 Hz, 1H), 3.14 (dd, J = 18.5, 8.1 Hz, 1H), 3.00 (dd, J = 10.4, 1.2 Hz, 1H), 2.78 – 2.55 (m, 2H).
^{13}C NMR (100 MHz, $CDCl_3$) δ 199.80, 171.69, 170.87, 133.32, 131.90, 75.07, 52.35, 46.19, 45.05, 44.31, 28.94.
HRMS-ESI (*m/z*): $[M - H]^-$ calculated for $C_{11}H_{12}O_5$, 223.0606; found 223.0641.

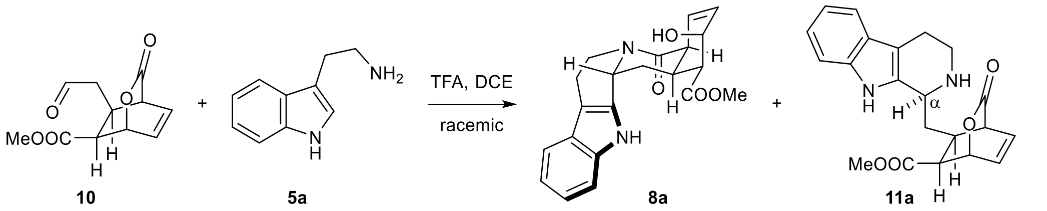

To a stirred solution of **10** (2.05 g, 9.14 mmol) and **5a** (1.46 g, 9.14 mmol) in DCE (91 mL; 0.1 M) at room temperature under N_2 atmosphere was slowly added TFA (0.82 ml, 11 mmol; 1.2 eq.). The resulting solution was stirred at room temperature for 12 hours. The reaction was concentrated under reduced pressure to afford the crude product, which was purified by silica gel column chromatography (DCM / MeOH = 20:1) to afford **8a** (1.67 g, 50%) as a pale-yellow powder and **11a** (650 mg, 20%) as a yellow oil.

8a:^{1}H NMR (400 MHz, DMSO-d6) δ 10.86 (s, 1H), 7.37 (dd, J = 7.9, 5.6 Hz, 2H), 7.06 (t, J = 7.7 Hz, 1H), 6.97 (t, J = 7.4 Hz, 1H), 5.95 – 5.78 (m, 1H), 5.70 (dd, J = 10.0, 2.2 Hz, 1H), 5.22 (d, J = 5.1 Hz, 1H), 4.85 (s, 1H), 4.77 (dd, J = 12.3, 5.1 Hz, 1H), 4.45 – 4.36 (m, 1H), 3.65 (s, 3H), 3.18 (d, J = 5.2 Hz, 1H), 3.04 (d, J = 2.7 Hz, 1H), 2.98 – 2.83 (m, 1H), 2.75 (m, 2H), 2.68 – 2.54 (m, 2H), 2.34 – 2.17 (m, 1H).
^{13}C NMR (100 MHz, DMSO-d6) δ 172.54, 170.05, 136.38, 135.00, 130.25, 127.19, 126.80, 121.26, 118.98, 117.96, 111.83, 108.67, 62.91, 54.14, 51.76, 46.88, 45.02, 42.39, 28.00, 25.30, 21.30.
HRMS-ESI (*m/z*): $[M + H]^+$ calculated for $C_{21}H_{23}N_2O_4$, 367.1658; found 367.165.

11a: ^{1}H NMR (400 MHz, $CDCl_3$ δ 10.03 (s, 1H), 9.47 (s, 1H), 9.24 (s, 1H), 7.45 (d, *J* = 7.8 Hz, 1H), 7.39 (d, *J* = 8.1 Hz, 1H), 7.26 – 7.18 (m, 1H), 7.18 – 7.10 (m, 1H), 6.41 (s, 1H), 6.29 (d, *J* = 5.7 Hz, 1H), 5.35 (d, *J* = 4.5 Hz, 1H), 4.73 (s, 1H), 3.75 (s, 3H), 3.48 (d, *J* = 6.5 Hz, 2H), 3.35 (s, 1H), 2.97 (dd, *J* = 16.1, 8.2 Hz, 3H), 2.67 (t, *J* = 12.8 Hz, 2H), 2.12 (d, *J* = 11.2 Hz, 1H).
^{13}C NMR (100 MHz, $CDCl_3$) δ 173.12, 171.81, 136.58, 133.65, 131.07, 128.23, 125.90, 123.02, 120.03, 118.25, 111.73, 106.86, 75.97, 52.68, 51.94, 46.89, 44.28, 40.31, 34.75, 31.54, 18.38.
HRMS-ESI (*m/z*): $[M + H]^+$ calculated for $C_{21}H_{23}N_2O_4$, 367.1658; found 367.1650.

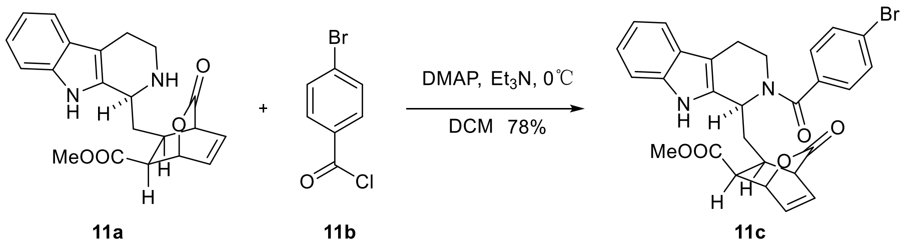

Et_3N (71.2 μl, 0.54 mmol) and **11b** (39 mg, 0.18 mmol) were added to a solution of **11a** (65 mg, 0.18 mmol) and DMAP (2.2 mg, 0.018 mmol) in anhydrous DCM (3.6 mL) at

0 °C under N_2. The resulting solution was stirred at 0 °C for 15 min. The reaction was concentrated under reduced pressure to afford the crude product, which was purified by silica gel column chromatography (DCM / MeOH = 20:1) to afford **11c** (76 mg, 78%) as a white powder.

^{1}H NMR (400 MHz, CDCl3) δ 8.54 (s, 1H), 7.61 (d, J = 8.2 Hz, 2H), 7.45 (d, J = 7.8 Hz, 1H), 7.41 (d, J = 8.1 Hz, 1H), 7.32 (d, J = 8.2 Hz, 2H), 7.21 (t, J = 7.6 Hz, 1H), 7.12 (t, J = 7.5 Hz, 1H), 6.69 (t, J = 6.5 Hz, 1H), 6.57 (t, J = 6.5 Hz, 1H), 5.65 (d, J = 10.4 Hz, 1H), 5.47 (d, J = 5.0 Hz, 1H), 5.37 (t, J = 4.8 Hz, 1H), 3.90 (s, 3H), 3.54 – 3.44 (m, 1H), 3.38 (d, J = 9.8 Hz, 1H), 3.32 (d, J = 6.2 Hz, 1H), 2.92 – 2.70 (m, 3H), 2.44 (d, J = 10.5 Hz, 1H), 2.23 (dd, J = 15.9, 8.3 Hz, 1H).

^{13}C NMR (100 MHz, CDCl3) δ 172.83, 171.79, 171.02, 136.06, 135.06, 134.31, 132.92, 131.90, 131.12, 128.13, 126.25, 124.15, 122.18, 119.57, 117.91, 111.27, 107.13, 52.81, 49.18, 47.93, 44.02, 42.02, 36.89, 34.17, 22.29, 14.11

HRMS-ESI (m/z): $[M + H]^+$ calculated for C28H26N2O5Br, 549.1025; found 549.1006.

Supplementary Table 1. Optimized reaction conditions for enantioselective synthesis of **8**

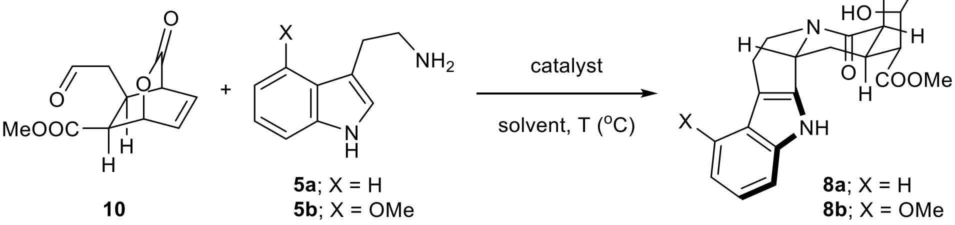

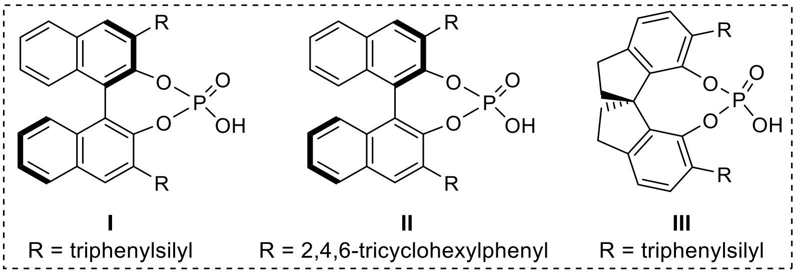

entry	catalyst	loading	solvent	T (°C)	5:10	yield (%)	ee
1	I	20 mol%	DCE	rt	1:1	**5a**: 45	62
2	II	20 mol%	DCE	rt	1:1	**5a**: 27	77
3	III	20 mol%	DCE	rt	1:1	**5a**: 30	-73
4	II	20 mol%	THF	rt	1:1	**5a**: 25	92
5	II	20 mol%	toluene	rt	1:1	**5a**: 30	86
6	II	20 mol%	dioxane	rt	1:1	**5a**: 45	95
7	II	20 mol%	THF	rt	1:2	**5a**: 78	94
9	II	20 mol%	dioxane	10	1:2	**5a**: 72	97

10	II	10 mol%	dioxane	10	1:2	**5a**: 70	95
11	II	10 mol%	dioxane	10	1:2	**5b**: 80	91

To a stirred solution of **10** (397 mg, 1.78 mmol; 2.0 eq.) and **5a** (148mg, 0.89 mmol) in dioxane (18 mL; 0.05 M) at 10 °C under N_2 atmosphere was added catalyst (**II**) (0.089 mmol, 10 mol%). The resulting solution was stirred at 10 °C for 24 hours. After evaporation of the solvent under reduced pressure, the residue was purified by silica gel column chromatography (DCM / MeOH = 100:1 to 20:1) to afford **8a** (230 mg, 70%) as a pale-yellow powder. $[\alpha]^{20}_{D}$ = 34.0 (*c* = 0.01, $CHCl_3$: MeOH 2:1).

8b: ^{1}H NMR (400 MHz, $CDCl_3$) δ 8.16 (s, 1H), 7.07 (t, J = 7.9 Hz, 1H), 7.00 (d, J = 8.0 Hz, 1H), 6.48 (d, J = 7.6 Hz, 1H), 5.98 (s, 2H), 4.92 (dd, J = 12.2, 6.0 Hz, 1H), 4.84 (s, 1H), 4.61 (s, 1H), 3.88 (s, 3H), 3.80 (s, 3H), 3.27 – 3.14 (m, 1H), 3.14 – 3.08 (m, 1H), 3.06 – 2.94 (m, 2H), 2.70 – 2.63 (m, 1H), 2.57 (d, J = 5.1 Hz, 1H), 2.53 (dd, J = 10.9, 3.7 Hz, 2H), 2.44 – 2.36 (m, 1H), 1.66 (s, 1H).
^{13}C NMR (100 MHz, $CDCl_3$) δ 172.94, 169.91, 154.39, 137.47, 131.49, 128.86, 127.39, 122.79, 117.80, 110.57, 104.65, 99.98, 62.87, 55.28, 54.28, 52.09, 46.54, 45.96, 43.41, 27.55, 24.66, 23.10.
HRMS-ESI (*m/z*): $[M + H]^+$ calculated for $C_{22}H_{25}N_2O_5$, 397.1763; found 397.1780. $[\alpha]^{20}_{D}$ = 43.2 (*c* = 0.01, $CHCl_3$: MeOH 2:1).

Supplementary Figure 1. HPLC spectra of *rac*-8a

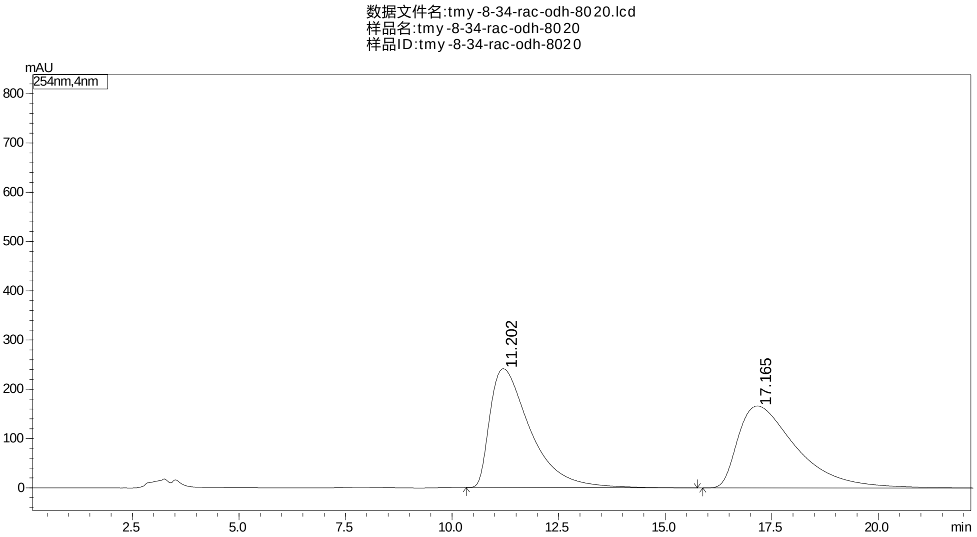

Peak	Ret. Time	Area	Height	Area %	Hight%
1	11.202	16016631	241406	50.070	59.192
2	17.165	15971817	166430	49.930	40.808
total		21346956	316811	100.000	100.000

Supplementary Figure 2. HPLC spectra of (+)-8a

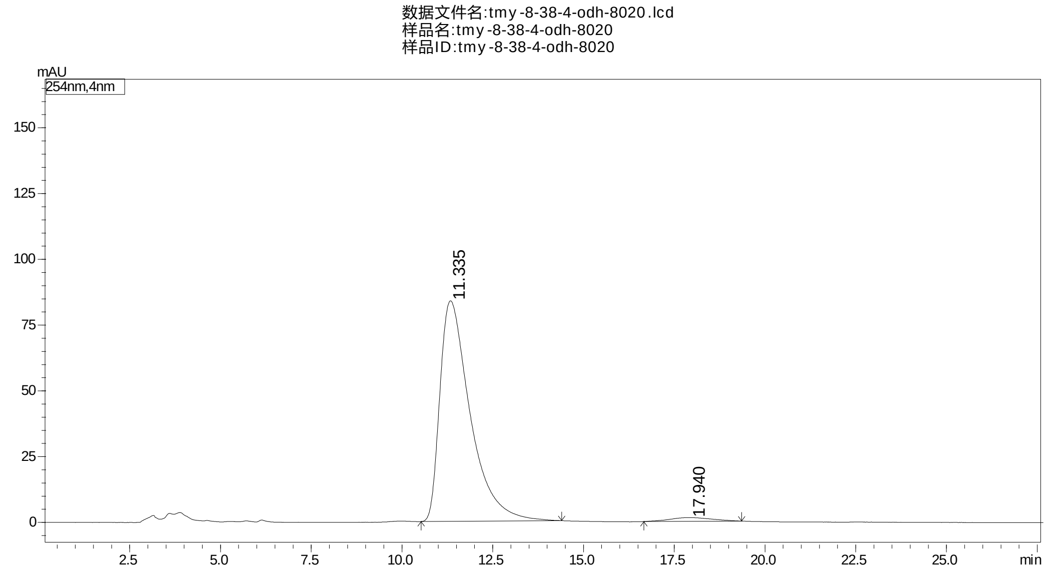

Peak	Ret. Time	Area	Height	Areat%	Hight%
1	11.335	4875701	83848	97.685	98.315
2	17.940	115556	1437	2.315	1.685
total		4991257	85285	100.000	100.000

Supplementary Figure 3. HPLC spectra of *rac*-8b

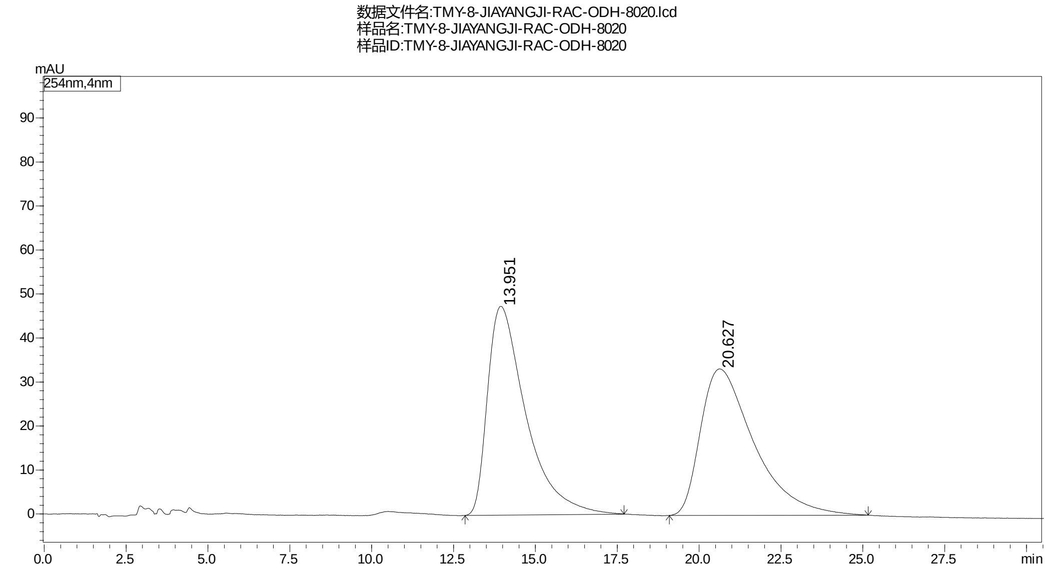

Peak	Ret. Time	Area	Height	Area%	Hight%
1	13.951	3877603	47459	50.563	58.746
2	20.627	63791242	33328	49.437	41.254
total		7668844	80787	100.000	100.000

Supplementary Figure 4. HPLC spectra of (+)-8b

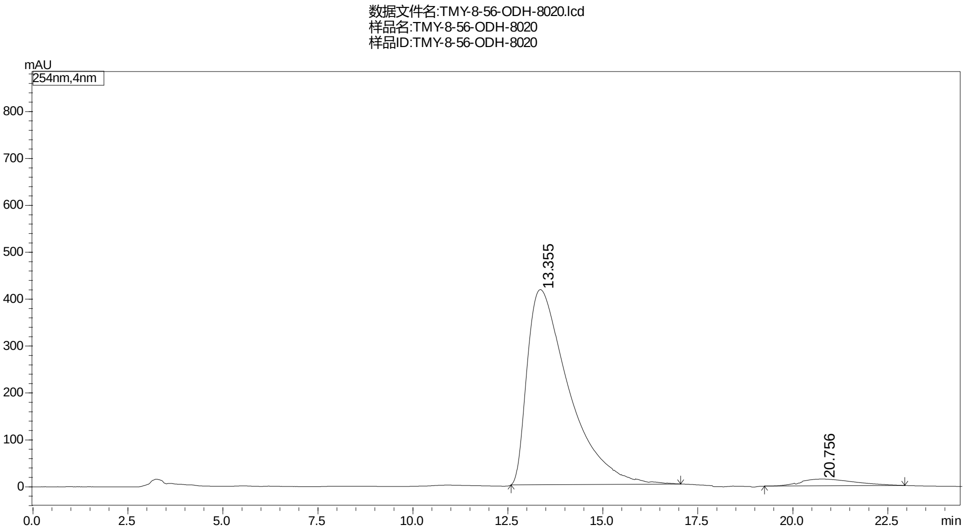

Peak	Ret. Time	Area	Height	Area%	Hight%
1	13.355	31855916	415923	95.726	96.626
2	20.756	1422453	14525	4.274	3.374
total		33278368	430448	100.000	100.000

Supplementary Figure 5. Crude NMR of **8b**, indicating the presence of product **8b** and unreacted **10**

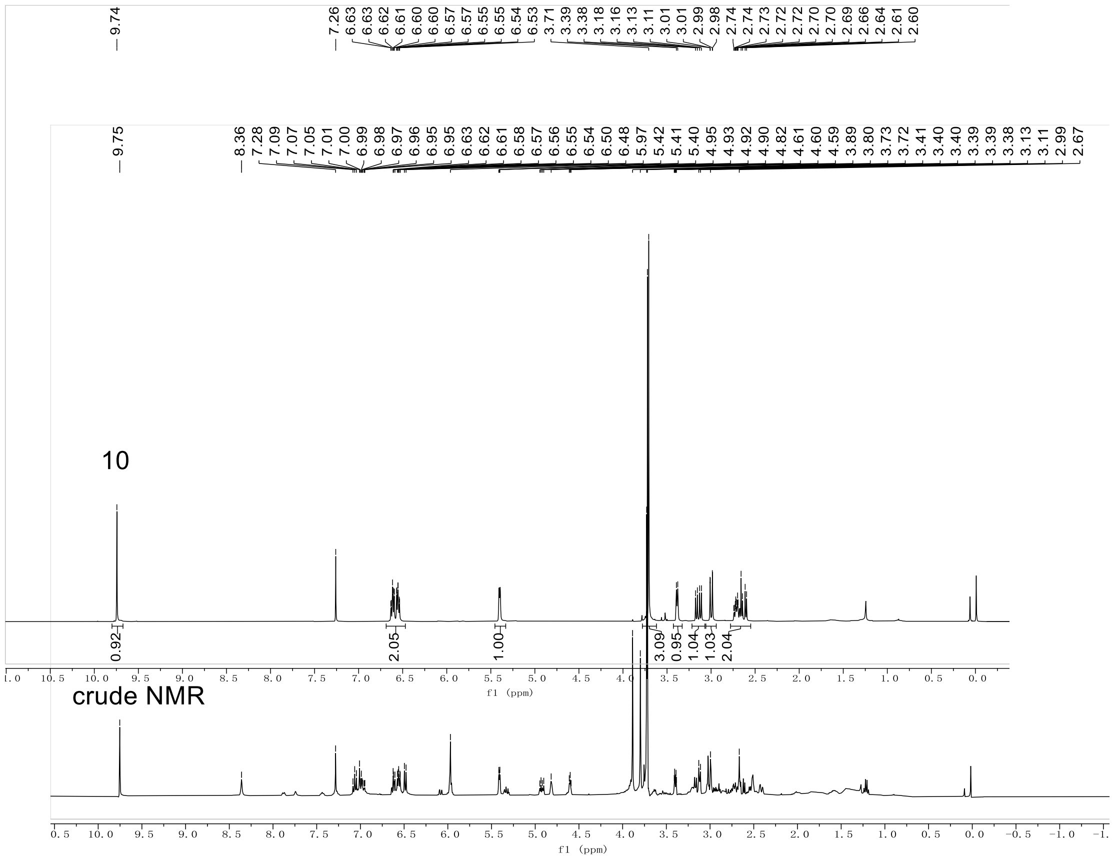

Supplementary Figure 6. HPLC analysis of the recovered **10** (from the reaction of **10** and **5a**; after derivation)

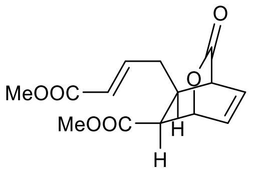

rac

数据文件名:tmy-yanshen-odh-8020-40-rac.2.lcd
样品名:tmy-yanshen-odh-8020-40-rac.2
样品ID:tmy-yanshen-odh-8020-40-rac.2

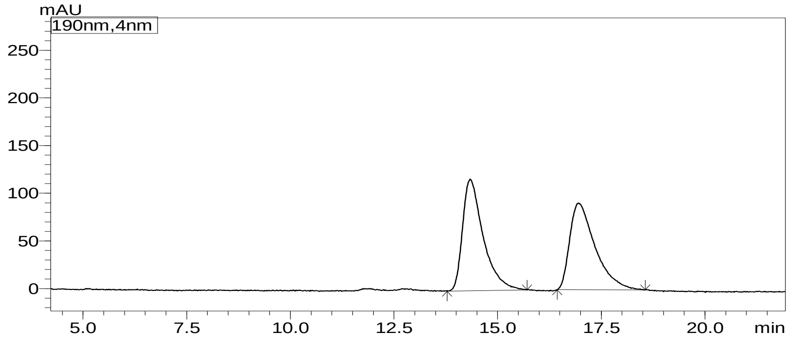

Peak	Ret. Time	Area	Height	Area%
1	14.338	4036037	116818	51.211
2	16.952	3845219	90741	48.789
total		7881256	207559	100.000

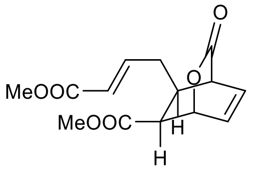

recovered

数据文件名:tmy-zhijieyanshen-odh-8020-40-pure.2.lcd
样品名:tmy-zhijieyanshen-odh-8020-40-pure.2
样品ID:tmy-zhijieyanshen-odh-8020-40.2

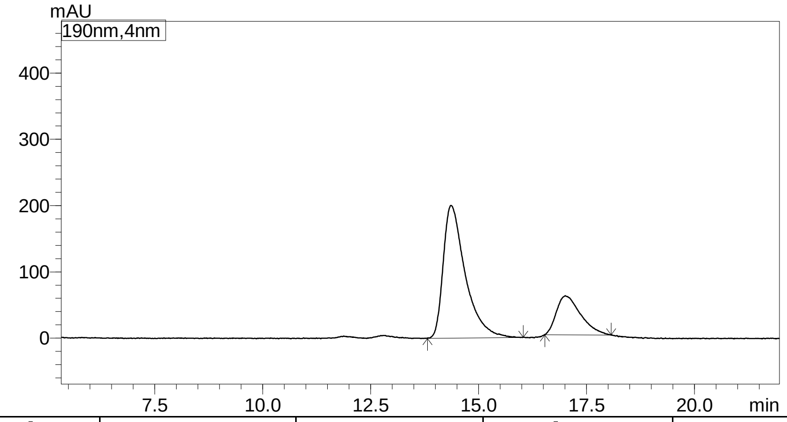

Peak	Ret. Time	Area	Height	Area%
1	14.361	7086308	200106	75.585
2	17.011	2288948	58862	24.415
total		9375256	258968	100.000

Supplementary Figure 7. ^{1}H-NMR of 10's derivative

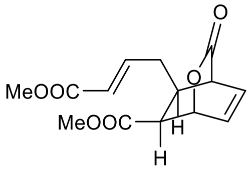

rac

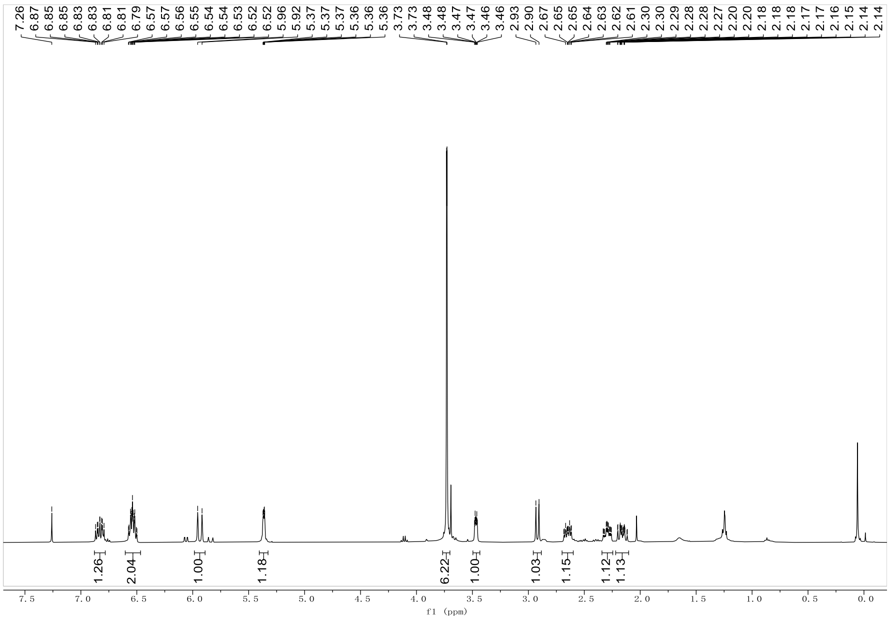

^{1}H NMR (400 MHz, $CDCl_3$) δ 6.87-6.79 (m, 1H), 6.60 – 6.47 (m, 2H), 5.94 (d, *J* = 15.6 Hz, 1H), 5.41 – 5.33 (m, 1H), 3.73 (s, 3H), 3.73 (s, 3H), 3.47 (dt, *J* = 6.0, 2.2 Hz, 1H), 2.92 (d, *J* = 11.2 Hz, 1H), 2.70 – 2.60 (m, 1H), 2.29 (dddd, *J* = 10.8, 7.5, 5.1, 2.5 Hz, 1H), 2.16 (dddd, *J* = 14.5, 10.8, 8.5, 1.2 Hz, 1H).

Supplementary Figure 8. ^{13}C-NMR of 10's derivative

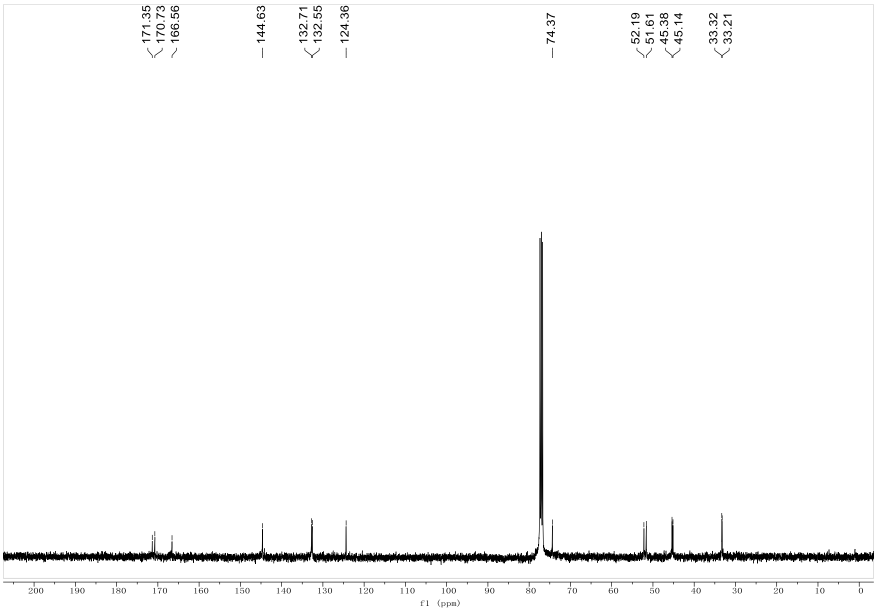

^{13}C NMR (100 MHz, $CDCl_3$) δ 171.35, 170.73, 166.56, 144.63, 132.71, 132.55, 124.36, 74.37, 52.19, 51.61, 45.38, 45.14, 33.32, 33.21.

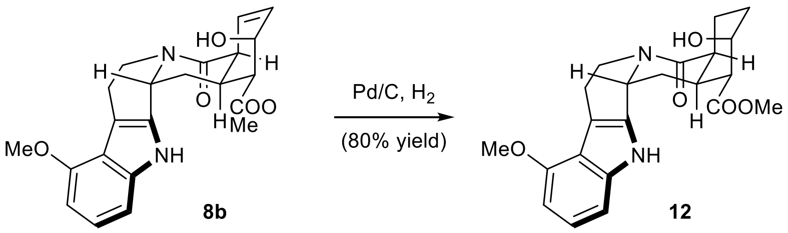

To a stirred solution of **8b** (76 mg, 0.192 mmol) in DCM (3.2 mL) and MeOH (3.2 mL) was added Pd/C (10%) (20 mg, 0.0192mmol). The resulting solution was stirred at rt with a H_2 balloon for 18 hours. The mixture was filtered through a celite pad, which was washed with dichloromethane. The combined filtrate was concentrated under reduced pressure to afford the crude product. The crude product was purified by silica gel column chromatography (DCM / MeOH = 20:1) to afford **12** (62 mg, 80%) as a white powder.

^{1}H NMR (400 MHz, DMSO-d6) δ 10.70 (s, 1H), 6.93 (d, J = 4.6 Hz, 2H), 6.47 – 6.39 (m, 1H), 4.85 (d, J = 6.1 Hz, 1H), 4.72 – 4.61 (m, 2H), 4.25 (t, J = 3.3 Hz, 1H), 3.80 (s, 3H), 3.65 (s, 3H), 2.92 – 2.75 (m, 3H), 2.74 – 2.62 (m, 1H), 2.53 (d, J = 2.2 Hz, 1H), 2.46 (d, J = 14.2 Hz, 1H), 2.28 (dt, J = 12.5, 4.1 Hz, 1H), 2.14 (dt, J = 12.9, 3.9 Hz, 1H), 1.91 (td, J = 12.7, 3.5 Hz, 1H), 1.78 (dd, J = 13.6, 3.6 Hz, 1H), 1.64 – 1.55 (m, 1H), 1.48 (ddd, J = 16.8, 8.4, 3.6 Hz, 1H).
^{13}C NMR (100 MHz, DMSO-d6) δ 172.96, 172.14, 154.09, 137.57, 133.34, 121.96, 117.31, 108.50, 105.23, 99.55, 79.64, 65.17, 55.57, 54.31, 51.79, 47.81, 42.74, 32.53, 30.10, 25.35, 23.23, 20.06.
HRMS-ESI (*m/z*): $[M + H]^+$ calculated for $C_{22}H_{27}N_2O_5$, 399.1920; found 399.1927.
$[\alpha]^{20}_D$ = 60.4 (*c* = 0.005, $CHCl_3$: MeOH 2:1).

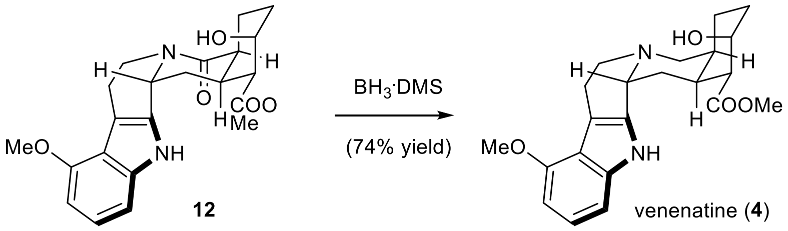

To a stirred solution of **12** (46 mg, 0.11 mmol) in THF (1.2 mL) at 0 °C under N_2 atmosphere was slowly added $BH_3{\cdot}DMS$ (2M in THF) (0.14 ml, 0.28 mmol). The resulting mixture was stirred at 0 °C for 12 hours. Additional $BH_3{\cdot}DMS$ (0.28 mmol) was added, and the reaction was stirred for 6 hours to drive the reaction to complete. The reaction was quenched by the addition of methanol, and warmed to room temperature. Pd/C (20 mg) was added, and the solution was stirred for additional 24 hours to dissociate the B-N complex. The mixture was filtered through a celite pad, which was washed with dichloromethane/methanol. Evaporation of the solvent under reduced pressure afforded the crude product, which was purified by silica gel column chromatography (DCM / MeOH = 20:1) to afford **4** (32 mg, 74%) as a colorless powder.

^{1}**H NMR** (400 MHz, $CDCl_3$) δ 7.97 (s, 1H), 7.03 (t, J = 7.9 Hz, 1H), 6.97 (d, J = 8.1 Hz, 1H), 6.48 (d, J = 7.6 Hz, 1H), 4.44 (s, 1H), 4.35 (d, J = 2.9 Hz, 1H), 3.89 (s, 3H), 3.83 (s, 3H), 3.28 – 3.12 (m, 3H), 3.07 (dd, J = 11.6, 4.2 Hz, 1H), 2.94 – 2.79 (m, 2H), 2.60 (dd, J = 11.6, 2.1 Hz, 1H), 2.49 – 2.43 (m, 1H), 2.38 (td, J = 13.2, 3.6 Hz, 1H), 2.12 – 2.06 (m, 1H), 2.03 – 1.99 (m, 1H), 1.97 – 1.88 (m, 1H), 1.60 (d, J = 8.4 Hz, 1H), 1.52 – 1.40 (m, 1H), 1.39 – 1.31 (m, 1H).
^{13}**C NMR** (100 MHz, $CDCl_3$) δ 174.68, 154.32, 137.18, 129.97, 122.14, 117.73, 107.57, 104.55, 99.74, 65.82, 55.26, 54.42, 51.98, 51.37, 50.42, 49.54, 36.54, 32.17, 30.70, 25.61,19.8, 18.9.
HRMS-ESI (*m/z*): $[M + H]^+$ calculated for $C_{22}H_{29}N_2O_4$, 385.2127; found 385.2126.
$[\alpha]^{20}_{D}$ = 32.7 (*c* = 0.01, $CHCl_3$: MeOH 2:1).

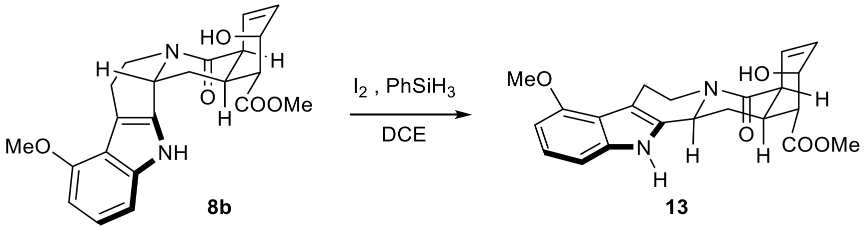

A solution of I_2 (75 mg, 0.3 mmol) and $PhSiH_3$ (75 µl, 0.6 mmol) in DCE (3 mL) was stirred for 15 min. **8b** (117mg, 0.3mmol) was added to the above solution. After being stirred at room temperature for 30 min, the solvent was removed under reduced pressure to afford the crude residue, which was purified by silica gel column chromatography (DCM / MeOH = 20:1) to afford **13** (82 mg, 70%) as a colorless powder.

^{1}**H NMR** (400 MHz, DMSO-d6) δ 10.98 (s, 1H), 6.97 – 6.86 (m, 2H), 6.43 (dd, J = 7.1, 1.5 Hz, 1H), 5.80 (ddd, J = 10.0, 4.9, 3.1 Hz, 1H), 5.65 (d, J = 9.8 Hz, 1H), 5.06 (d, J = 5.0 Hz, 1H), 4.90 – 4.79 (m, 1H), 4.73 (d, J = 9.4 Hz, 1H), 4.41-4.37(m , 1H), 3.80 (s, 3H), 3.72 (s, 3H), 3.18 (s, 1H), 3.00 – 2.89 (m, 1H), 2.85 – 2.78 (m, 2H), 2.77 (d, J = 3.5 Hz, 1H), 2.64-2.72 (m, 2H), 2.52-2.55 (m, 1H).
^{13}**C NMR** (100 MHz, DMSO-d6) δ 172.70, 169.23, 154.30, 138.08, 133.11, 129.88, 127.88, 122.12, 116.57, 106.69, 105.22, 99.64, 79.65, 62.30, 55.58, 54.77, 51.75, 46.62, 45.34, 29.34, 28.39, 23.37.
HRMS-ESI (*m/z*): $[M + H]^+$ calculated for $C_{22}H_{25}N_2O_5$, 397.1763; found 397.1780.
$[\alpha]^{20}_{D}$ = 30.5 (*c* = 0.005, $CHCl_3$: MeOH 2:1).

The above reaction using AcOH (reflux) led to complex mixtures, see the crude NMR of the reaction below.

Supplementary Figure 9. Crude NMR of the reaction of 8b in refluxed AcOH

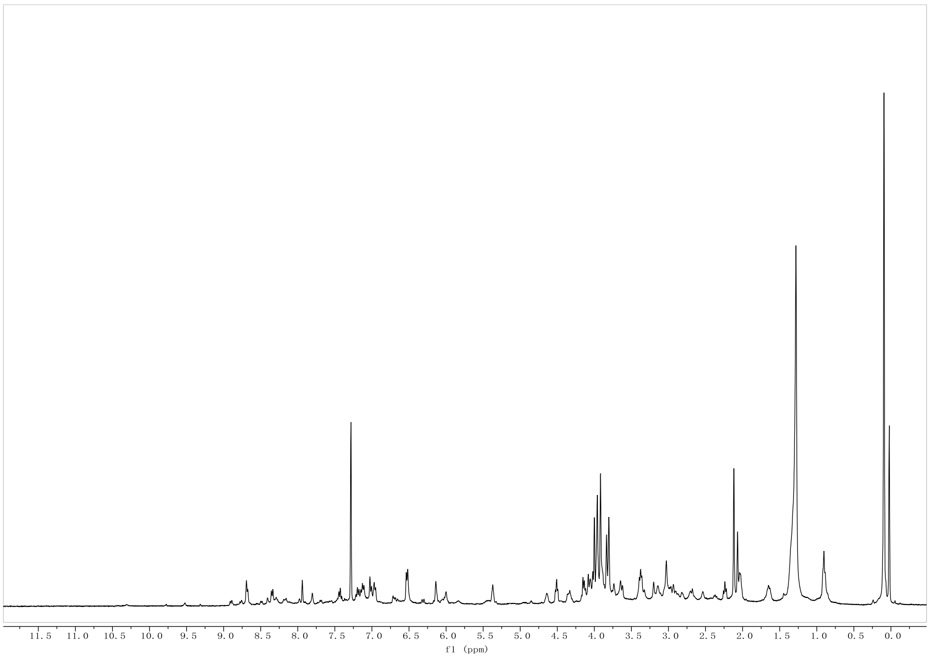

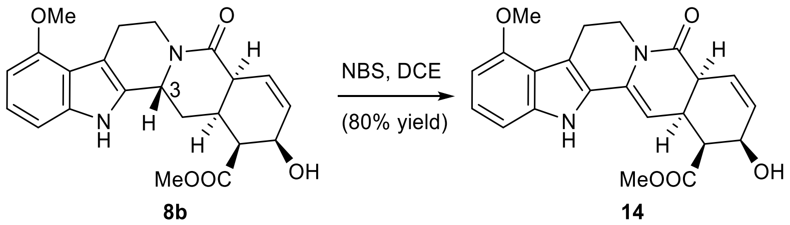

To a stirred solution of **8b** (16 mg, 0.04 mmol) in DCE (0.4 mL) at room temperature was added NBS (7.2 mg, 0.04 mmol). The mixture was stirred for 1 h until the completion of the reaction, as monitored by TLC. The mixture was concentrated under reduced pressure to afford the crude product. The crude product was purified by silica gel column chromatography (DCM / MeOH = 20:1) to afford **14** (13 mg, 80%) as a white powder.

^{1}H NMR (400 MHz, DMSO-d6) δ 11.20 (s, 1H), 7.00 (t, J = 7.9 Hz, 1H), 6.92 (d, J = 8.1 Hz, 1H), 6.45 (d, J = 7.7 Hz, 1H), 5.85 – 5.67 (m, 3H), 5.13 (d, J = 5.1 Hz, 1H), 4.41 (s, 1H), 3.83 (s, 3H), 3.58 (s, 3H), 3.29 – 3.18 (m, 2H), 3.03 (dt, J = 16.3, 5.1 Hz, 1H), 2.98 – 2.85 (m, 2H), 2.57 (s, 2H), 1.24 (s, 2H).
^{13}C NMR (100 MHz, DMSO-d6) δ 179.89, 172.37, 168.95, 154.60, 139.08, 131.49, 127.29, 125.13, 123.82, 116.43, 109.94, 105.15, 101.30, 99.78, 64.32, 55.50, 51.48, 47.38, 42.36, 29.98, 29.56, 22.62.
HRMS-ESI (*m/z*): $[M + H]^+$ calculated for $C_{22}H_{25}N_2O_5$, 395.1607; found 395.1602

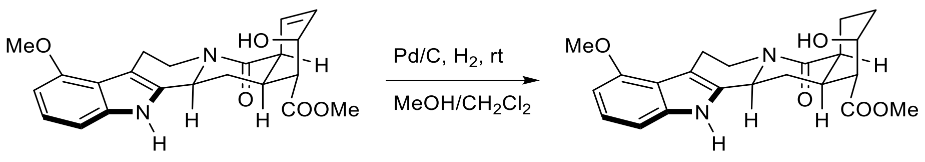

To a stirred solution of **13** (40.6 mg, 0.1 mmol) in DCM/MeOH (1:1, 1.7 mL) at room temperature was added Pd/C (10%) (11 mg, 0.01 mmol). The resulting solution was stirred at room temperature with a H_2 balloon for overnight until the completion of the reaction, as monitored by TLC. The mixture was filtered through a celite pad, which was washed with dichloromethane/Methanol. The filtrate was concentrated under reduced pressure to afford the crude product. The crude product was purified by silica gel column chromatography (DCM / MeOH = 20:1) to afford **13'** (35 mg, 86%) as a white powder.

^{1}H NMR (400 MHz, $CDCl_3$) δ 7.98 (s, 1H), 7.07 (t, J = 8.0 Hz, 1H), 6.92 (d, J = 8.1 Hz, 1H), 6.50 (d, J = 7.8 Hz, 1H), 5.09 (ddd, J = 12.7, 5.0, 1.5 Hz, 1H), 4.69 – 4.61 (m, 1H), 4.34 (s, 1H), 3.90 (s, 3H), 3.85 (s, 3H), 3.22 (s, 1H), 3.08 (dd, J = 16.0, 4.4 Hz, 1H), 2.98 (tdd, J = 16.0, 5.0, 2.5 Hz, 1H), 2.83 (td, J = 12.1, 4.3 Hz, 1H), 2.74 – 2.54 (m, 3H), 2.53 – 2.41 (m, 1H), 2.23 – 2.14 (m, 1H), 2.03 – 1.81 (m, 3H), 1.57 – 1.46 (m, 1H).
^{13}C NMR (100 MHz, $CDCl_3$)δ 174.74, 171.30, 154.63, 137.61, 131.19, 122.91, 116.95, 109.12, 104.26, 100.04, 65.37, 55.25, 54.53, 52.17, 47.99, 43.78, 40.29, 32.02, 31.31, 28.74, 22.95, 20.14.
HRMS-ESI (*m/z*): $[M + H]^+$ calculated for $C_{22}H_{27}N_2O_5$, 399.1920; found 399.1927.
$[\alpha]^{20}_D$ = 29.8 (*c* = 0.01, $CHCl_3$: MeOH 2:1).

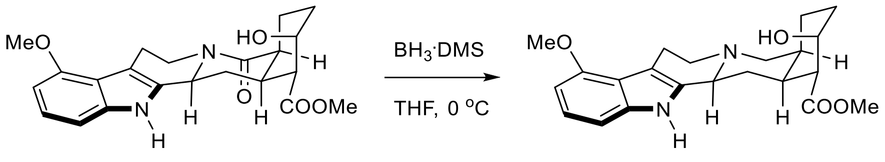

To a stirred solution of **13’** (35 mg, 0.09 mmol) in THF (0.94 mL) at 0 °C under N_2 atmosphere was slowly added BH_3·DMS (2M in THF) (0.12 ml, 0.22 mmol). The resulting mixture was stirred at 0 °C for 12 hours. Additional BH_3·DMS (0.22 mmol) was added, and the reaction was stirred for 6 hours to drive the reaction to complete. The reaction was quenched by the addition of methanol, and warmed to room temperature. Pd/C (20 mg) was added, and the solution was stirred for additional 24 hours to dissociate the B-N complex. The mixture was filtered through a celite pad, which was washed with dichloromethane. Evaporation of the solvent under reduced pressure afforded the crude product, which was purified by silica gel column chromatography (DCM / MeOH = 20:1) to afford **3** (26 mg, 77%) as a colorless powder.

^{1}H NMR (400 MHz, $CDCl_3$) δ 7.71 (s, 1H), 6.99 (t, J = 7.9 Hz, 1H), 6.89 (d, J = 8.1 Hz, 1H), 6.45 (d, J = 7.7 Hz, 1H), 4.27 (d, J = 2.8 Hz, 1H), 3.87 (s, 3H), 3.83 (s, 3H),

3.17 – 3.04 (m, 1H), 3.04 (d, J = 10.8 Hz, 1H), 3.00 – 2.90 (m, 2H), 2.87 (d, J = 11.3 Hz, 1H), 2.63 – 2.57 (m, 1H), 2.56 (dd, J = 11.2, 3.3 Hz, 1H), 2.49 (dt, J = 11.7, 5.8 Hz, 1H), 2.43 – 2.28 (m, 3H), 1.98 (dd, J = 13.9, 3.3 Hz, 1H), 1.73 (d, J = 13.0 Hz, 1H), 1.65 (d, J = 11.1 Hz, 2H), 1.46 (tt, J = 14.1, 3.9 Hz, 2H), 1.33 (d, J = 10.8 Hz, 1H).
^{13}C NMR (100 MHz, $CDCl_3$) δ 175.7, 154.5, 137.3, 133.1, 121.9, 117.6, 108.1, 104.3, 99.8, 65.7, 61.5, 60.8, 55.3, 53.7, 52.0, 49.7, 37.5, 36.8, 31.8, 29.4, 23.8, 20.5.
HRMS-ESI (m/z): [M + H]+ calculated for $C_{22}H_{29}N_2O_4$, 385.2127; found 385.2126.
$[\alpha]^{20}_D$ = -17.8 (*c* = 0.01, $CHCl_3$: MeOH 2:1).

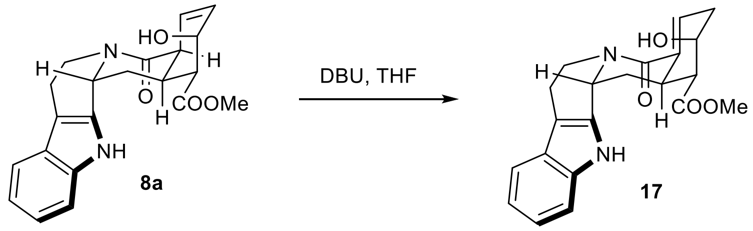

To a stirred solution of **8a** (36.6 mg, 0.1 mmol) in dry THF (1.5 mL) under N_2 atmosphere was added DBU (75 µl, 0.5 mmol). The reaction was stirred at 50 °C for 3.5 h. The reaction was quenched with 2N HCl until pH = 6 and concentrated under reduced pressure. The residue was diluted with water (10 mL) and extracted with ethyl acetate (3 x 10 mL). The combined organic phase was washed with water (10 mL) and brine (10 mL), dried over Na_2SO_4, and concentrated under reduced pressure to give the crude residue. The crude product was purified by silica gel column chromatography DCM / MeOH = 20:1) to afford the product **17** (35 mg, 95 % yield) as a white powder.

^{1}H NMR (400 MHz, DMSO-d6) δ 11.03 (s, 1H), 7.35 (dd, J = 11.5, 7.9 Hz, 2H), 7.07 (t, J = 7.5 Hz, 1H), 6.97 (t, J = 7.4 Hz, 1H), 6.75 (s, 1H), 5.16 (d, J = 3.7 Hz, 1H), 5.02 (s, 1H), 4.79 (dd, J = 12.6, 5.7 Hz, 1H), 3.86 (dq, J = 9.3, 4.6 Hz, 1H), 3.65 (s, 3H), 3.10 – 2.95 (m, 2H), 2.85 – 2.70 (m, 1H), 2.70 – 2.57 (m, 2H), 2.48 – 2.27 (m, 2H), 1.81 (td, J = 13.6, 5.8 Hz, 1H).
^{13}C NMR (100 MHz, DMSO) δ 172.05, 163.58, 136.28, 134.66, 134.15, 128.82, 127.42, 121.44, 119.15, 118.04, 111.83, 109.24, 65.78, 53.60, 51.40, 49.57, 43.35, 32.38, 31.83, 27.43, 21.09.
HRMS-ESI (*m/z*): $[M + H]^+$ calculated for $C_{21}H_{23}N_2O_4$, 367.1658; found 367.1650.

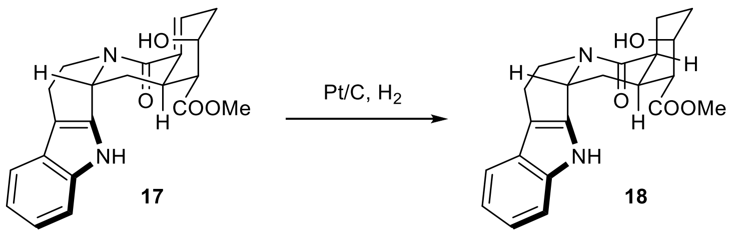

To a stirred solution of **17** (14.3 mg, 0.04 mmol) in EtOH (1.2mL) and THF (0.4 ml) at room temperature was added Pt/C (10 %) (16 mg, 0.2 mmol). The mixture was stirred for overnight with a H_2 balloon. The mixture was filtered through a celite pad, which was washed with dichloromethane. The filtrate was concentrated under reduced

pressure to afford the crude product. The crude product was purified by silica gel column chromatography (DCM / MeOH = 20:1) to afford **18** (5 mg, 33%) as a white powder and recovered **18** (9 mg, 62%).

^{1}H NMR (400 MHz, DMSO-d6) δ 10.75 (s, 1H), 7.35 (t, J = 8.7 Hz, 2H), 7.10 – 7.01 (m, 1H), 7.01 – 6.92 (m, 1H), 4.90 (d, J = 6.2 Hz, 1H), 4.78 – 4.64 (m, 2H), 4.26 (s, 1H), 3.67 (s, 3H), 2.88 (td, J = 12.0, 4.1 Hz, 1H), 2.83 – 2.64 (m, 2H), 2.62 – 2.50 (m, 8H), 2.29 (dt, J = 12.8, 4.4 Hz, 1H), 2.21 – 2.09 (m, 1H), 2.02 – 1.86 (m, 1H), 1.79 (dd, J = 13.6, 3.5 Hz, 1H), 1.65 – 1.55 (m, 1H), 1.49 (td, J = 12.8, 6.5 Hz, 1H).
^{13}C NMR (100 MHz, DMSO-d6) δ 172.95, 172.21, 136.31, 135.27, 127.31, 121.23, 118.94, 117.90, 111.67, 108.94, 65.18, 54.40, 51.80, 47.80, 44.09, 42.51, 32.55, 30.14, 25.27, 21.11, 20.07.
HRMS-ESI (*m/z*): $[M + H]^+$ calculated for $C_{21}H_{25}N_2O_4$, 369.1814; found 369.1808.

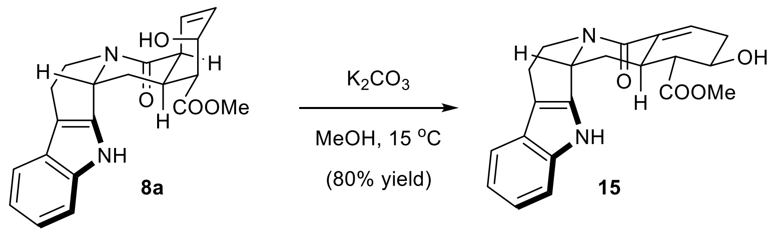

To a stirred solution of **8a** (1.74 g, 4.75 mmol) in MeOH (67 ml) K_2CO_3 was added at 15 °C. The mixture was stirred for 24 hours. The mixture was quenched with 1N HCl at 0 °C until pH = 6 and concentrated under reduced pressure. The residue was diluted with water and extracted with ethyl acetate (6 x 50 mL). The combined organic phase was washed with water (50 mL) and brine (50 mL), dried over Na_2SO_4, and concentrated under reduced pressure to give the crude residue. The crude product was purified by silica gel column chromatography DCM / MeOH = 20:1) to afford the product **15** (1.39 g, 80 % yield) as a white powder.

^{1}H NMR (400 MHz, DMSO-d6) δ 10.98 (s, 1H), 7.36 (dd, J = 16.0, 7.9 Hz, 2H), 7.07 (t, J = 7.3 Hz, 1H), 6.98 (t, J = 7.4 Hz, 1H), 6.72 – 6.60 (m, 1H), 5.14 (d, J = 6.0 Hz, 1H), 5.02 (d, J = 3.2 Hz, 1H), 4.80 (dd, J = 12.5, 5.4 Hz, 1H), 3.74 (s, 3H), 3.72 – 3.63 (m, 1H), 2.99 (td, J = 12.2, 4.5 Hz, 1H), 2.84 – 2.70 (m, 1H), 2.65 – 2.51 (m, 3H), 2.50 – 2.42 (m, 1H), 2.41 – 2.24 (m, 2H), 2.16 – 2.03 (m, 1H), 1.92 (td, J = 13.3, 5.9 Hz, 1H).
^{13}C NMR (100 MHz, DMSO-d6) δ 173.89, 163.06, 136.27, 134.44, 132.88, 130.68, 127.29, 121.48, 119.13, 118.07, 111.78, 109.23, 67.21, 55.11, 53.57, 52.14, 43.32, 35.18, 34.60, 29.78, 21.22.
HRMS-ESI (*m/z*): $[M + H]^+$ calculated for $C_{21}H_{25}N_2O_4$, 369.1814; found 369.1808
$[\alpha]^{20}_D = 40.8$ (*c* = 0.005, $CHCl_3$: MeOH 2:1).

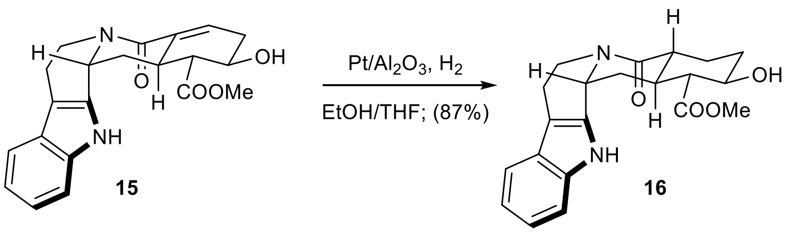

To a stirred solution of **15** (168 mg, 0.46 mmol) in EtOH (12 mL) and THF (8 mL) at room temperature was added Pt/Al_2O_3(5%) (358 mg, 0.092 mmol). The mixture was stirred for overnight with a H_2 balloon. The mixture was filtered through a celite pad, which was washed with dichloromethane. The filtrate was concentrated under reduced pressure to afford the crude product. The crude product was purified by silica gel column chromatography (DCM / MeOH = 20:1) to afford **16** (147 mg, 87%) as a white powder (dr > 15:1).

16:^{1}H NMR (400 MHz, DMSO-d6) δ 10.87 (s, 1H), 7.37 (d, J = 7.8 Hz, 1H), 7.31 (d, J = 8.0 Hz, 1H), 7.10 – 7.02 (m, 1H), 7.02 – 6.93 (m, 1H), 4.99 (d, J = 6.1 Hz, 1H), 4.88 (d, J = 5.7 Hz, 1H), 4.67 (dd, J = 12.7, 4.7 Hz, 1H), 3.69 (s, 3H), 3.46 – 3.34 (m, 1H), 2.89 (td, J = 12.1, 4.3 Hz, 1H), 2.73 (dddd, J = 14.2, 11.7, 5.2, 2.3 Hz, 1H), 2.57 (dd, J = 15.4, 4.1 Hz, 1H), 2.25 (dt, J = 13.6, 2.8 Hz, 1H), 2.19 – 2.07 (m, 2H), 2.10 – 1.98 (m, 2H), 1.89 – 1.80 (m, 1H), 1.41 (qd, J = 11.5, 3.1 Hz, 1H), 1.32 – 1.18 (m, 1H), 1.15 – 1.00 (m, 1H).

13C NMR (100 MHz, DMSO) δ 173.83, 170.81, 136.31, 134.94, 127.22, 121.40, 119.05, 118.01, 111.68, 109.01, 70.61, 57.72, 53.54, 51.91, 44.12, 42.44, 35.83, 34.44, 30.81, 25.42, 20.88.

HRMS-ESI (*m/z*): $[M + H]^+$ calculated for $C_{21}H_{25}N_2O_4$, 369.1814; found 369.1808.

$[\alpha]^{20}_D$ = -11.2 (*c* = 0.01, $CHCl_3$: MeOH 2:1).

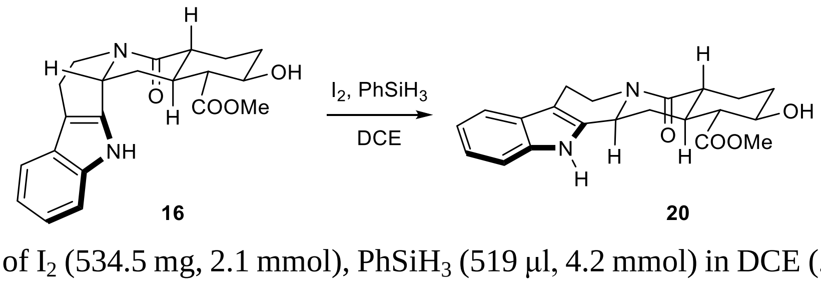

A solution of I_2 (534.5 mg, 2.1 mmol), $PhSiH_3$ (519 μl, 4.2 mmol) in DCE (21 mL) was stirred for 15 min. Then **16** (775 mg, 2.1mmol) was added to the stirred solution at rt. The resulting mixture was stirred for 24 h. Evaporation of the solvent under reduced pressure afforded the crude product, which was purified by silica gel column chromatography (DCM /MeOH = 20:1) to afford **20** (679 mg, 88%) as a white powder.

^{1}H NMR (400 MHz, DMSO-d6) δ 10.95 (s, 1H), 7.40 (d, J = 7.8 Hz, 1H), 7.32 (d, J = 8.0 Hz, 1H), 7.11 – 7.02 (m, 1H), 7.02 – 6.93 (m, 1H), 4.95 (d, J = 5.3 Hz, 1H), 4.91 (dd, J = 12.7, 4.6 Hz, 1H), 4.76 (dd, J = 11.9, 4.7 Hz, 1H), 3.75 (s, 3H), 3.65 – 3.53 (m, 1H), 2.76 (td, J = 12.2, 4.0 Hz, 1H), 2.73 – 2.64 (m, 1H), 2.65 – 2.52 (m, 1H), 2.44 –

2.35 (m, 1H), 2.30 – 2.22 (m, 1H), 2.15 (t, J = 10.4 Hz, 1H), 2.01 – 1.91 (m, 2H), 1.85 (qd, J = 11.5, 2.0 Hz, 1H), 1.46 (q, J = 12.1 Hz, 1H), 1.35 – 1.24 (m, 3H).
^{13}C NMR (100 MHz, DMSO-d6) δ 174.54, 169.20, 136.61, 134.93, 126.76, 121.47, 119.06, 118.23, 111.53, 107.40, 71.07, 57.20, 53.95, 51.88, 44.75, 37.38, 34.86, 32.83, 25.98, 21.29.
HRMS-ESI (*m/z*): $[M + H]^+$ calculated for $C_{21}H_{25}N_2O_4$, 369.1814; found 369.1808.
$[\alpha]^{20}_D$ = 57.2 (*c* = 0.01, $CHCl_3$: MeOH 2:1).

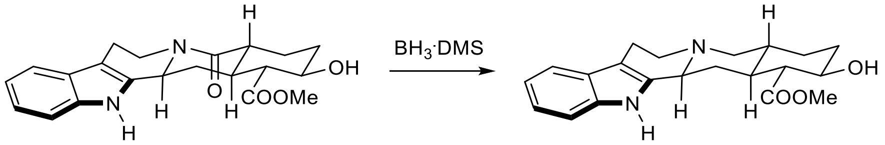

20 **1b**

To a stirred solution of **20** (192 mg, 0.522 mmol) in THF (10.6 mL) at 0 °C under N_2 atmosphere was slowly added BH_3·DMS (2M in THF) (0.652 ml, 1.3 mmol). The resulting mixture was stirred at 0 °C for 12 hours. Additional BH_3·DMS (1.3 mmol) was added, and the reaction was stirred for 6 hours to drive the reaction to complete. The reaction was quenched by the addition of methanol, and warmed to room temperature. Pd/C (70 mg) was added, and the solution was stirred for additional 24 hours to dissociate the B-N complex. The mixture was filtered through a celite pad, which was washed with dichloromethane. Evaporation of the solvent under reduced pressure afforded the crude product, which was purified by silica gel column chromatography (DCM / MeOH = 20:1) to afford **1b** (137 mg, 74%) as a colorless powder.

^{1}H NMR (400 MHz, $CDCl_3$) δ 7.83 (s, 1H), 7.46 (d, J = 7.7 Hz, 1H), 7.30 (d, J = 7.9 Hz, 1H), 7.13 (td, J = 7.6, 1.4 Hz, 1H), 7.08 (td, J = 7.4, 1.2 Hz, 1H), 3.89 – 3.85 (m, 1H), 3.83 (s, 3H), 3.23 (d, J = 9.6 Hz, 1H), 3.12 – 3.04 (m, 1H), 3.04 – 2.93 (m, 2H), 2.75 – 2.67 (m, 1H), 2.60 (td, J = 11.1, 4.4 Hz, 1H), 2.24 – 2.05 (m, 4H), 1.93 (dt, J = 12.1, 2.5 Hz, 1H), 1.70 (m, 1H), 1.53 (m, 2H), 1.48 – 1.38 (m, 2H), 1.22 – 1.14 (m, 1H).
^{13}C NMR (100 MHz, $CDCl_3$) δ 174.93, 136.06, 134.22, 127.38, 121.44, 119.44, 118.16, 110.79, 108.33, 72.25, 61.09, 59.50, 57.35, 52.95, 51.94, 41.96, 39.91, 34.18, 34.16, 29.70, 27.91, 21.72.
HRMS-ESI (*m/z*): $[M + H]^+$ calculated for $C_{21}H_{27}N_2O_3$, 355.2022; found 355.2018.
$[\alpha]^{20}_D$ = 14.4 (*c* = 0.01, EtOH).

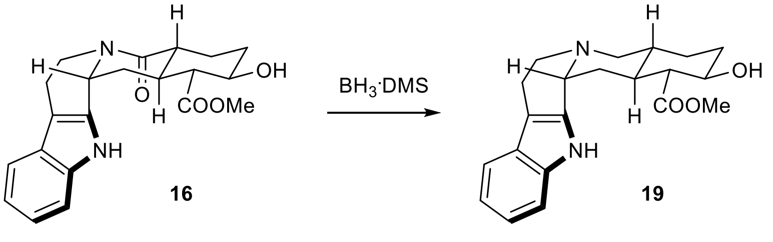

16 **19**

To a stirred solution of **16** (85 mg, 0.25 mmol) in THF (5 mL) under N_2 atmosphere was slowly added BH_3·DMS (2M in THF) (0.5 ml, 1.25 mmol). The resulting mixture was stirred at room temperature for 12 hours. Additional BH_3·DMS (0.625 mmol) was

added, and the reaction was stirred for 6 hours to drive the reaction to complete. The reaction was quenched by the addition of methanol, and warmed to room temperature. Pd/C (40 mg) was added, and the solution was stirred for additional 24 hours to dissociate the B-N complex. The mixture was filtered through a celite pad, which was washed with dichloromethane. Evaporation of the solvent under reduced pressure afforded the crude product, which was purified by silica gel column chromatography (DCM / MeOH = 20:1) to afford **19** (52mg, 60%) as a white powder.

^{1}H NMR (400 MHz, $CDCl_3$) δ 7.86 (s, 1H), 7.48 (d, J = 7.7 Hz, 1H), 7.39 (d, J = 8.1 Hz, 1H), 7.22 – 7.13 (m, 1H), 7.11 (td, J = 7.4, 1.2 Hz, 1H), 4.45 (d, J = 2.5 Hz, 1H), 3.83 (s, 3H), 3.73 – 3.66 (m, 1H), 3.60 (td, J = 10.7, 4.4 Hz, 1H), 3.33 – 3.24 (m, 2H), 3.00 (dtd, J = 18.2, 9.6, 8.9, 2.5 Hz, 1H), 2.66 – 2.52 (m, 2H), 2.42 (t, J = 11.0 Hz, 2H), 2.14 (t, J = 10.6 Hz, 1H), 2.07 – 1.95 (m, 2H), 1.84 – 1.72 (m, 1H), 1.73 – 1.66 (m, 1H), 1.59 (dq, J = 13.2, 3.5 Hz, 1H), 1.46 – 1.30 (m, 2H), 1.31 – 1.17 (m, 2H), 0.94 (qd, J = 13.2, 3.6 Hz, 1H).
^{13}C NMR (100 MHz, CDCl3) δ 175.02, 135.84, 132.55, 127.74, 121.53, 119.49, 118.01, 111.27, 107.98, 71.97, 62.75, 56.77, 53.60, 51.89, 51.04, 39.49, 36.73, 34.15, 32.10, 27.86, 16.88.
HRMS-ESI (*m/z*): $[M + H]^+$ calculated for $C_{21}H_{27}N_2O_3$, 355.2022; found 355.2018.
$[\alpha]^{20}_D$ = 53.2 (*c* = 0.01, $CHCl_3$: MeOH 2:1).

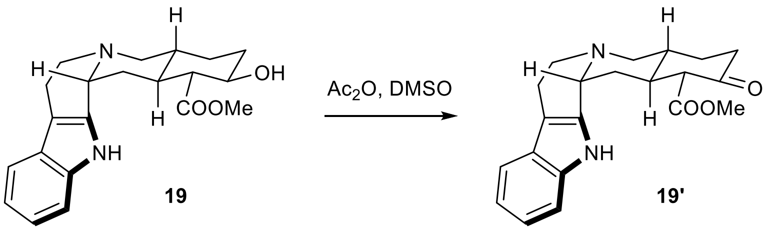

19 19'

To a stirred solution of **19** (27 mg, 0.076 mmol) in DMSO (6.3 mL) under N_2 atmosphere was added Ac_2O (2.1 ml). The resulting mixture was stirred at room temperature for 2 hours and then was quenched with aqueous $NaHCO_3$. The reaction was extracted with ethyl acetate (6 x 50 mL). The combined organic phase was washed with water (50 mL) and brine (50 mL), dried over Na_2SO_4, and concentrated under reduced pressure to give the crude residue. The crude product was purified by silica gel column chromatography DCM / MeOH = 20:1) to afford the product **19'** (23 mg, 86 % yield) as a white powder.
^{1}H NMR (400 MHz, $CDCl_3$) δ 8.03 (s, 1H), 7.48 (d, J = 7.8 Hz, 1H), 7.42 (d, J = 8.0 Hz, 1H), 7.19 (t, J = 7.6 Hz, 1H), 7.12 (t, J = 7.4 Hz, 1H), 4.56 (s, 1H), 3.84 (s, 3H), 3.41 – 3.33 (m, 2H), 3.23 (d, J = 12.3 Hz, 1H), 3.09 – 2.95 (m, 1H), 2.81 (dd, J = 11.3, 3.6 Hz, 1H), 2.67 (d, J = 14.7 Hz, 1H), 2.53 (t, J = 11.1 Hz, 1H), 2.47 – 2.37 (m, 2H), 2.24 (dt, J = 13.5, 2.8 Hz, 1H), 2.07 – 1.86 (m, 4H), 1.76 (td, J = 11.6, 2.8 Hz, 1H).
^{13}C NMR (100 MHz, $CDCl_3$)δ 204.47, 170.01, 136.09, 131.40, 127.43, 121.95, 119.71, 118.06, 111.54, 107.61, 61.38, 53.53, 52.31, 50.81, 50.40, 40.78, 38.71, 38.30, 32.82, 30.01, 29.73, 16.81
HRMS-ESI (*m/z*): $[M + H]^+$ calculated for $C_{21}H_{25}N_2O_3$, 353.1865; found 353.1862.

[α]$^{20}_{D}$ = 56.6 (c = 0.01, $CHCl_3$: MeOH 2:1).

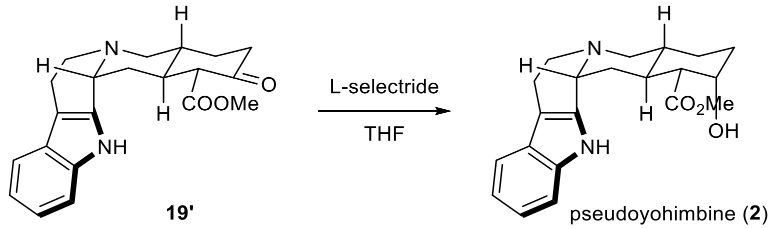

To a stirred solution of **19'** (23 mg, 0.065 mmol) in THF (10 mL) under N_2 atmosphere was added dropwisely L-selectride (1M) (0.1 ml, 0.1 mmol) at -78 °C over 5 minutes. After being stirred for 30 min, the reaction was quenched with aqueous NH_4Cl and extracted with ethyl acetate (3x 50 mL). The combined organic phase was washed with water (50 mL) and brine (50 mL), dried over Na_2SO_4, and concentrated under reduced pressure to give the crude residue. The crude product was purified by silica gel column chromatography DCM / MeOH = 20:1) to afford the product **2** (17 mg, 72 % yield) as a white powder.

^{1}H NMR (400 MHz, $CDCl_3$)δ 8.52 (s, 1H), 7.48 (d, J = 7.7 Hz, 1H), 7.44 (d, J = 8.0 Hz, 1H), 7.23 – 7.15 (m, 2H), 7.18 – 7.09 (m, 1H), 4.74 (d, J = 4.4 Hz, 1H), 4.23 (d, J = 2.9 Hz, 1H), 3.78 (s, 3H), 3.47 – 3.37 (m, 2H), 3.03 (dddd, J = 18.3, 10.5, 7.7, 2.4 Hz, 1H), 2.89 – 2.77 (m, 2H), 2.68 (t, J = 11.3 Hz, 1H), 2.39 – 2.31 (m, 1H), 2.26 (dd, J = 11.4, 2.6 Hz, 1H), 2.05 – 1.94 (m, 1H), 1.86 – 1.73 (m, 2H), 1.68 (td, J = 11.6, 2.6 Hz, 1H), 1.51 (tdd, J = 13.8, 4.4, 2.4 Hz, 1H), 1.33 – 1.28 (m, 2H), 1.24 – 1.15 (m, 2H), 0.96 – 0.80 (m, 1H).

^{13}C NMR (100 MHz, $CDCl_3$) δ 174.40, 136.51, 129.55, 126.96, 122.36, 119.95, 115.59, 111.81, 106.63, 67.01, 55.05, 52.07, 51.14, 50.67, 50.56, 38.16, 31.34, 31.12, 30.47, 22.50, 16.45.

HRMS-ESI (m/z): $[M + H]^+$ calculated for $C_{21}H_{27}N_2O_3$, 355.2022; found 355.2018

[α]$^{20}_{D}$ = 19.1 (c = 0.01, $CHCl_3$: MeOH 2:1)

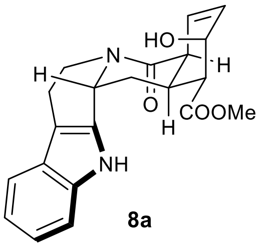

8a

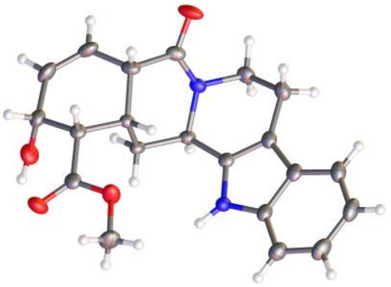

8a was dissolved in DCM / MeOH (1:1) in a vial, and then n-hexane was added slowly to form a two-layer solution. And then wait for crystal precipitation while standing still. CCDC 2287987.

Supplementary Table 2. Crystal data and structure refinement for exp_8415

Identification code	exp_8415
Empirical formula	C21H22N2O4
Formula weight	366.40
Temperature / K	116.60(14)
Crystal system	orthorhombic
Space group	P212121
a / Å, b / Å, c / Å	8.27297(12), 11.15465(18), 19.0948(3)
α/°, β/°, γ/°	90, 90, 90
Volume / Å3	1762.11(5)
Z	4
ρcalc / mg mm-3	1.381
μ / mm-1	0.786
F(000)	776
Crystal size / mm3	0.360 × 0.250 × 0.210
2Θ range for data collection	9.182 to 132.22°
Index ranges	-7 ≤ h ≤ 9, -12 ≤ k ≤ 12, -22 ≤ l ≤ 22
Reflections collected	8572
Independent reflections	3020[R(int) = 0.0303 (inf-0.9Å)]
Data/restraints/parameters	3020/0/247
Goodness-of-fit on F2	1.030
Final R indexes [I>2σ (I) i.e. Fo>4σ (Fo)]	R1 = 0.0338, wR2 = 0.0853
Final R indexes [all data]	R1 = 0.0356, wR2 = 0.0866
Largest diff. peak/hole / e Å-3	0.165/-0.155
Flack Parameters	0.04(13)
Completeness	0.9994

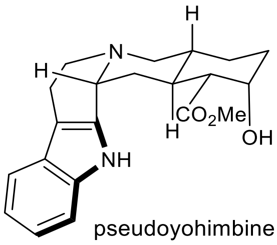

pseudoyohimbine (**2**)

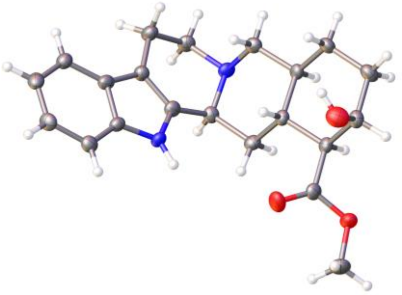

2 was dissolved in DCE in a vial, and then n-hexane was added slowly to form a two-layer solution. And then wait for crystal precipitation while standing still. CCDC 2287988.

Supplementary Table 3. Crystal data and structure refinement for exp_8587

Identification code	exp_8587
Empirical formula	C21H26N2O3
Formula weight	354.44
Temperature / K	114.80(10)
Crystal system	orthorhombic
Space group	P212121
a / Å, b / Å, c / Å	10.0723(8), 10.4529(9), 16.787(3)
α/°, β/°, γ/°	90.00, 90.00, 90.00
Volume / Å3	1767.4(4)
Z	4
ρcalc / mg mm-3	1.332
μ / mm-1	0.089
F(000)	760
Crystal size / mm3	0.26 × 0.22 × 0.13
2Θ range for data collection	6.12 to 51.98°
Index ranges	-12 ≤ h ≤ 8, -12 ≤ k ≤ 12, -20 ≤ l ≤ 10
Reflections collected	6972
Independent reflections	3384[R(int) = 0.0645 (inf-0.9Å)]
Data/restraints/parameters	3384/0/237
Goodness-of-fit on F2	1.035
Final R indexes [I>2σ (I) i.e. Fo>4σ (Fo)]	R1 = 0.0679, wR2 = 0.1148
Final R indexes [all data]	R1 = 0.1118, wR2 = 0.1416
Largest diff. peak/hole / e Å-3	0.332/-0.288
Flack Parameters	-1(2)
Completeness	0.9957

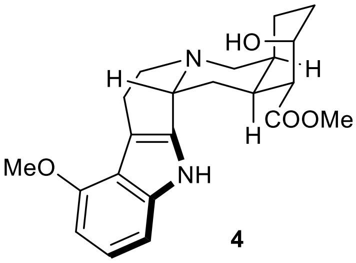

4

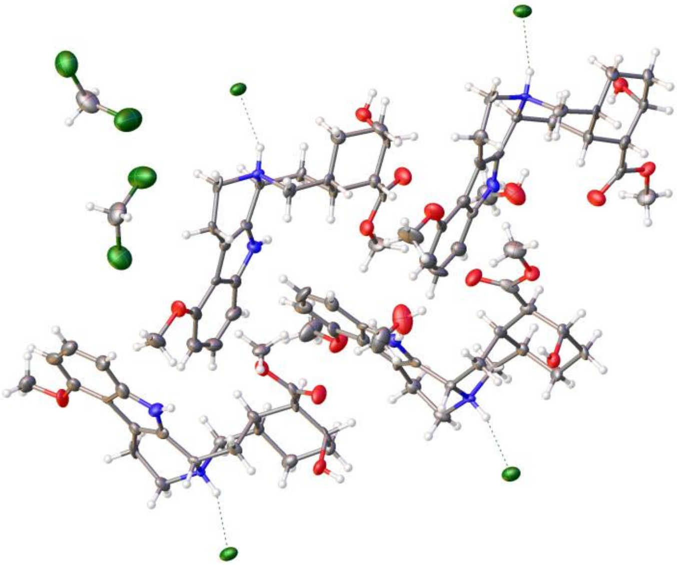

4 was dissolved in DCE in a vial, and then n-hexane was added slowly to form a two-layer solution. And then wait for crystal precipitation while standing still. CCDC 2322036.

Supplementary Table 4. Crystal data and structure refinement for exp_8615

Identification code	exp_8615
Empirical formula	C23H32Cl2N2O4.5
Formula weight	479.41
Temperature / K	115.20(14)
Crystal system	triclinic
Space group	P-1
a / Å, b / Å, c / Å	14.4324(12), 18.7614(19), 20.123(3)
α/°, β/°, γ/°	76.804(10), 69.371(10), 70.833(8)
Volume / Å3	4777.6(9)
Z	8
ρcalc / mg mm-3	1.333
μ / mm-1	0.306
F(000)	2032
Crystal size / mm3	0.40 × 0.38 × 0.21
2Θ range for data collection	5.9 to 52°
Index ranges	-16 ≤ h ≤ 17, -23 ≤ k ≤ 23, -24 ≤ l ≤ 23
Reflections collected	46022
Independent reflections	18387[R(int) = 0.0660 (inf-0.9Å)]
Data/restraints/parameters	18387/0/1151
Goodness-of-fit on F2	2.066
Final R indexes [I>2σ (I) i.e. Fo>4σ (Fo)]	R1 = 0.1846, wR2 = 0.3900
Final R indexes [all data]	R1 = 0.2322, wR2 = 0.4151
Largest diff. peak/hole / e Å-3	5.348/-1.110
Flack Parameters	N
Completeness	0.9981

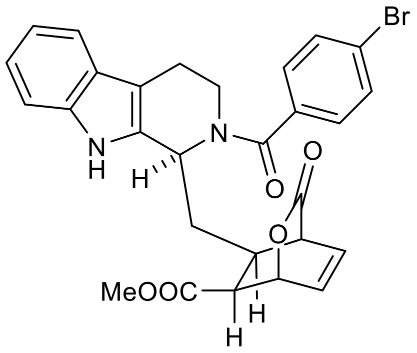

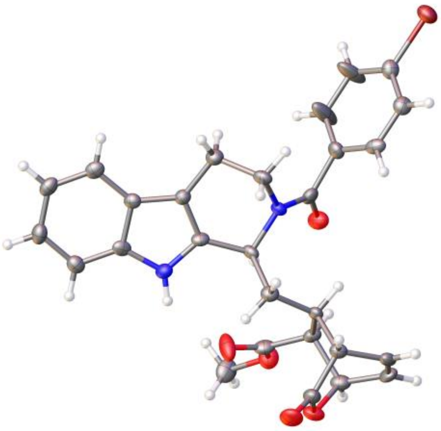

derivative of **11a**

11a was dissolved in DCM in a vial, and then n-hexane was added slowly to form a two-layer solution. And then wait for crystal precipitation while standing still. CCDC 2322046.

Supplementary Table 5. Crystal data and structure refinement for exp_8611

Identification code	exp_8611
Empirical formula	C28H25BrN2O5
Formula weight	549.41
Temperature / K	112(3)
Crystal system	monoclinic
Space group	P21/c
a / Å, b / Å, c / Å	10.1708(10), 9.6200(10), 24.436(4)
α/°, β/°, γ/°	90.00, 90.695(11), 90.00
Volume / Å3	2390.7(5)
Z	4
ρcalc / mg mm-3	1.526
μ / mm-1	1.763
F(000)	1128
Crystal size / mm3	0.34 × 0.33 × 0.13
2Θ range for data collection	5.82 to 52°
Index ranges	-12 ≤ h ≤ 10, -11 ≤ k ≤ 8, -29 ≤ l ≤ 29
Reflections collected	11892
Independent reflections	4621[R(int) = 0.0935 (inf-0.9Å)]
Data/restraints/parameters	4621/0/326
Goodness-of-fit on F2	1.081
Final R indexes [I>2σ (I) i.e. Fo>4σ (Fo)]	R1 = 0.0787, wR2 = 0.1508
Final R indexes [all data]	R1 = 0.1124, wR2 = 0.1696
Largest diff. peak/hole / e Å-3	0.946/-1.400
Flack Parameters	N
Completeness	0.9971

Supplementary Figure 10. Comparison of NMR Spectral of Alstovenine (**3**)

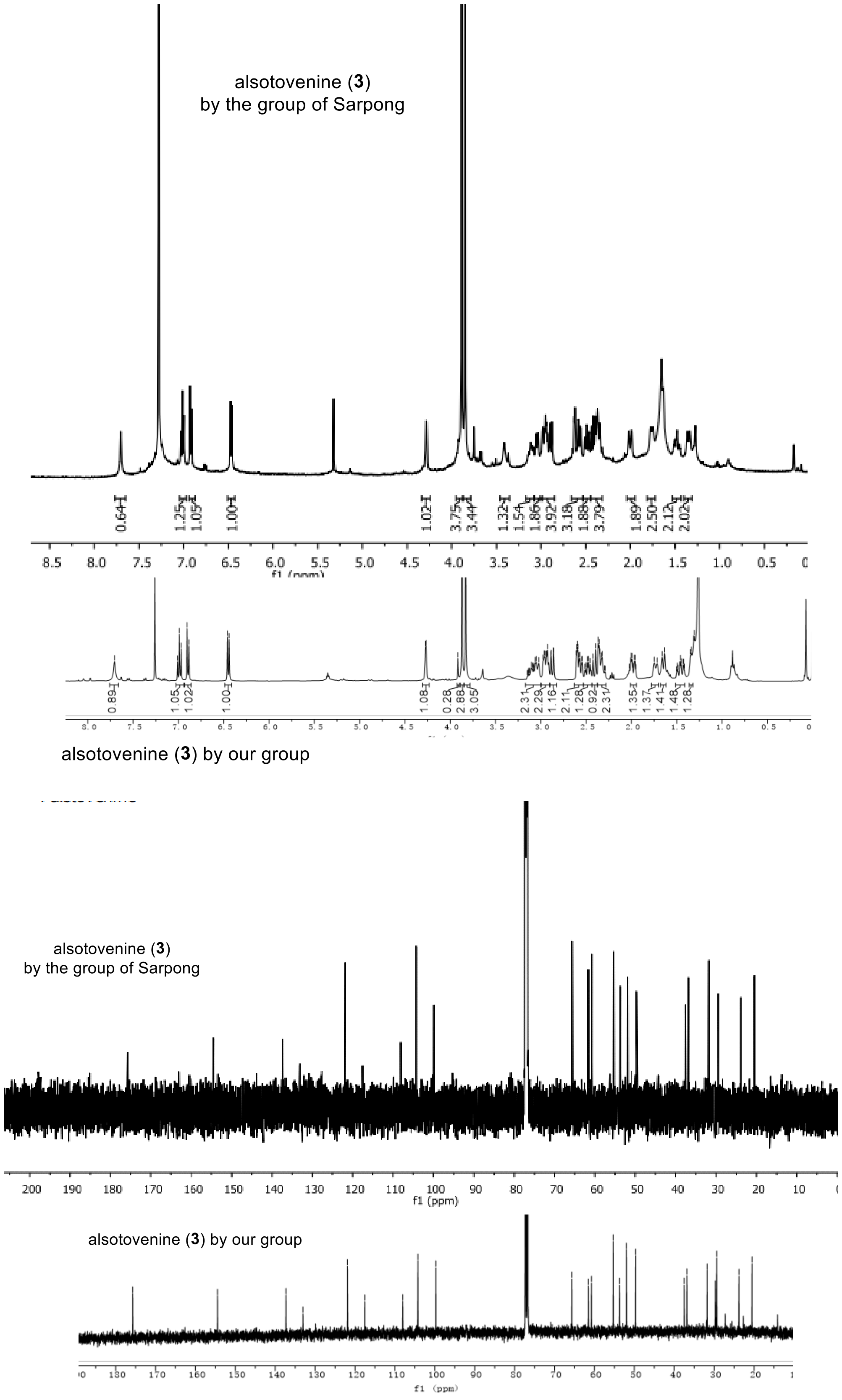

Supplementary Table 6. Comparison of ^{1}H NMR Spectral Data of Alstovenine (**3**)

Synthetic Alstovenine<br>by the group of Sarpong[2]<br>(600 MHz, $CDCl_3$) δH (*J* in Hz)	Synthetic Alstovenine<br>by our group<br>(400 MHz, $CDCl_3$) δH (*J* in Hz)
7.68 (s, 1H)	7.71 (s, 1H)
6.99 (t, J = 7.8 Hz, 1H)	6.99 (t, J = 7.9 Hz, 1H)
6.90 (d, J = 7.8 Hz, 1H)	6.89 (d, J = 8.1 Hz, 1H)
6.45 (d, J = 7.8Hz, 1H)	6.45 (d, J = 7.7 Hz, 1H)
4.27 (s, 1H)	4.27 (d, J = 2.8 Hz, 1H)
3.87 (s, 3H)	3.87 (s, 3H)
3.83 (s, 3H)	3.83 (s, 3H)
3.38 (s, 1H)	
3.13 – 3.06 (m, 1H)	3.16 – 3.06 (m, 1H)
3.03 (d, J = 10.8 Hz, 1H)	3.04 (d, J = 10.8 Hz, 1H)
2.97 – 2.91 (m, 2H)	3.00 – 2.90 (m, 2H)
2.87 (d, J = 11.4 Hz, 1H)	2.87 (d, J = 11.3 Hz, 1H)
2.62 – 2.59 (m, 1H)	2.63 – 2.57 (m, 1H)
2.55 (dd, J =11.4, 3.6 Hz, 1H)	2.56 (dd, J = 11.2, 3.3 Hz, 1H)
2.47 (dt, J =11.4, 4.2 Hz, 1H)	2.49 (dt, J = 11.7, 5.8 Hz, 1H)
2.42– 2.30 (m, 3H)	2.43 – 2.28 (m, 3H)
1.98 (dd, J = 14.4, 1.2 Hz, 1H)	1.98 (dd, J = 13.9, 3.3 Hz, 1H)
1.74 (d, J = 11.4 Hz, 1H)	1.73 (d, J = 13.0 Hz, 1H)
	1.65 (d, J = 11.1 Hz, 1H)
1.46 (t, J = 14.4 Hz, 1H)	1.46 (tt, J = 14.1, 3.9 Hz, 1H)
1.33 (d, J = 11.4 Hz, 1H)	1.33 d, J = 10.8 Hz, 1H).

Supplementary Table 7. Comparison of ^{13}C NMR Spectral Data of Alstovenine (**3**)

Synthetic Alstovenine<br>by the group of Sarpong[2]<br>(150MHz, $CDCl_3$)	Synthetic Alstovenine<br>by our group<br>(100 MHz, $CDCl_3$)
175.7	175.7
154.5	154.5
137.3	137.3
133.1	133.1
121.9	121.9
117.6	117.6
108.1	108.1
104.2	104.3
99.8	99.8
65.7	65.7
61.6	61.5
60.8	60.8
55.3	55.3
53.7	53.7
51.9	52.0
49.7	49.7
37.6	37.5
36.8	36.8
31.8	31.8
29.4	29.4
23.8	23.8
20.5	20.5

Supplementary Figure 11. Comparison of NMR Spectral of venenatine (**4**)

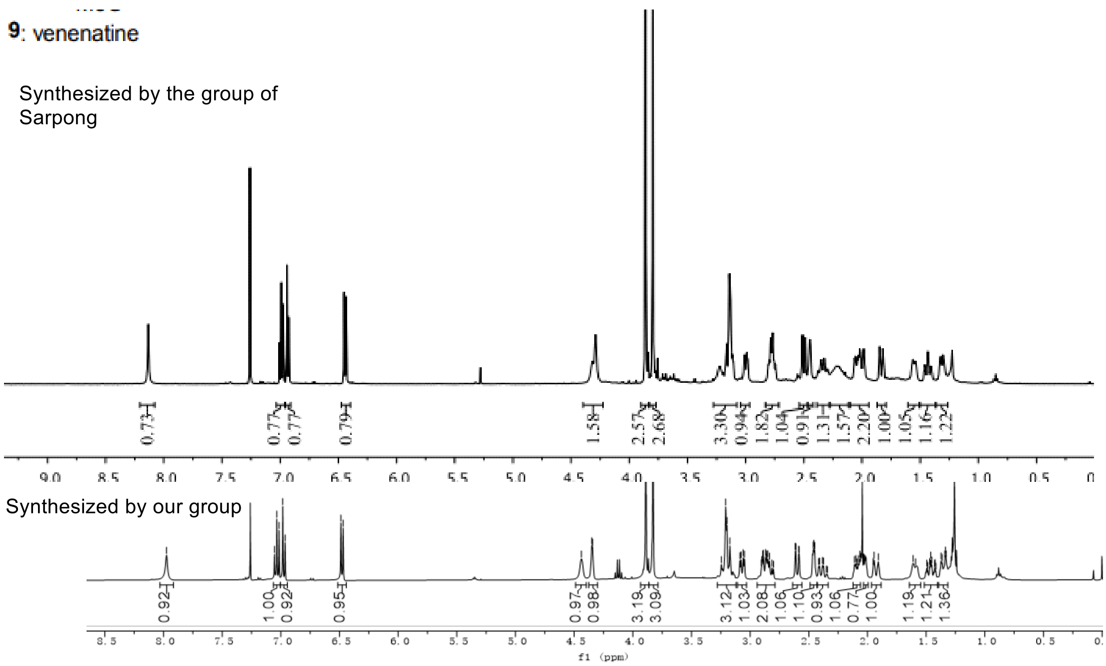

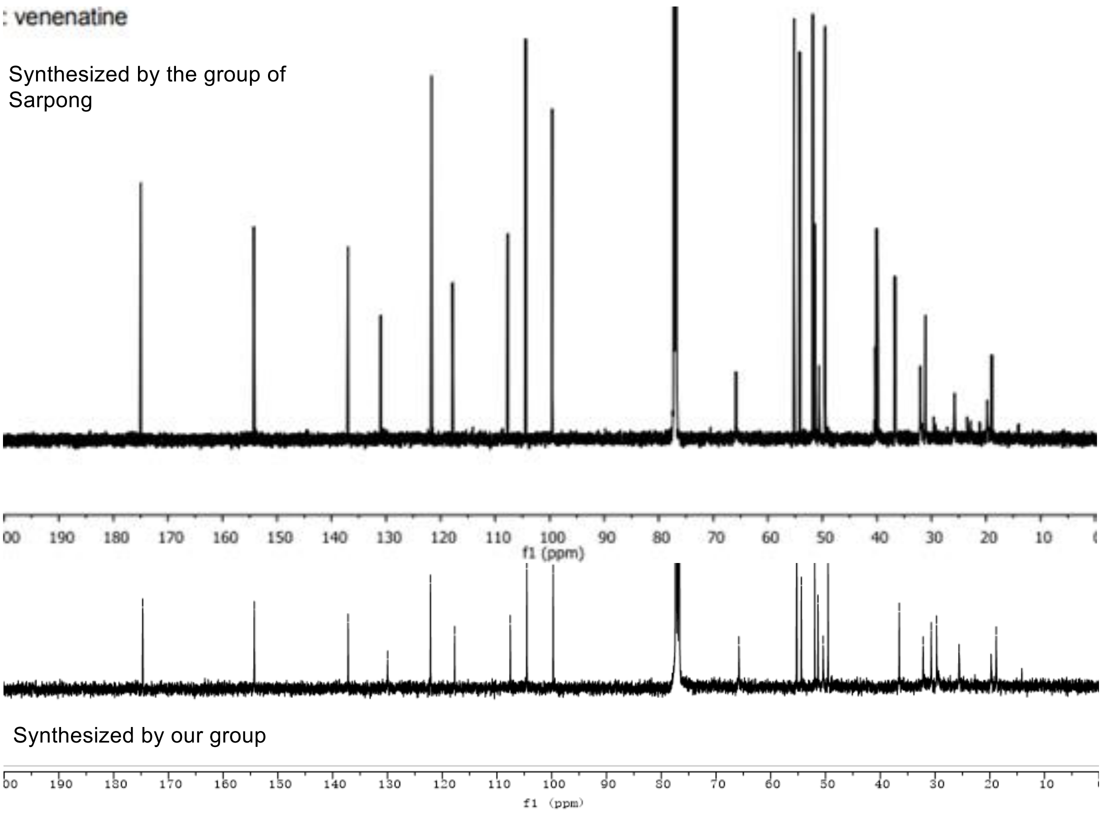

Supplementary Table 8. Comparison of ^{1}H NMR Spectral Data of venenatine (**4**)

Synthetic venenatine<br>by the group of Sarpong[2]<br>(500 MHz, $CDCl_3$) δH (*J* in Hz)	Synthetic venenatine<br>by our group<br>(400 MHz, $CDCl_3$) δH (*J* in Hz)
8.13 (s, 1H)	7.97 (s, 1H)
6.99 (dd, J =7.5, 8.0 Hz, 1H)	7.03 (t, J = 7.9 Hz, 1H)
9.94 (d, J = 8.0 Hz, 1H)	6.97 (d, J = 8.1 Hz, 1H)
6.44 (d, J = 7.5 Hz, 1H)	6.48 (d, J = 7.6 Hz, 1H)
4.32 (s, 1H)	4.44 (s, 1H)
4.29 (s, 1H)	4.35 (d, J = 2.9 Hz, 1H)
3.86 (s, 3H)	3.89 (s, 3H)
3.80 (s, 3H)	3.83 (s, 3H)
3.25 – 3.10 (m, 3H)	3.28 – 3.12 (m, 3H)
3.00 (dd, J = 11.5, 3.5 Hz, 1H)	3.07 (dd, J = 11.6, 4.2 Hz, 1H)
2.82-2.73 (m,2H)	2.94 – 2.79 (m, 2H)
2.50 (dd, J = 11.5, 2.0 Hz, 1H)	2.60 (dd, J = 11.6, 2.1 Hz, 1H)
2.45 (br.s, 1H)	2.49 – 2.43 (m, 1H)
2.34 (dq, J = 12.5, 3.0 Hz, 1H)	2.38 (td, J = 13.2, 3.6 Hz, 1H)
2.21 (br.s, 1H)	2.12 – 2.06 (m, 1H)
2.08-1.98 (m, 2H)	2.03 – 1.99 (m, 1H),
1.83 (d, J = 14.0 Hz,1H)	1.97 – 1.88 (m, 1H)
1.55 (d, J =12.5 Hz, 1H)	1.60 (d, J = 8.4 Hz, 1H)
1.43 (tt, J = 13.5, 3.5 Hz, 1H)	1.52 – 1.40 (m, 1H)
1.32 (dd, J = 13.5, 3.5 Hz, 1H)	1.39 – 1.31 (m, 1H)

Supplementary Table 9. Comparison of ^{13}C NMR Spectral Data of venenatine (**4**)

Synthetic venenatine<br>by the group of Sarpong[2]<br>(150MHz, $CDCl_3$)	Synthetic venenatine<br>by our group<br>(100 MHz, $CDCl_3$)
175.0	174.7
154.2	154.3
137.0	137.2
131.0	130.0
121.6	122.1
117.8	117.7
107.7	107.6
104.4	104.6
99.5	99.7
65.8	65.8
55.2	55.3
54.2	54.4
51.8	51.9
51.4	51.4
50.6	50.4
49.5	49.5
36.7	36.5
32.0	32.2
31.1	30.7
25.8	25.6
19.8	19.8
18.9	18.8

Supplementary Figure 12: Comparison of NMR Spectral of β-yohimbine (**1b**)

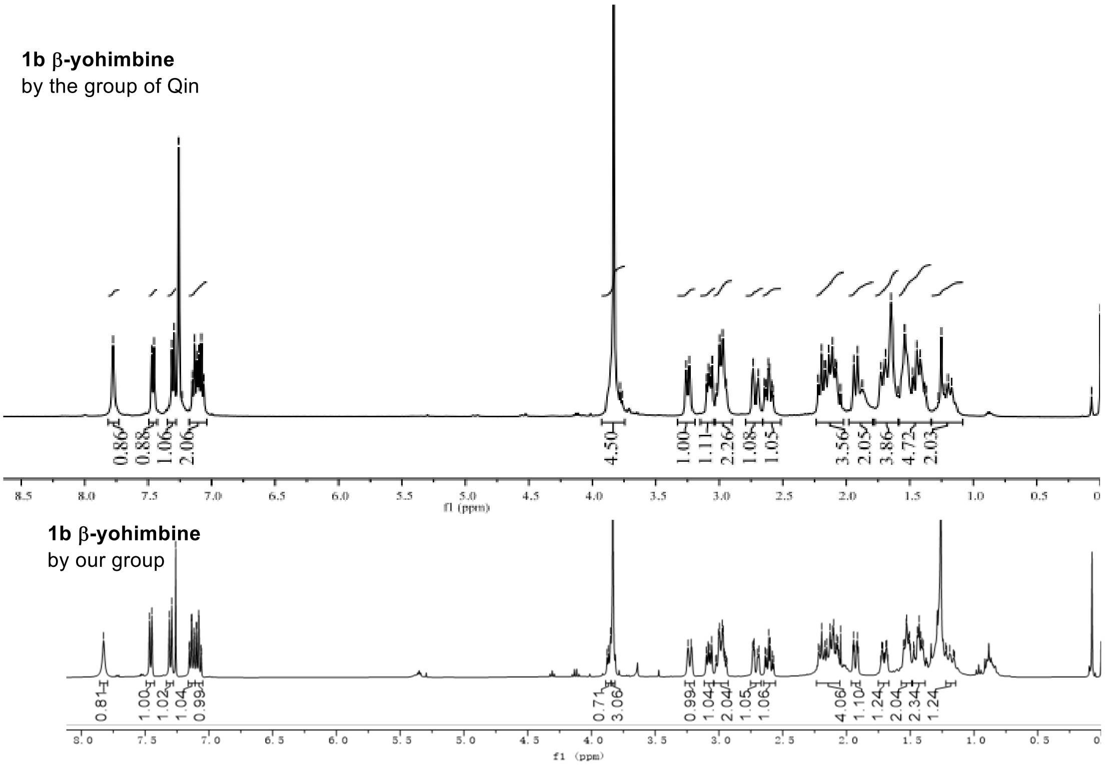

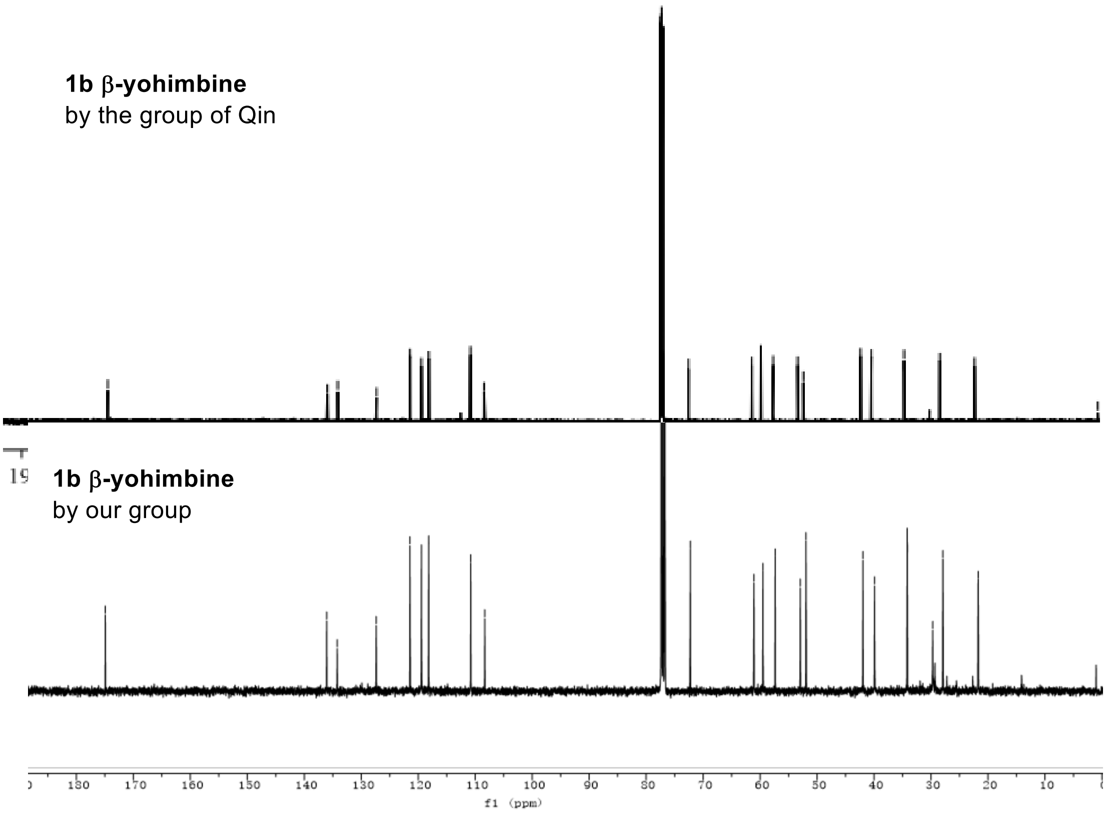

Supplementary Table 10. Comparison of 1H NMR Spectral Data of β-yohimbine (**1b**)

Synthetic β-yohimbine by the group of Qin[3] (400 MHz, CDCl3) δH (J in Hz)	Synthetic β-yohimbine by our group (400 MHz, CDCl3) δH (J in Hz)
7.78 (brs, 1H)	7.83 (s, 1H)
7.46 (d, J = 7.6 Hz, 1H)	7.46 (d, J = 7.7 Hz, 1H)
7.30 (d, J = 8.0 Hz, 1H)	7.30 (d, J = 7.9 Hz, 1H)
7.15 – 7.06 (m, 2H)	7.15 – 7.06 (m, 2H)
3.88 – 3.77 (m, 1H)	3.89 – 3.76 (m, 1H)
3.83 (s, 3H)	3.83 (s, 3H)
3.25 (d, J = 10.8 Hz, 1H)	3.23 (d, J = 9.6 Hz, 1H)
3.10 – 2.95 (m, 3H)	3.10 – 2.95 (m, 3H)
2.73-2.57 (m, 2H)	2.75-2.67 (m, 1H)<br>2.60 (td, J=11.1, 4.4 Hz, 1H)
2.24-2.05 (m, 3H)	2.24-2.05(m, 4H)
1.94-1.87(m, 2H)	1.93 (dt, J=12.1, 2.5 Hz, 1H)
1.72-1.64 (m, 1H)	1.70 (m, 1H)
1.53-1.37 (m, 4H)	1.53 (m, 2H)<br>1.48-1.38 (m, 2H)
1.23-1.14 (m, 1H)	1.22-1.14 (m, 1H)

Supplementary Table 11. Comparison of 13C NMR Spectral Data of β-yohimbine (**1b**)

Synthetic β-yohimbine by the group of Qin[3] (150MHz, CDCl3)	Synthetic β-yohimbine by our group (100 MHz, CDCl3)
174.8,	174.9
136.0	136.0
134.2	134.2
127.3	127.4
121.4	121.4
119.4	119.4
118.1	118.1
110.8	110.8
108.3	108.3
72.2	72.2
61.1	61.1
59.5	59.5
57.3	57.3
53.0	52.0
52.0	51.9
41.9	42.0
40.0	39.9
34.2	34.2
34.2	34.2
27.9	27.9
21.7	21.7

Supplementary Table 12. NMR comparation of pseudoyohimbine (**2**)

pseudoyohimbine<br>Synthesied by Brown[4]	1H NMR<br>(400 MHz, $CDCl_3$)
	8.52 (s, 1H)
	7.48 (d, J = 7.7 Hz, 1H)
	7.44 (d, J = 8.0 Hz, 1H)
	7.23 – 7.15 (m, 2H)
	7.18 – 7.09 (m, 1H)
4.75 (ddd, J=5, 2.5, 1.5 Hz, 1H)	4.74 (d, J = 4.4 Hz, 1H)
4.29 (bs, J=3, 2.5, 2 Hz, 1H)	4.23 (d, J = 2.9 Hz, 1H)
3.92 (s, 3H)	3.78 (s, 3H)
3.44 (m, 2H)	3.47 – 3.37 (m, 2H)
3.07 (dm, J=16 Hz, 1H)	3.03 (m, 1H),
2.8 (dm, 2H)	2.89 – 2.77 (m, 2H)
2.68 (t, J=12 Hz, 1Hx)	2.68 (t, J = 11.3 Hz, 1H)
2.34 (dd, J=12, 3 Hz, H6)	2.39 – 2.31 (m, 1H)
2.30 (dm, 1H)	2.26 (dd, J = 11.4, 2.6 Hz, 1H)
2.00 (td, J=12, 5 Hz, 1H)	2.05 – 1.94 (m, 1H)
1.87 (dm, J=12, 3Hz, 1H-)	1.86 – 1.73 (m, 2H)
1.78-1.55 (tm, J=12 Hz, 1H; tm, J=12 Hz,	1.68 (td, J = 11.6, 2.6 Hz, 1H)
1H; td, J=12, 3.5 Hz, 1H),	1.51 (tdd, J = 13.8, 4.4, 2.4 Hz, 1H)
1.30 (bq, J=12 Hz, 1 H)	1.33 – 1.28 (m, 2H)
	1.24 – 1.15 (m, 2H)
0.92 (dbd, J=12, 3 Hz, 1H)	0.96 – 0.80 (m, 1H)

Supplementary Figure 13. ^{1}H NMR of 10 (400 MHz, $CDCl_3$)

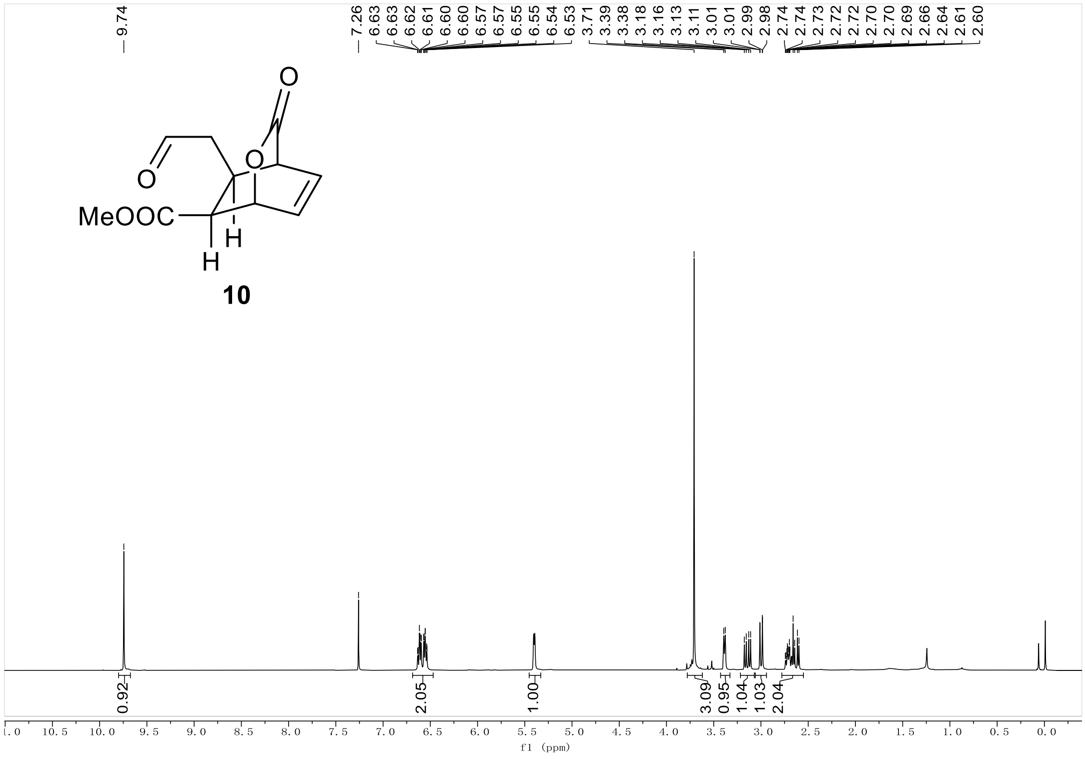

Supplementary Figure 14. ^{13}C NMR of 10 (100 MHz, $CDCl_3$)

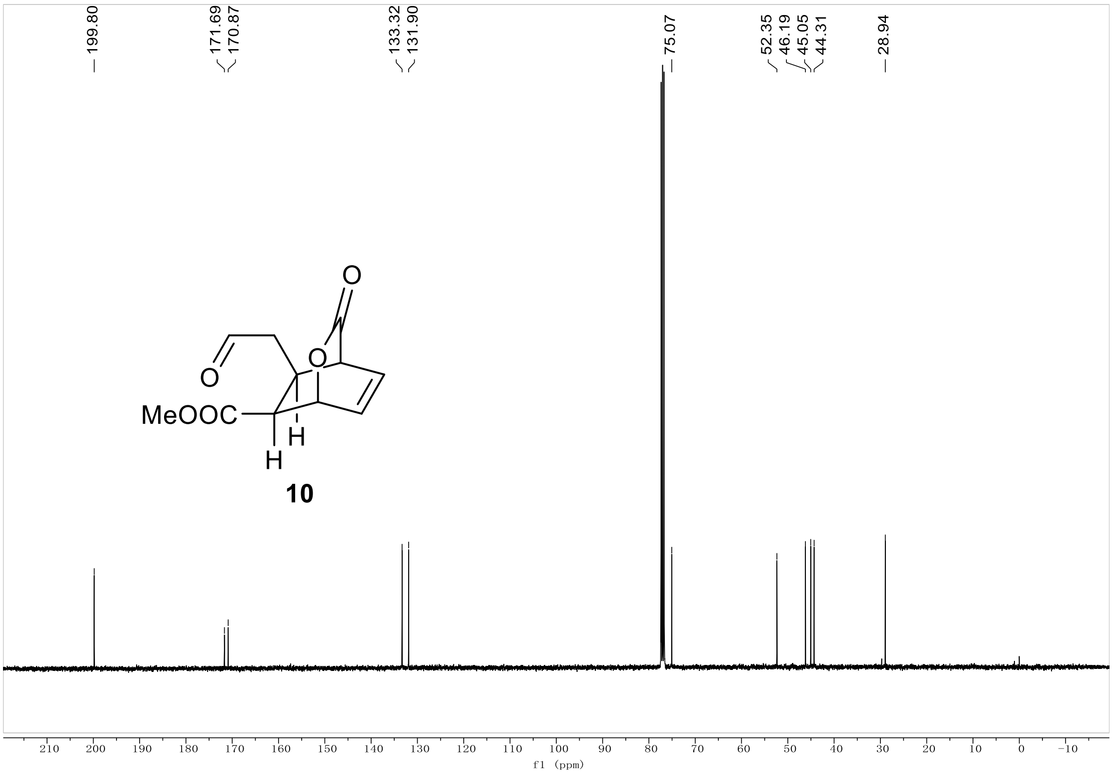

Supplementary Figure 15. ^{1}H NMR of 8a (400 MHz, DMSO-d6)

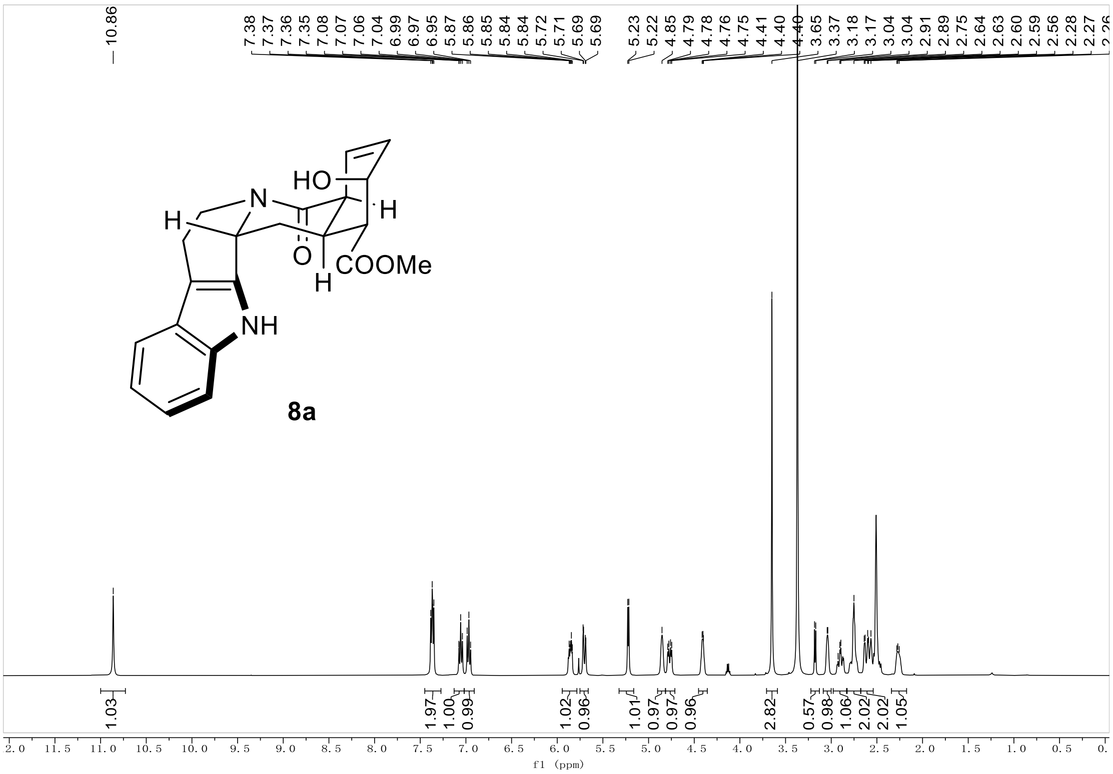

Supplementary Figure 16. ^{13}C NMR of 8a (100 MHz, DMSO-d6)

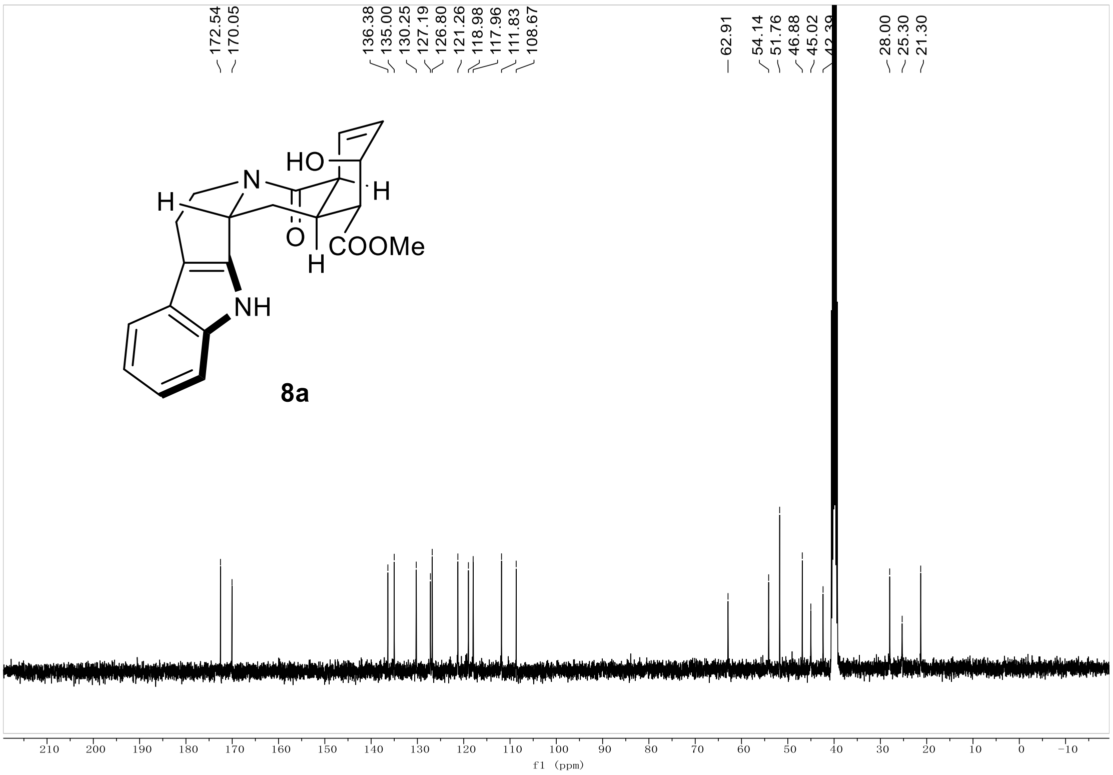

Supplementary Figure 17. ^{1}H NMR of 11a (400 MHz, $CDCl_3$)

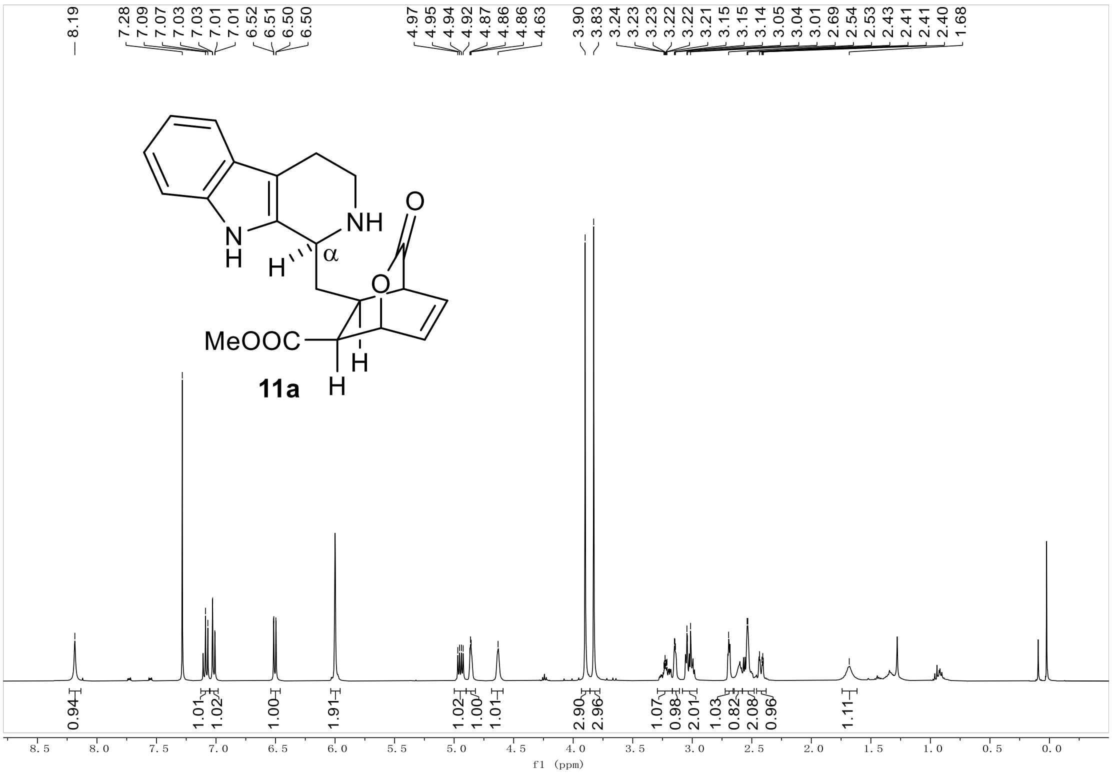

Supplementary Figure 18. ^{13}C NMR of 11a (100 MHz, $CDCl_3$)

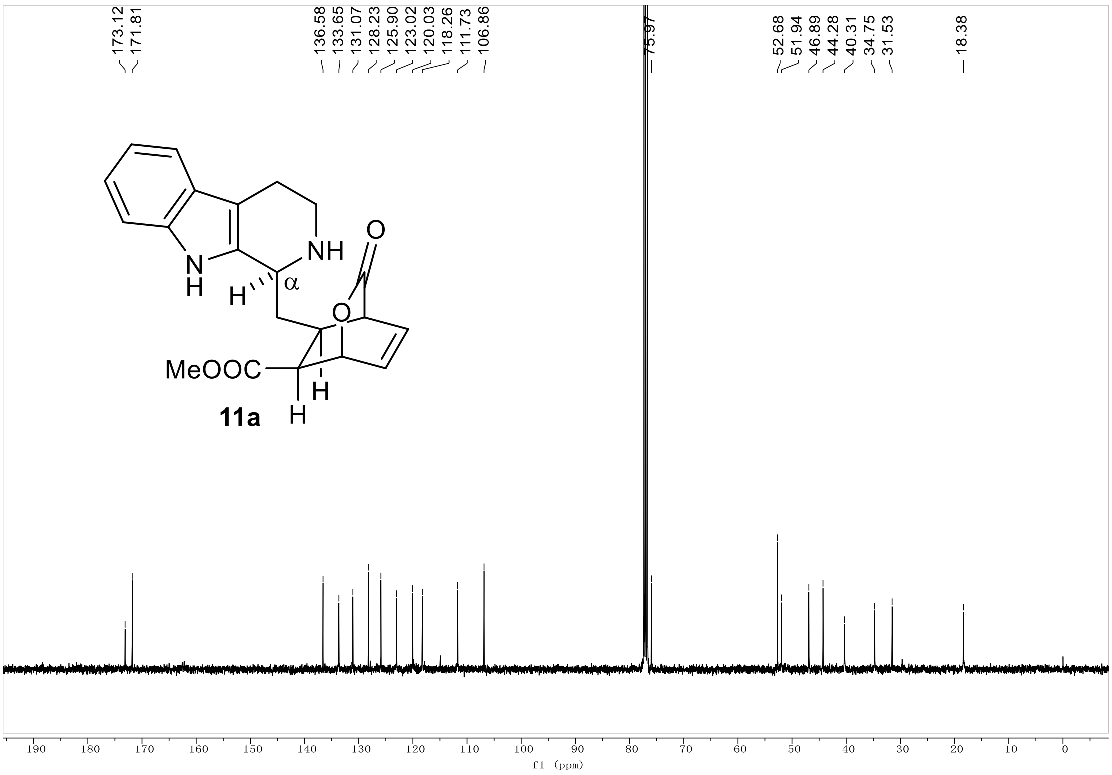

Supplementary Figure 19. ^{1}H NMR of 11c (400 MHz, $CDCl_3$)

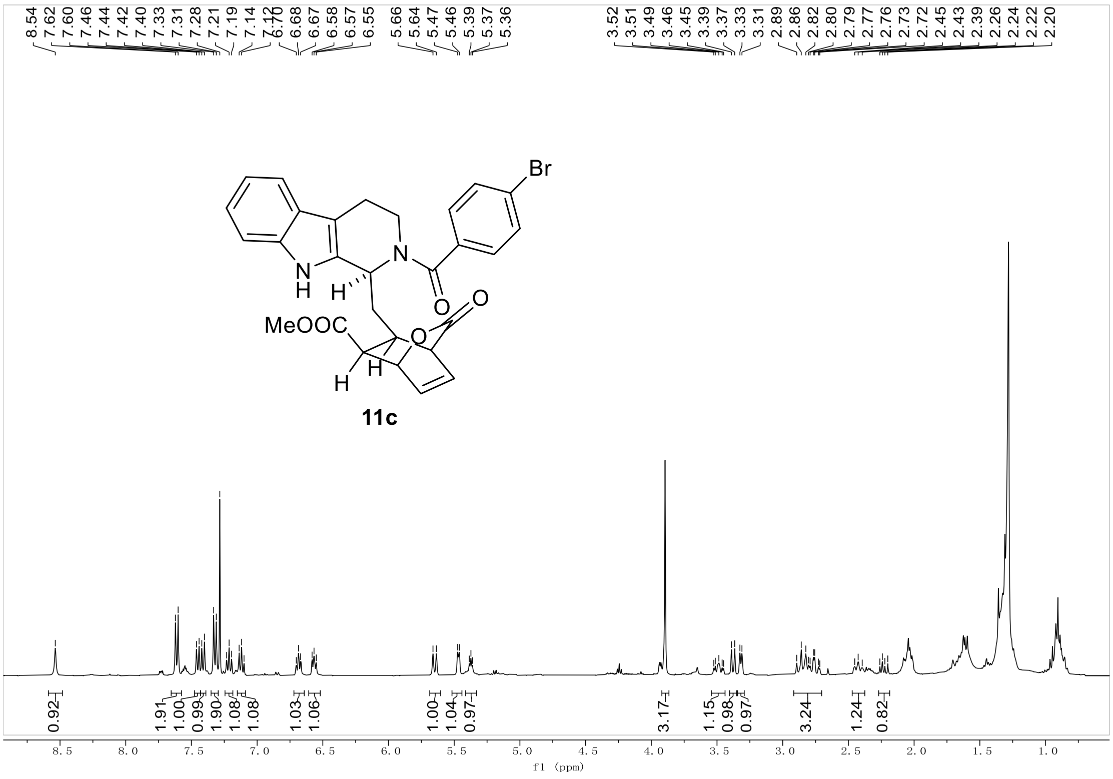

Supplementary Figure 20. ^{13}C NMR of 11c (100 MHz, $CDCl_3$)

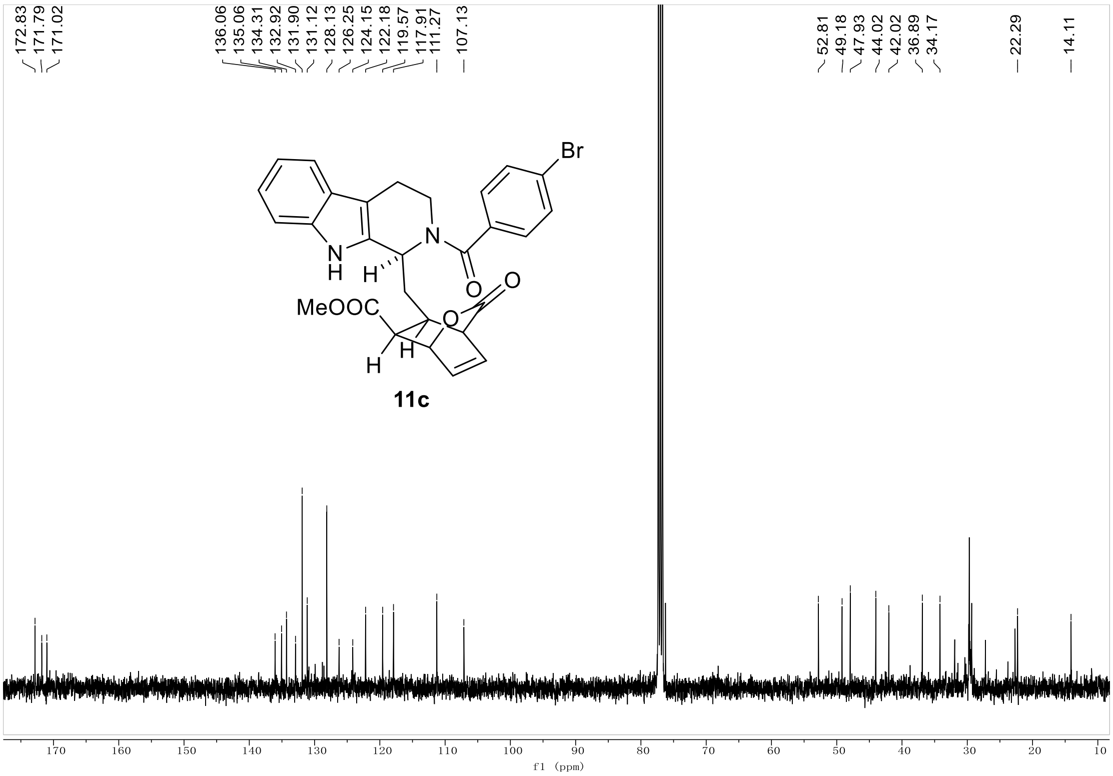

Supplementary Figure 21. ^{1}H NMR of 8b (400 MHz, $CDCl_3$)

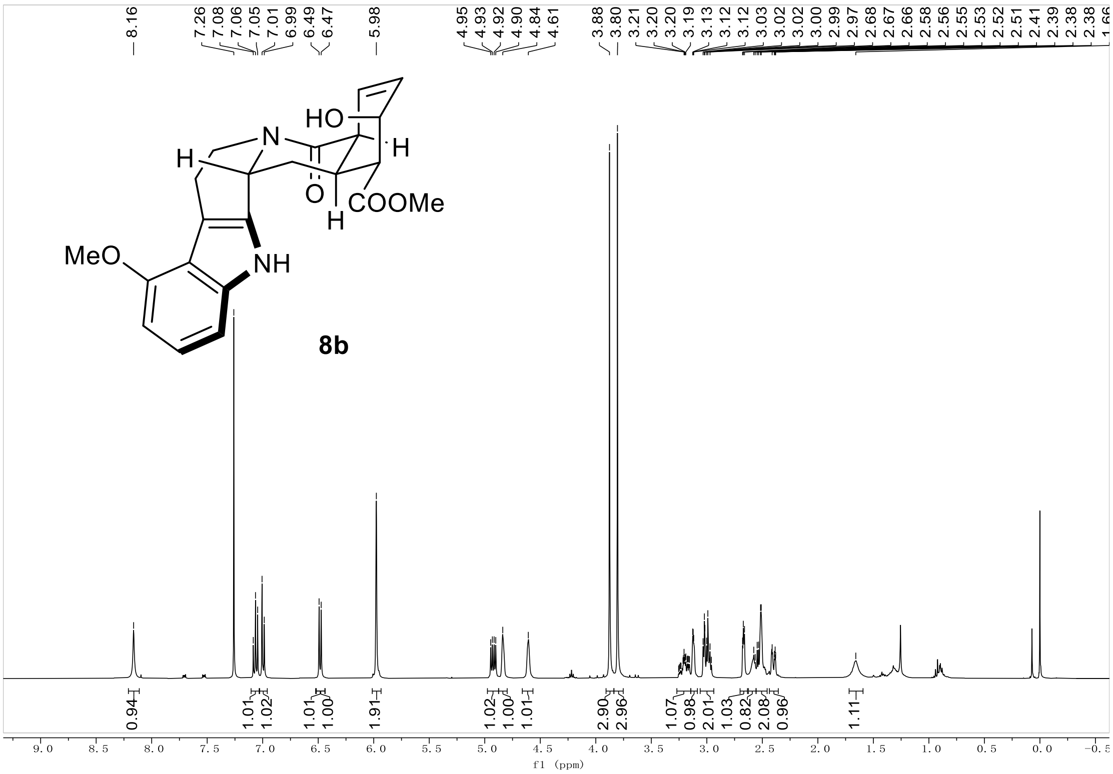

Supplementary Figure 22. ^{13}C NMR of 8b (100 MHz, $CDCl_3$)

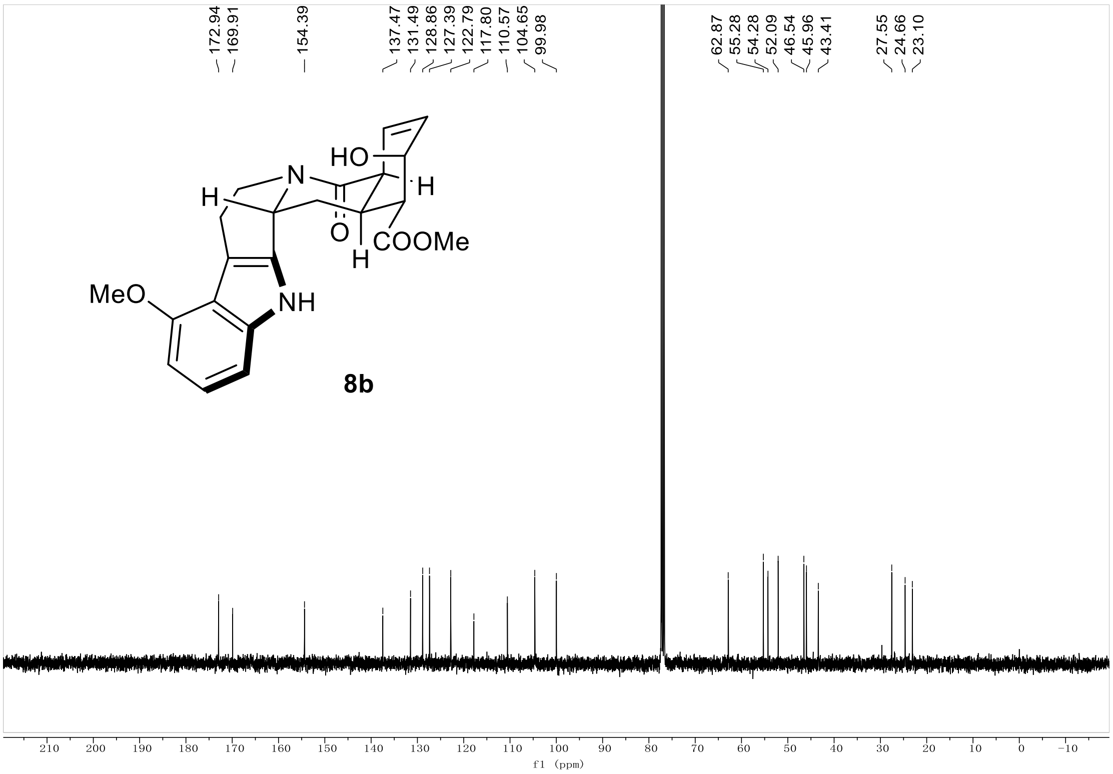

Supplementary Figure 23. ^{1}H NMR of 12 (400 MHz, DMSO-d6)

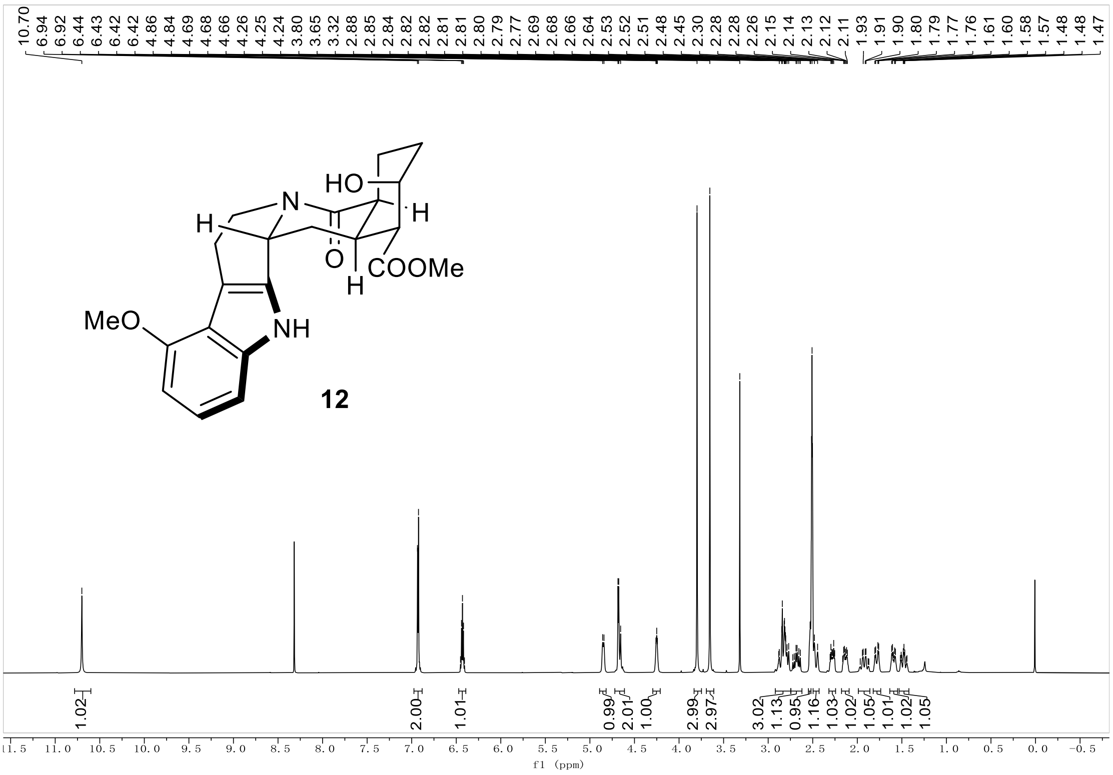

Supplementary Figure 24. ^{13}C NMR of 12 (100 MHz, DMSO-d6)

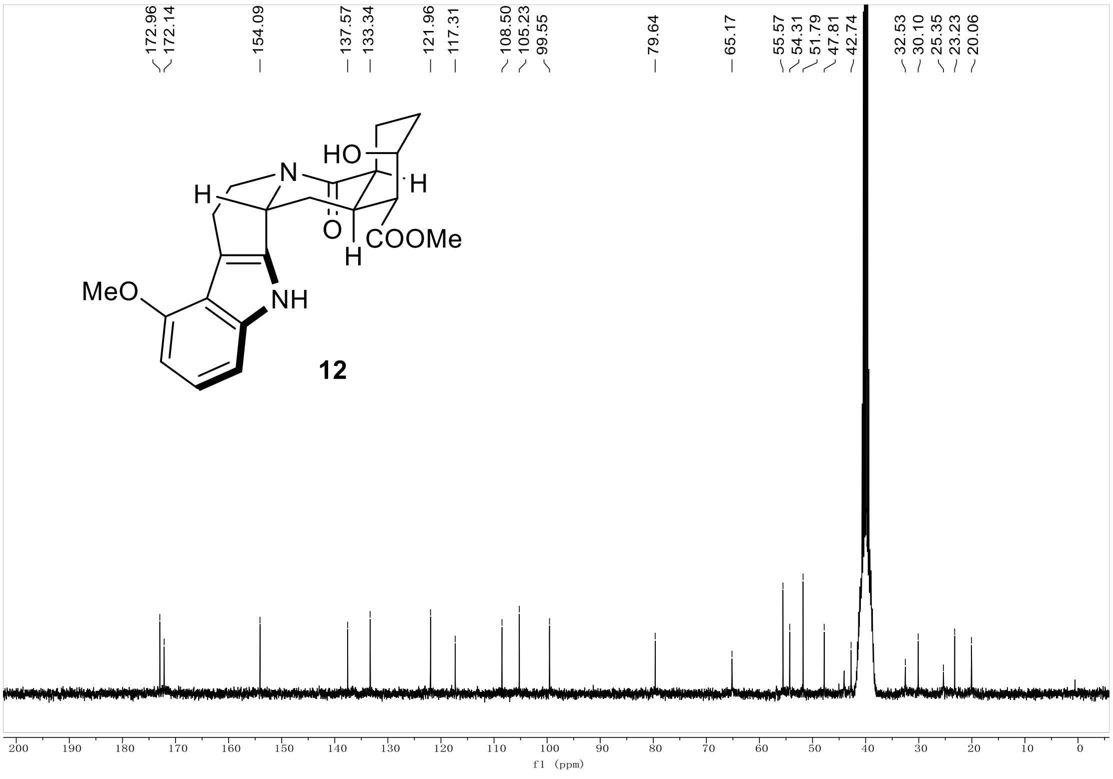

Supplementary Figure 25. ^{1}H NMR of 4 (400 MHz, $CDCl_3$)

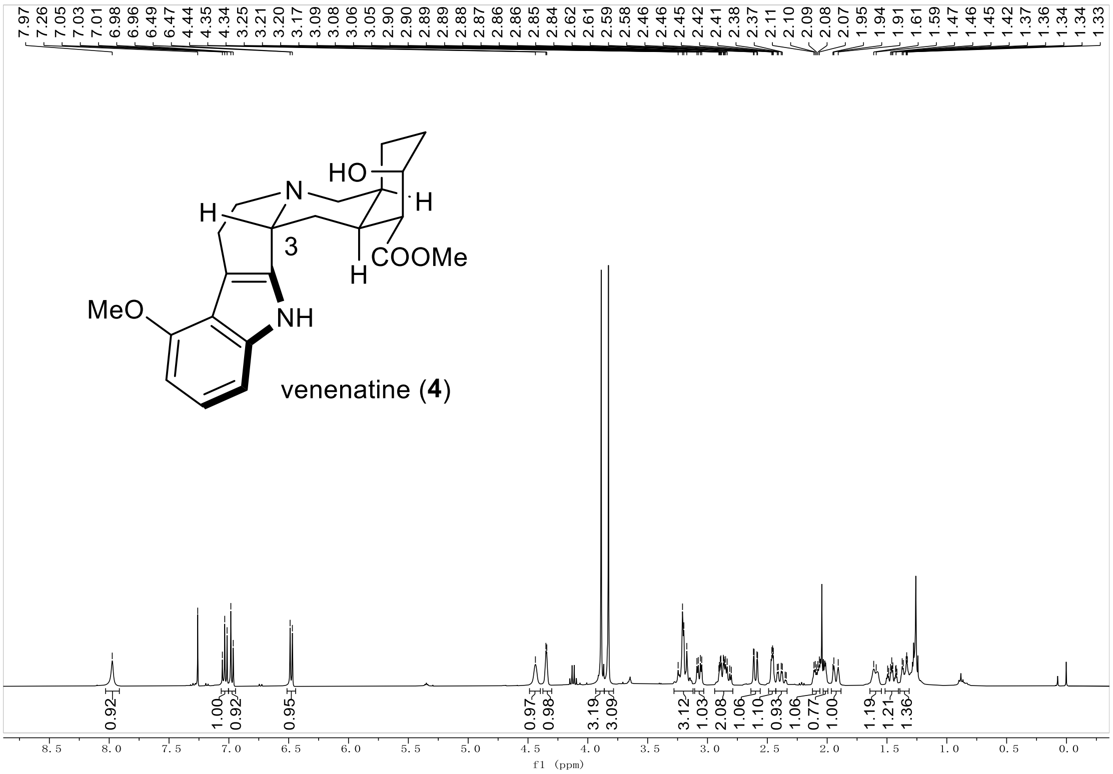

Supplementary Figure 26. ^{13}C NMR of 4 (100 MHz, $CDCl_3$)

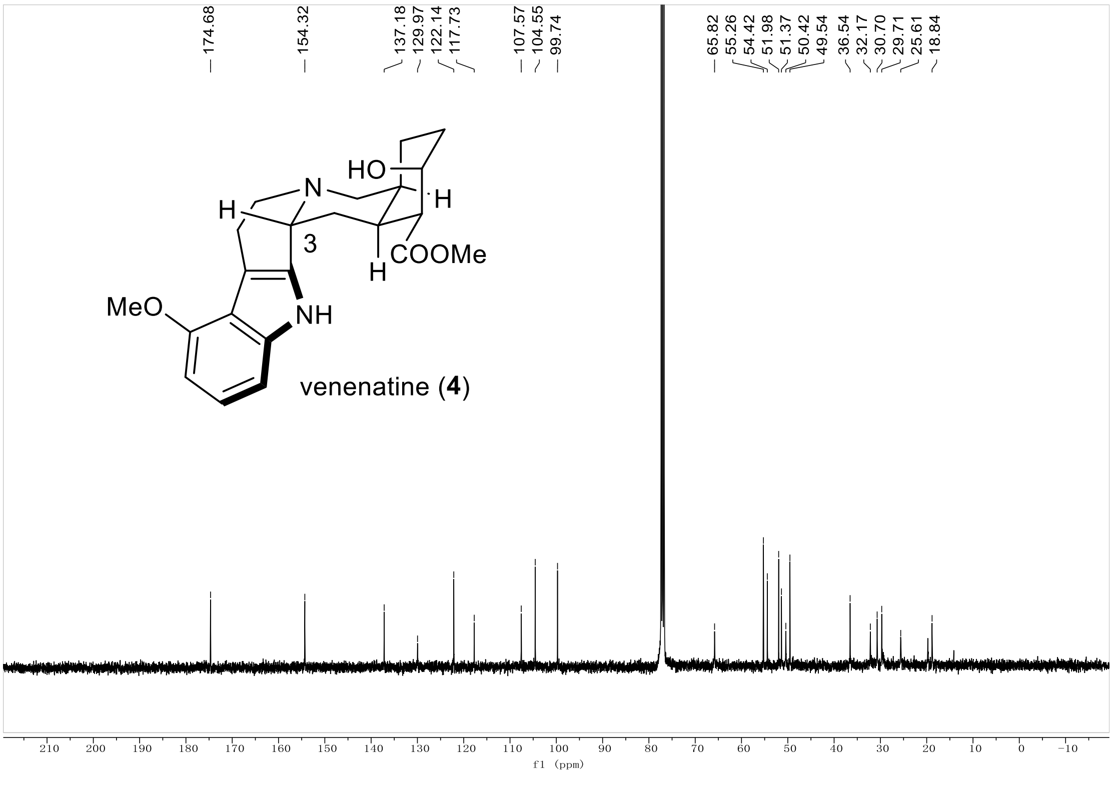

Supplementary Figure 27. ^{1}H NMR of 13 (400 MHz, DMSO-d6)

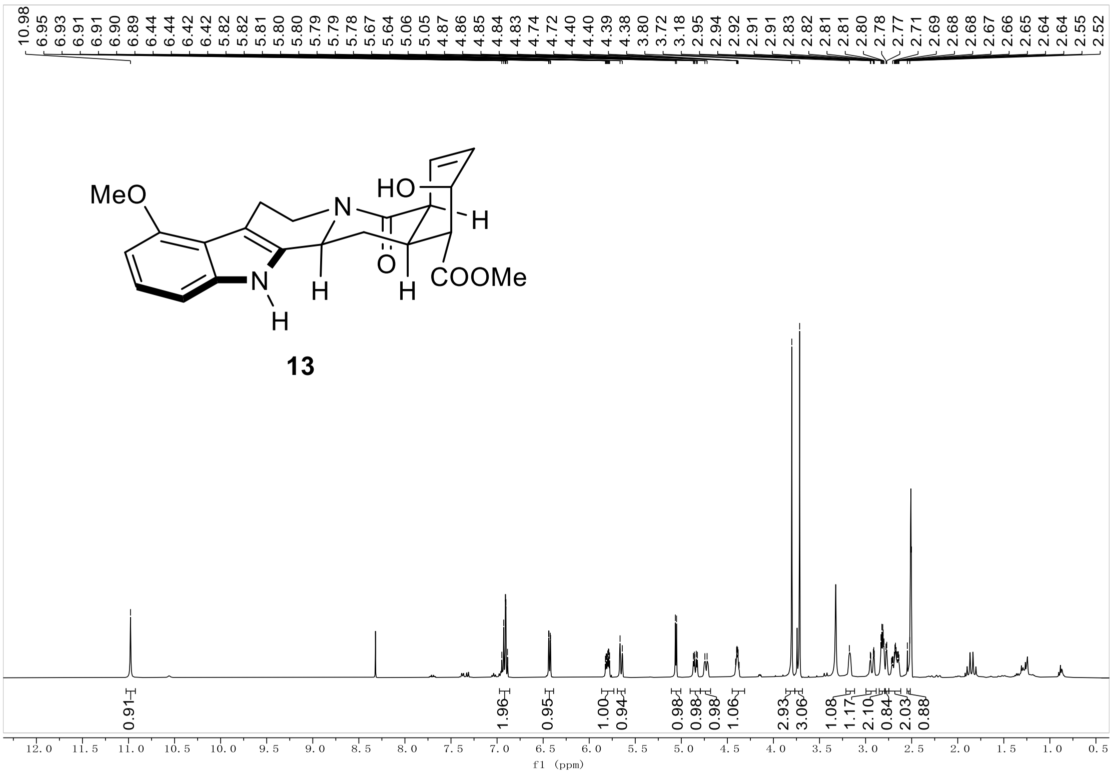

Supplementary Figure 28. ^{13}C NMR of 13 (100 MHz, DMSO-d6)

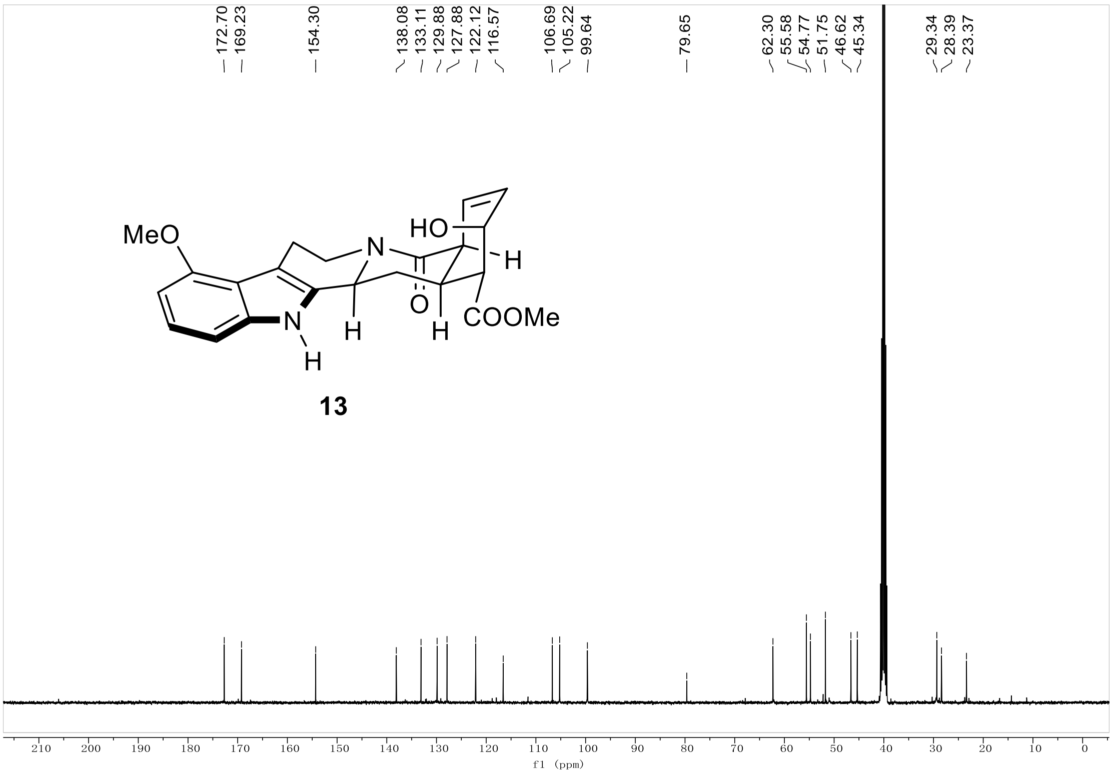

Supplementary Figure 29. ^{1}H NMR of 14 (400 MHz, DMSO-d6)

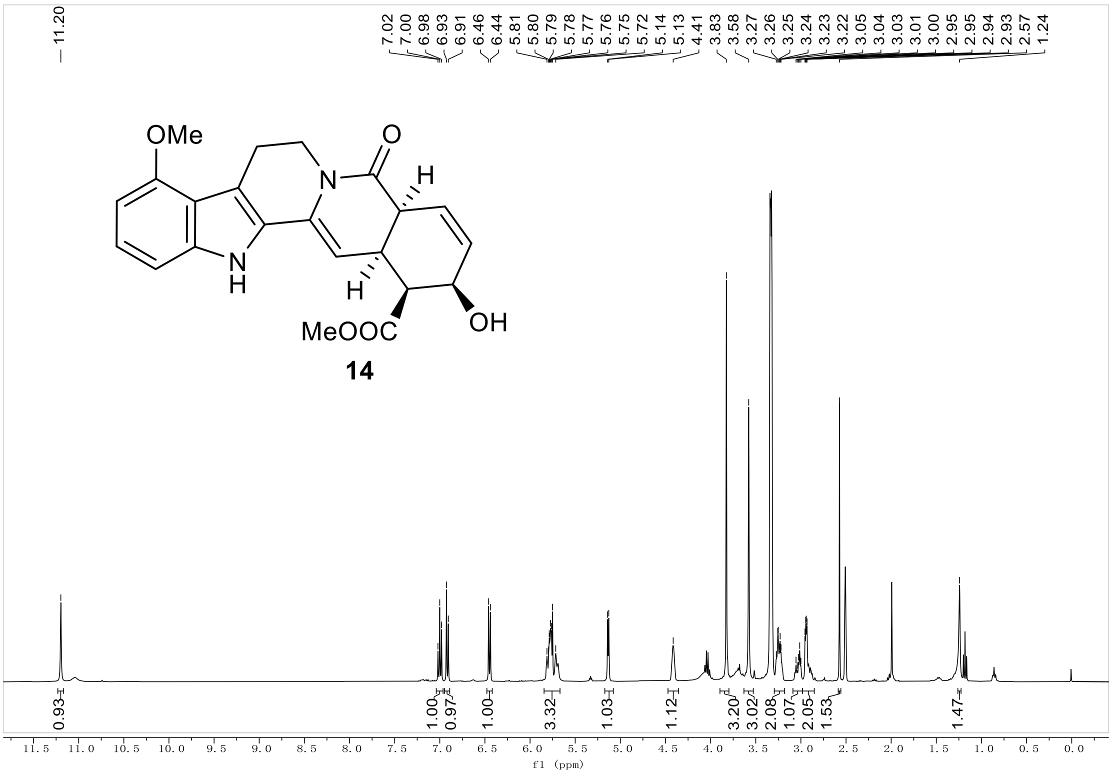

Supplementary Figure 30. ^{13}C NMR of 14 (100 MHz, DMSO-d6)

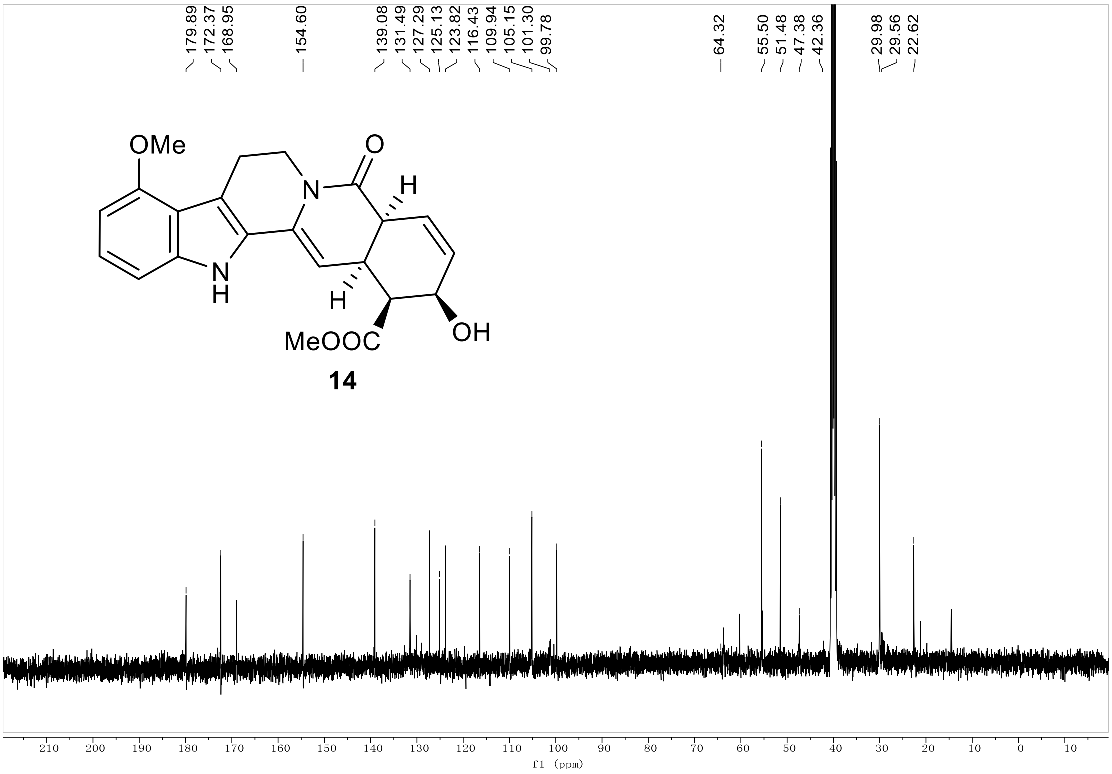

Supplementary Figure 31. HSQC NMR of 14 (100 MHz, DMSO-d6)

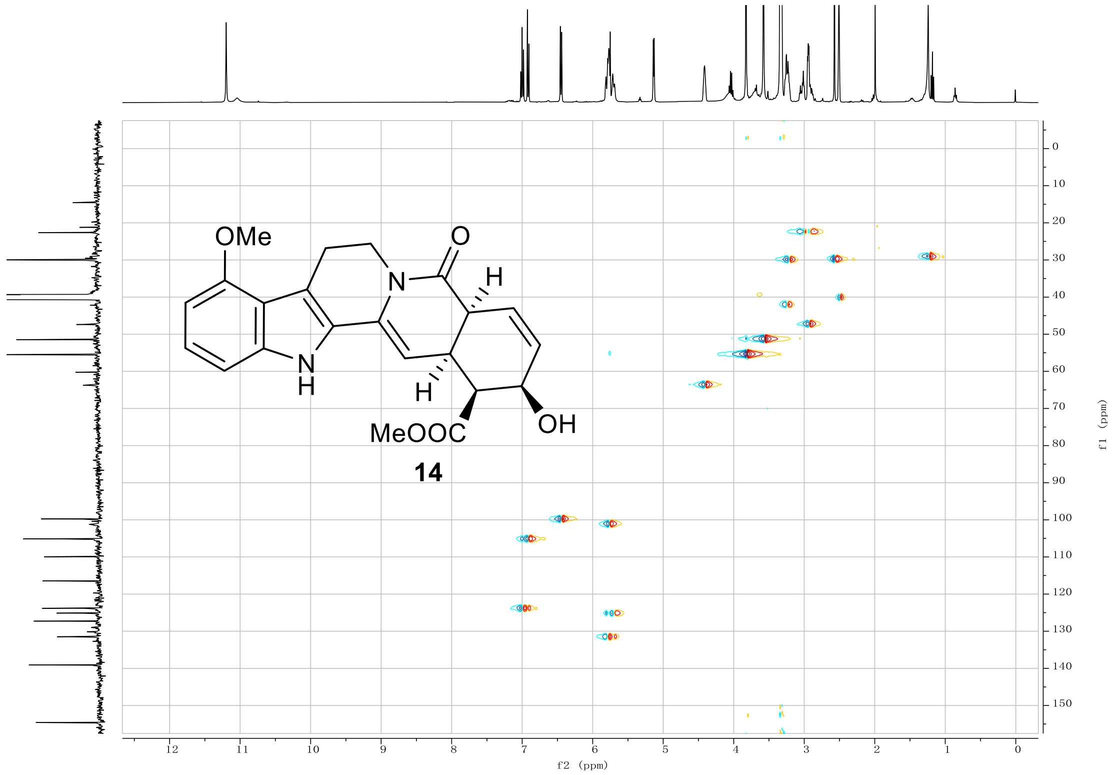

Supplementary Figure 32. ^{1}H NMR of 13’ (400 MHz, $CDCl_3$)

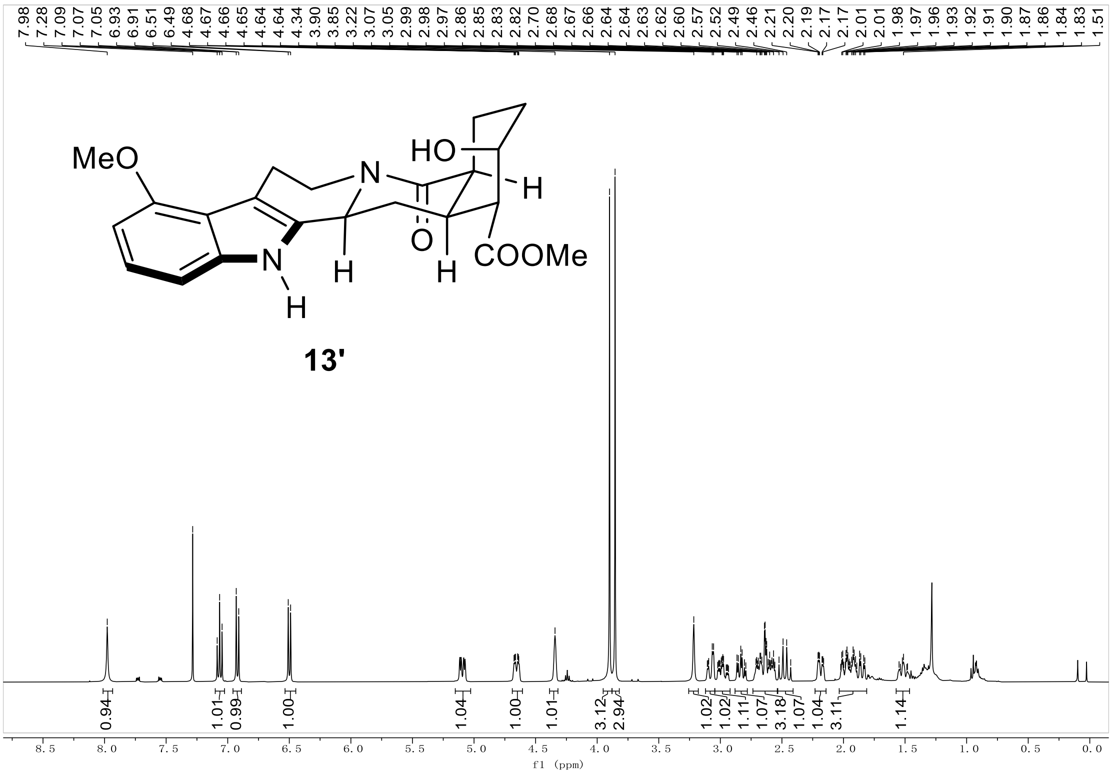

Supplementary Figure 33. ^{13}C NMR of 13’ (100 MHz, $CDCl_3$)

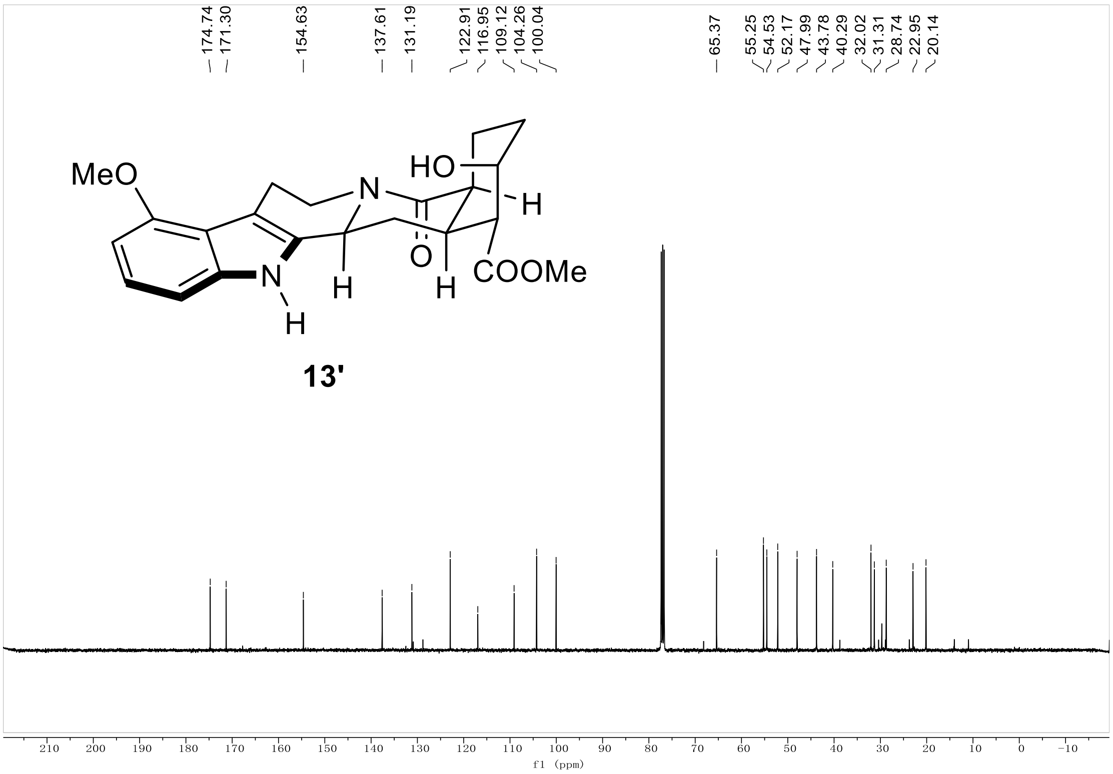

Supplementary Figure 34. ^{1}H NMR of 3 (400 MHz, $CDCl_3$)

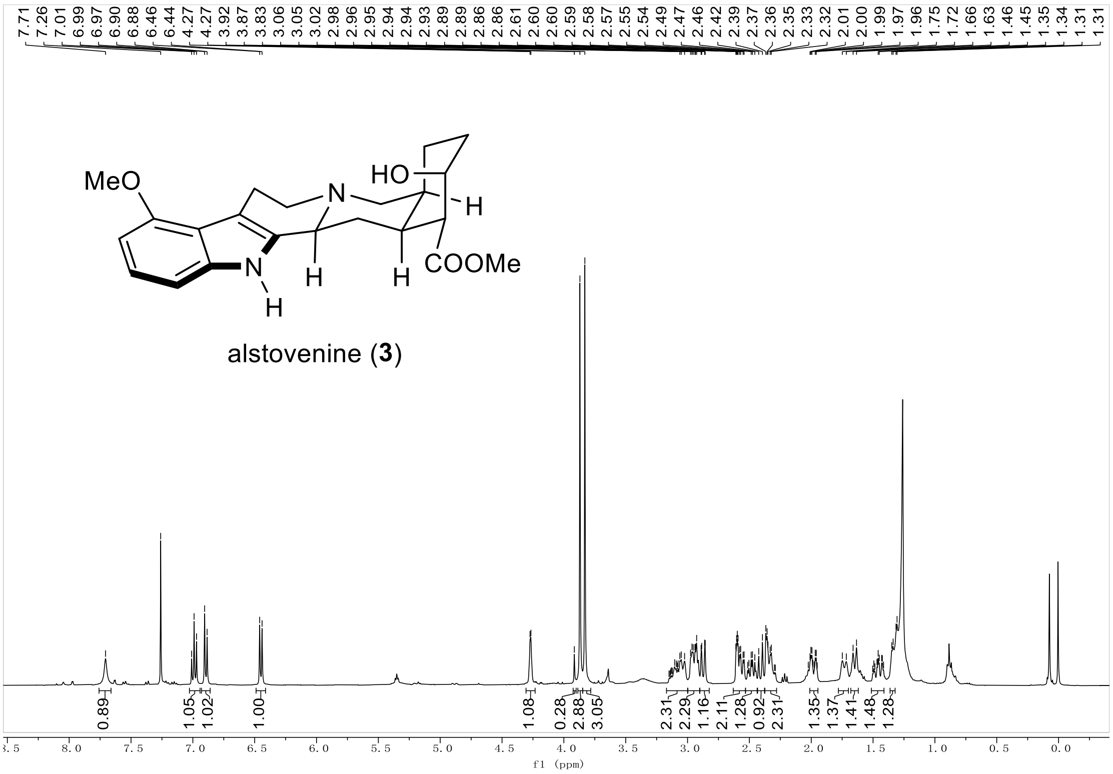

Supplementary Figure 35. ^{13}C NMR of 3 (100 MHz, $CDCl_3$)

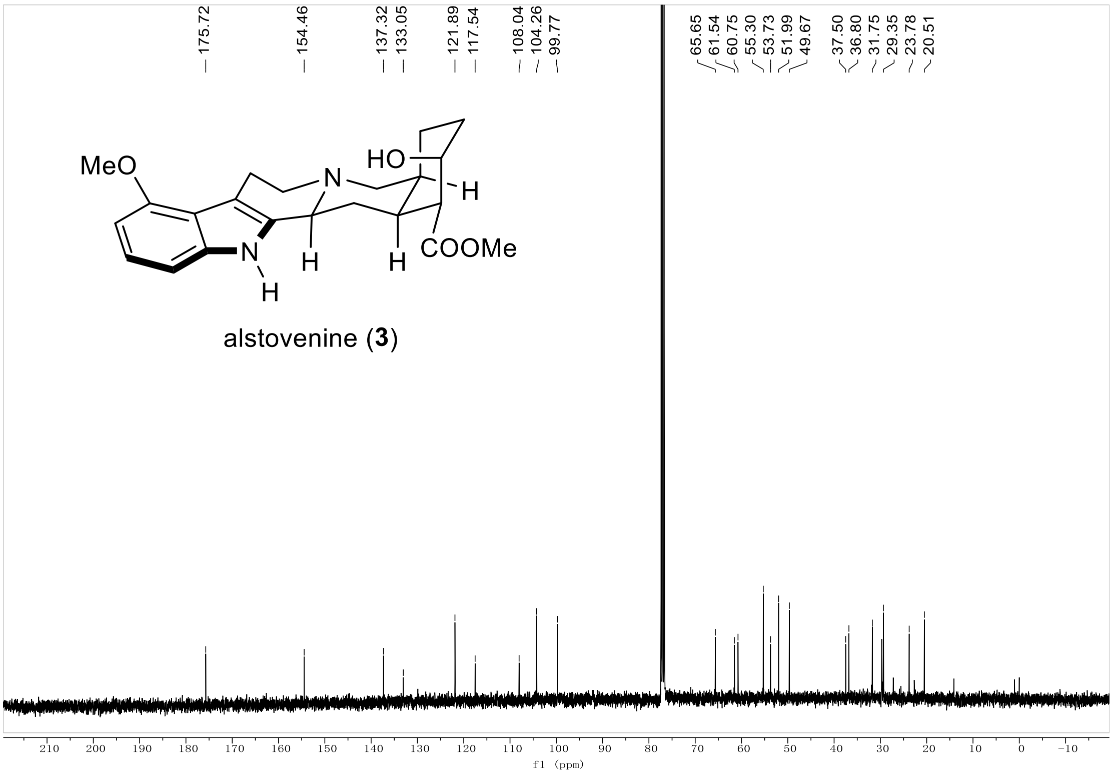

Supplementary Figure 36. ^{1}H NMR of 17 (400 MHz, DMSO-d6)

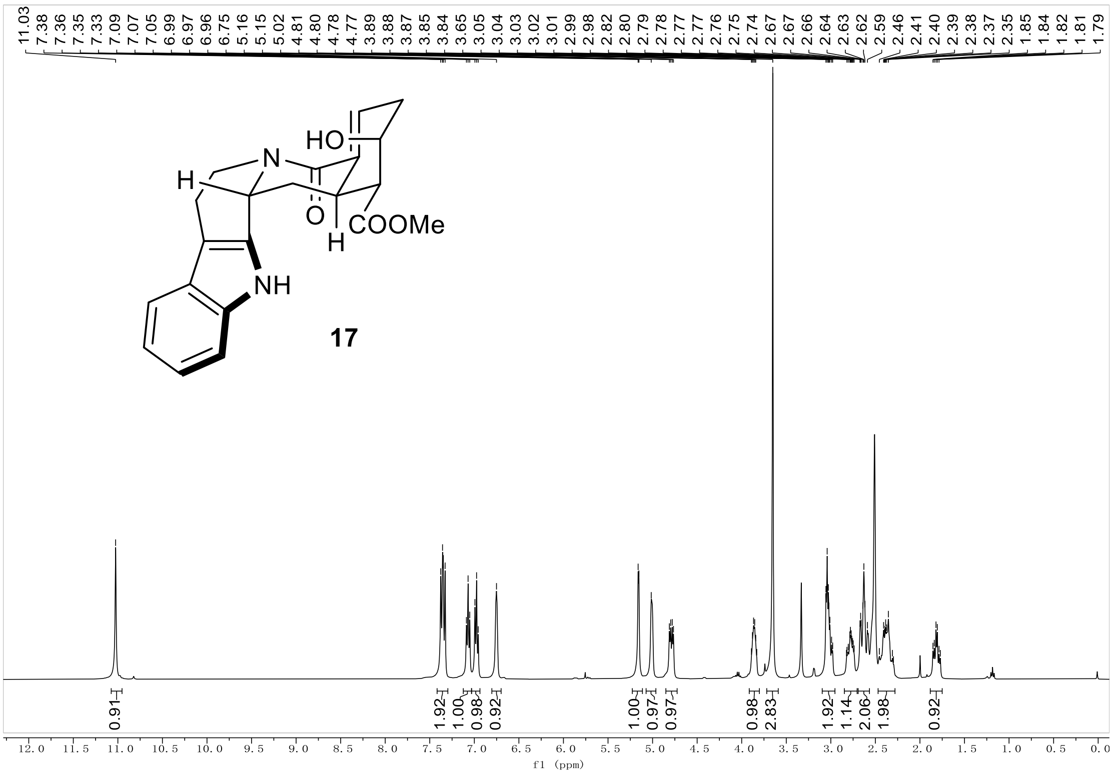

Supplementary Figure 37. ^{13}C NMR of 17 (100 MHz, DMSO-d6)

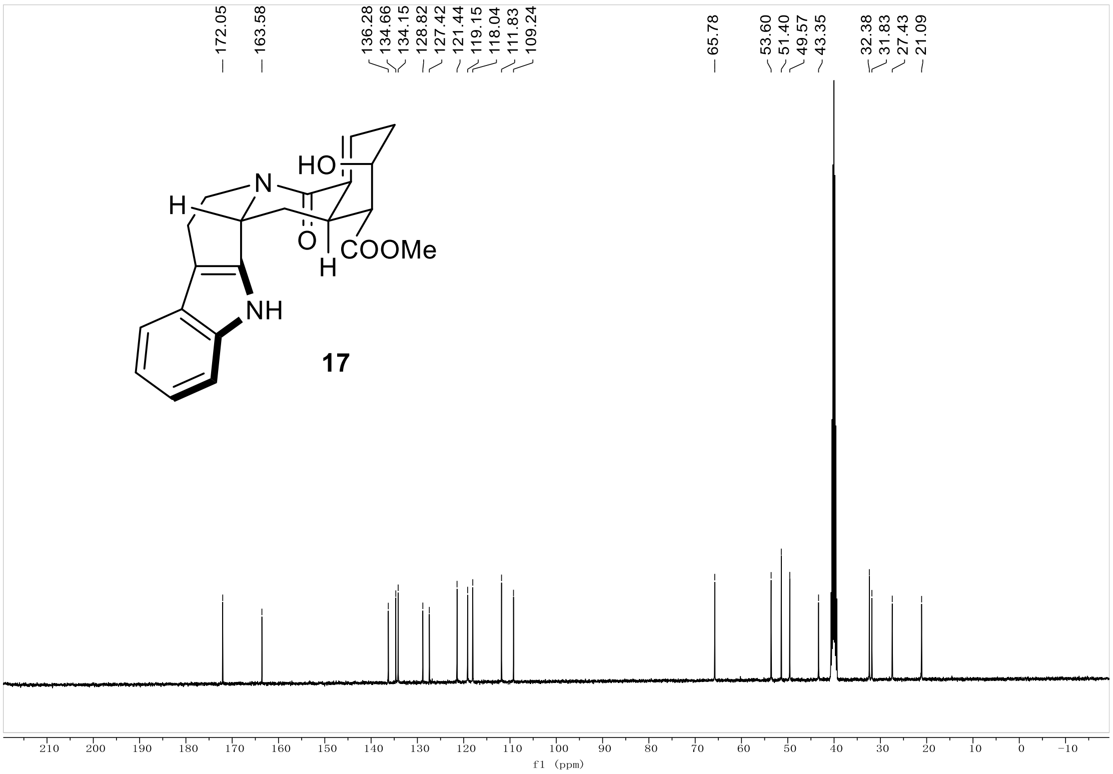

Supplementary Figure 38. ^{1}H NMR of 18 (400 MHz, DMSO-d6)

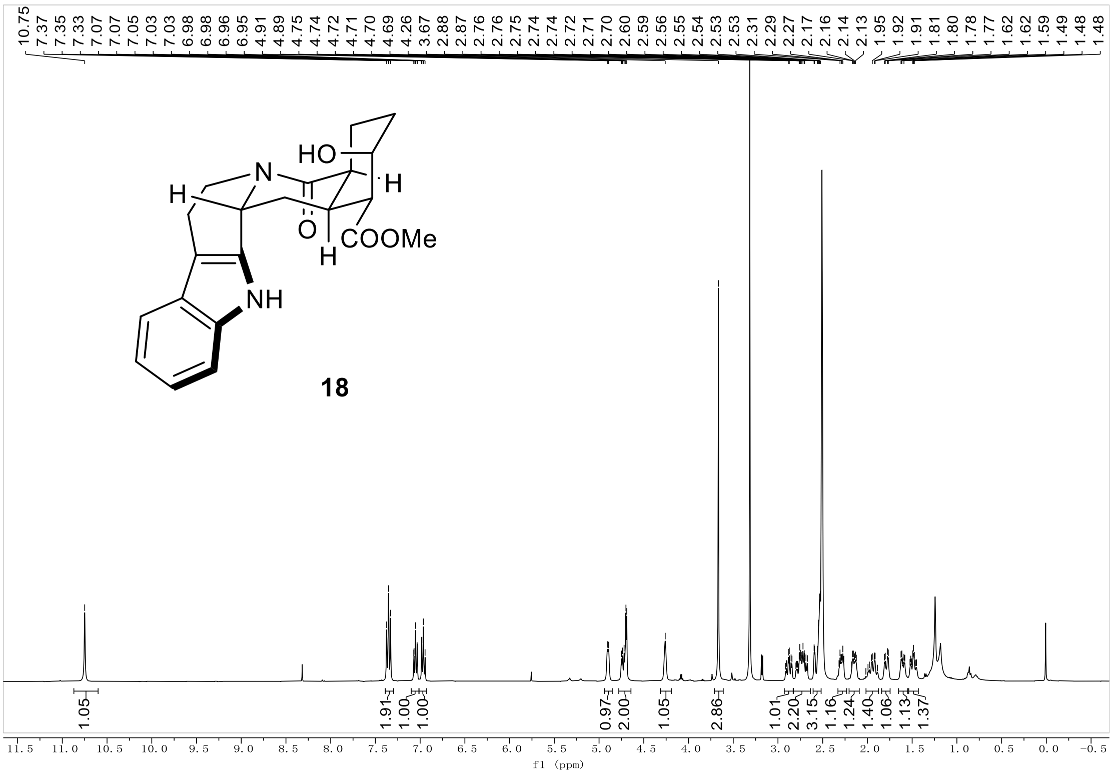

Supplementary Figure 39. ^{13}C NMR of 18 (100 MHz, DMSO-d6)

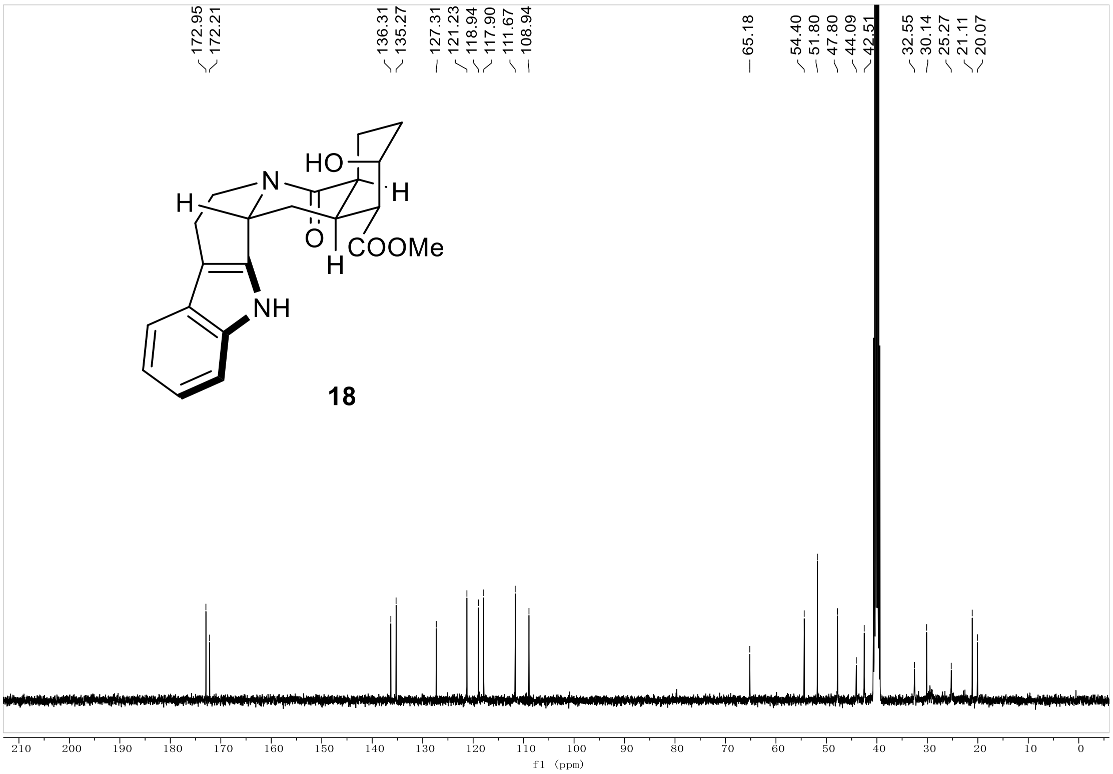

Supplementary Figure 40. ^{1}H NMR of 15 (400 MHz, DMSO-d6)

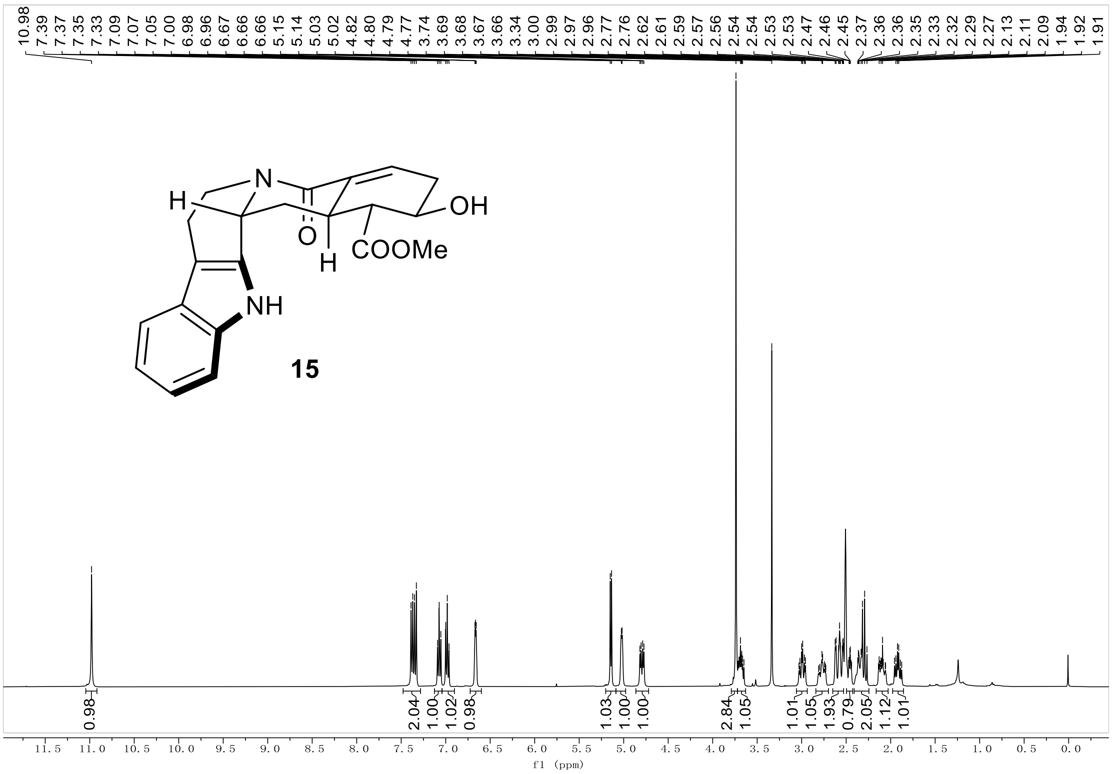

Supplementary Figure 41. ^{13}C NMR of 15 (100 MHz, DMSO-d6)

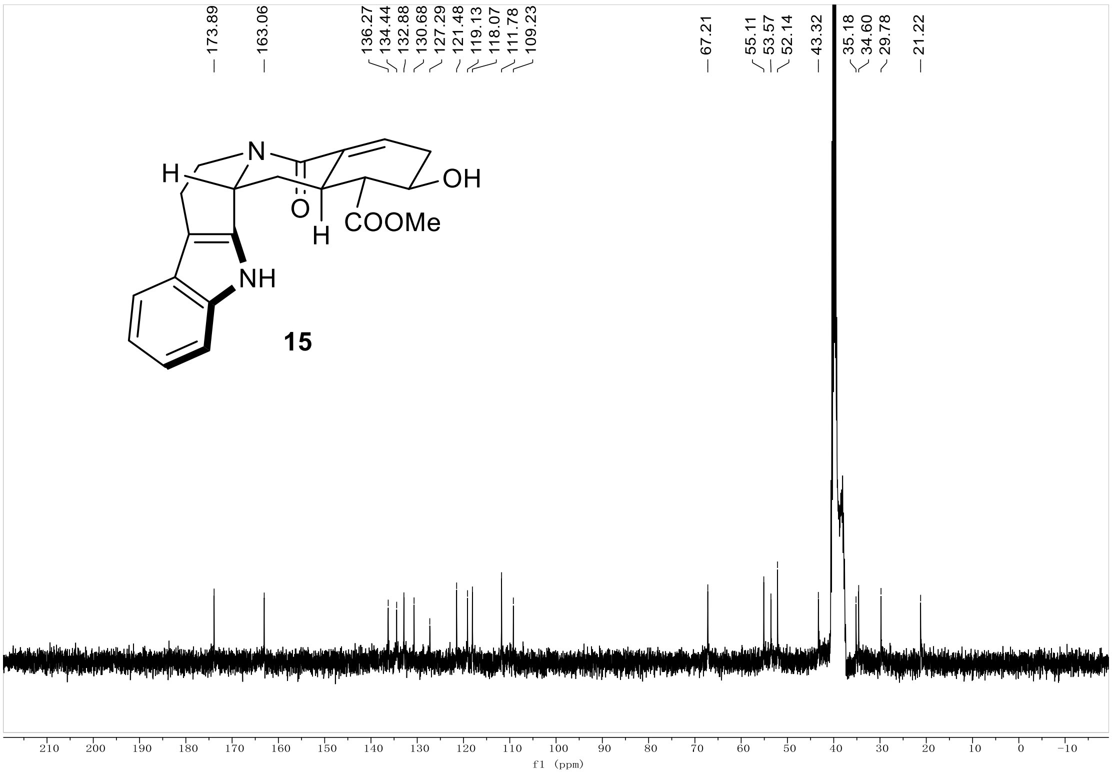

Supplementary Figure 42. ^{1}H NMR of 16 (400 MHz, DMSO-d6)

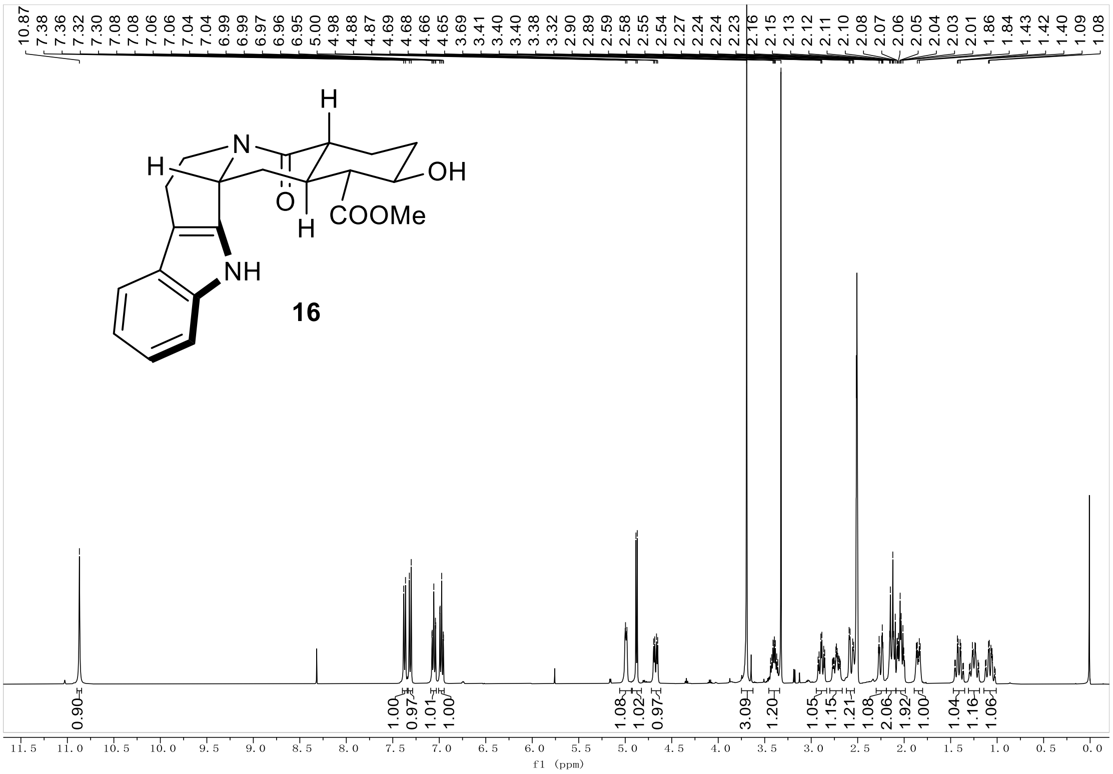

Supplementary Figure 43. ^{13}C NMR of 16 (100 MHz, DMSO-d6)

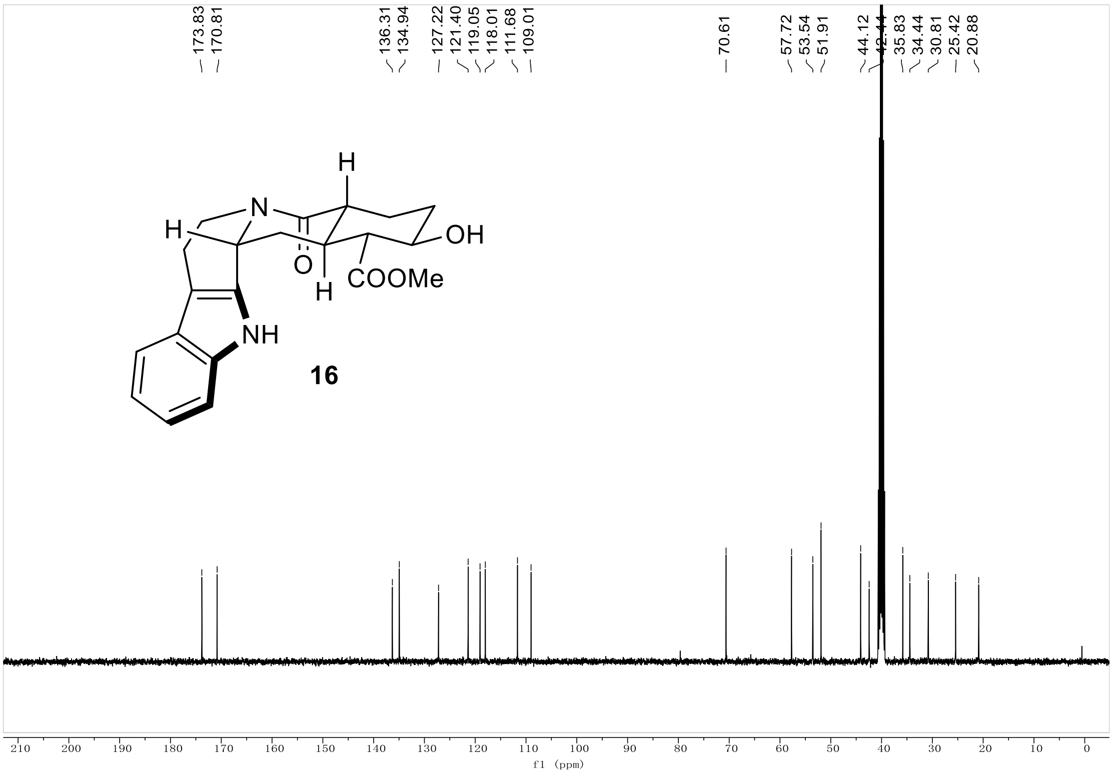

Supplementary Figure 44. ^{1}H NMR of 20 (400 MHz, DMSO-d6)

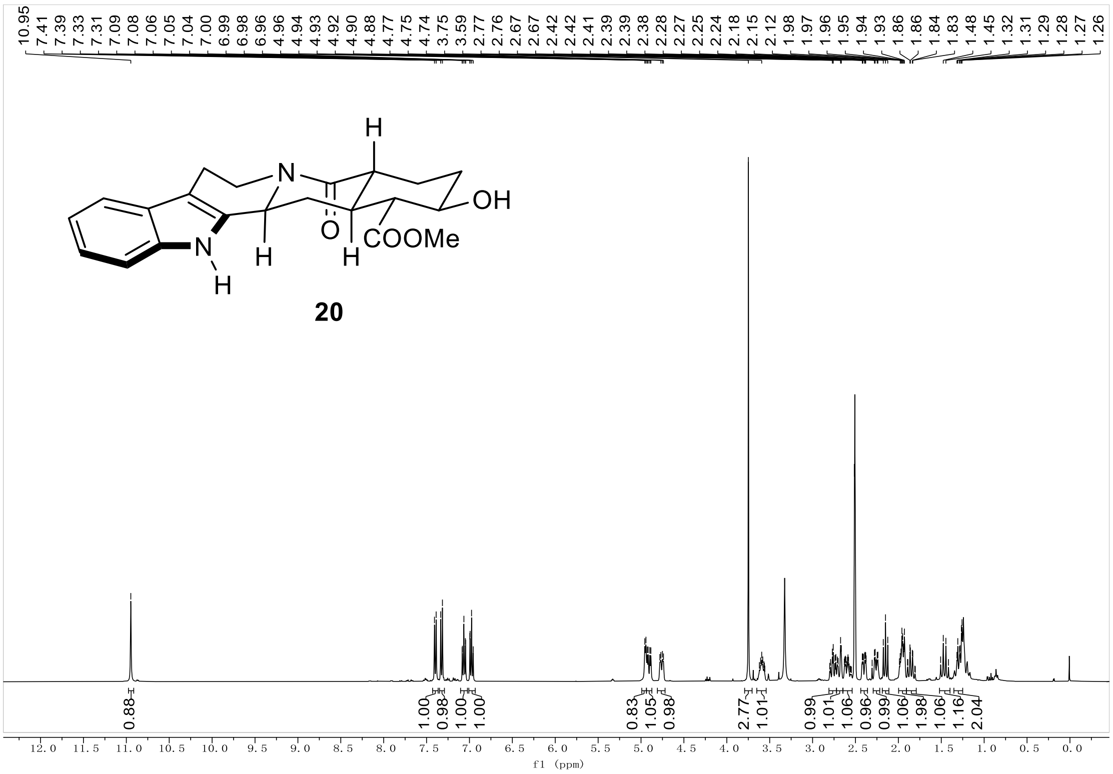

Supplementary Figure 45. ^{13}C NMR of 20 (100 MHz, DMSO-d6)

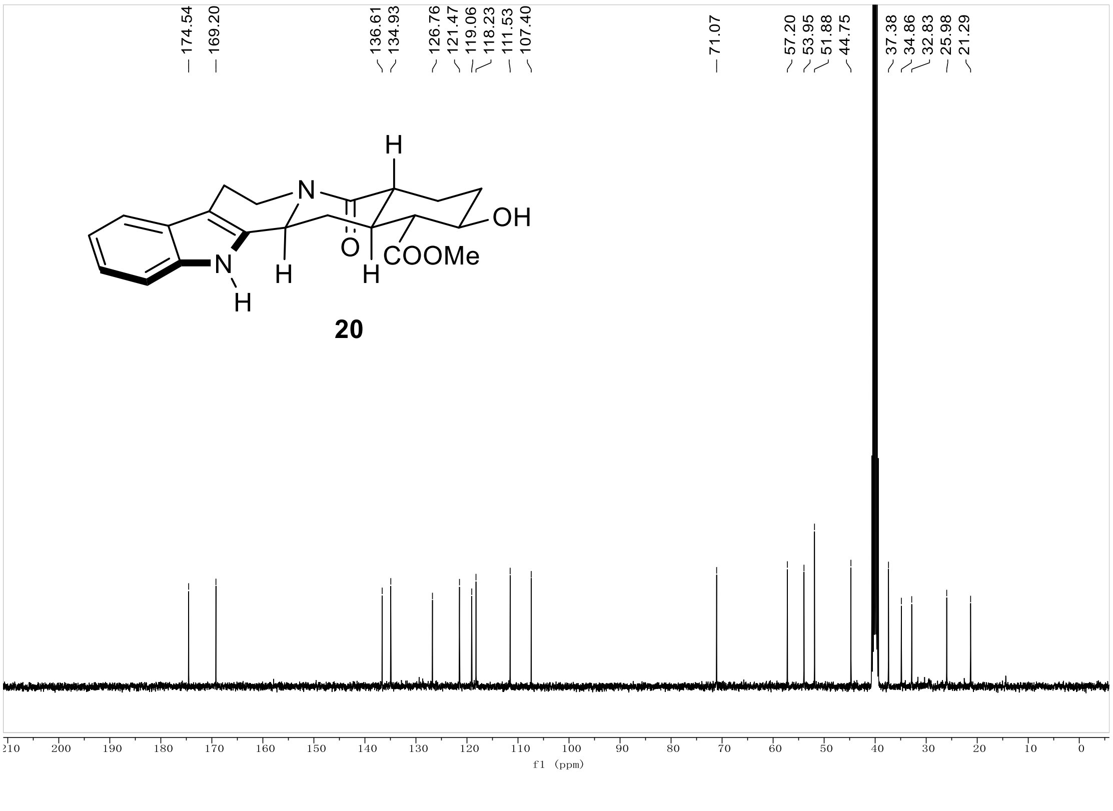

Supplementary Figure 46. ^{1}H NMR of 1b (400 MHz, $CDCl_3$)

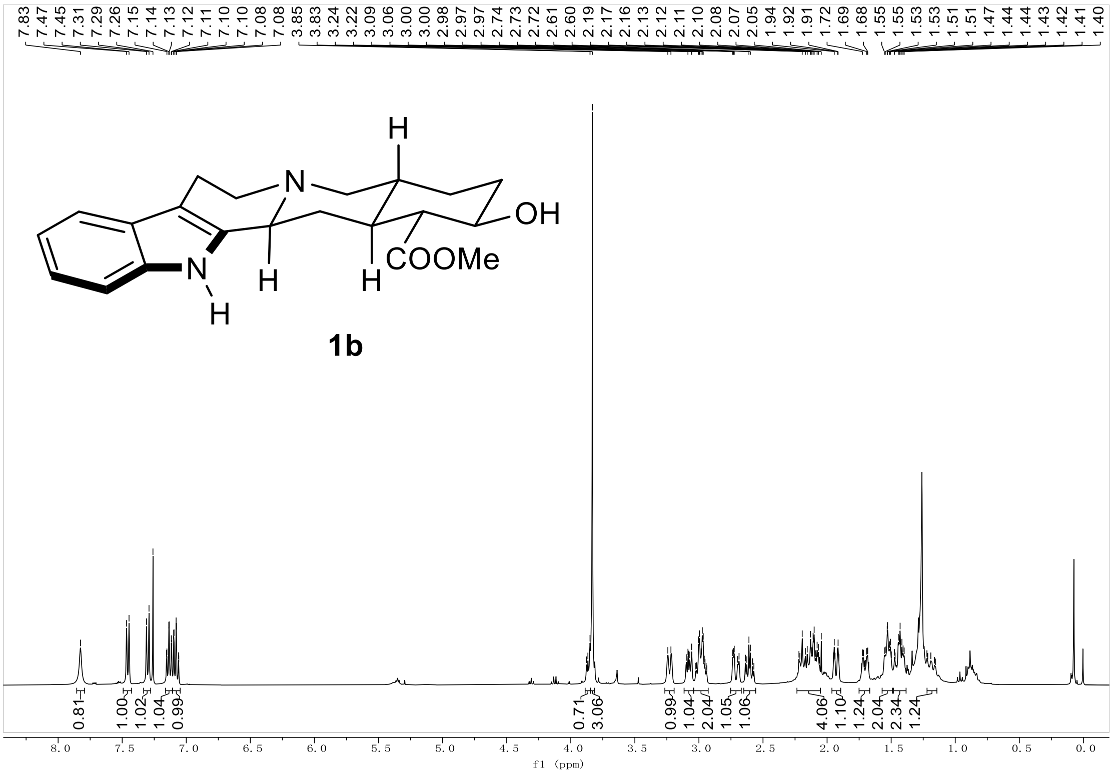

Supplementary Figure 47. ^{13}C NMR of 1b (100 MHz, $CDCl_3$)

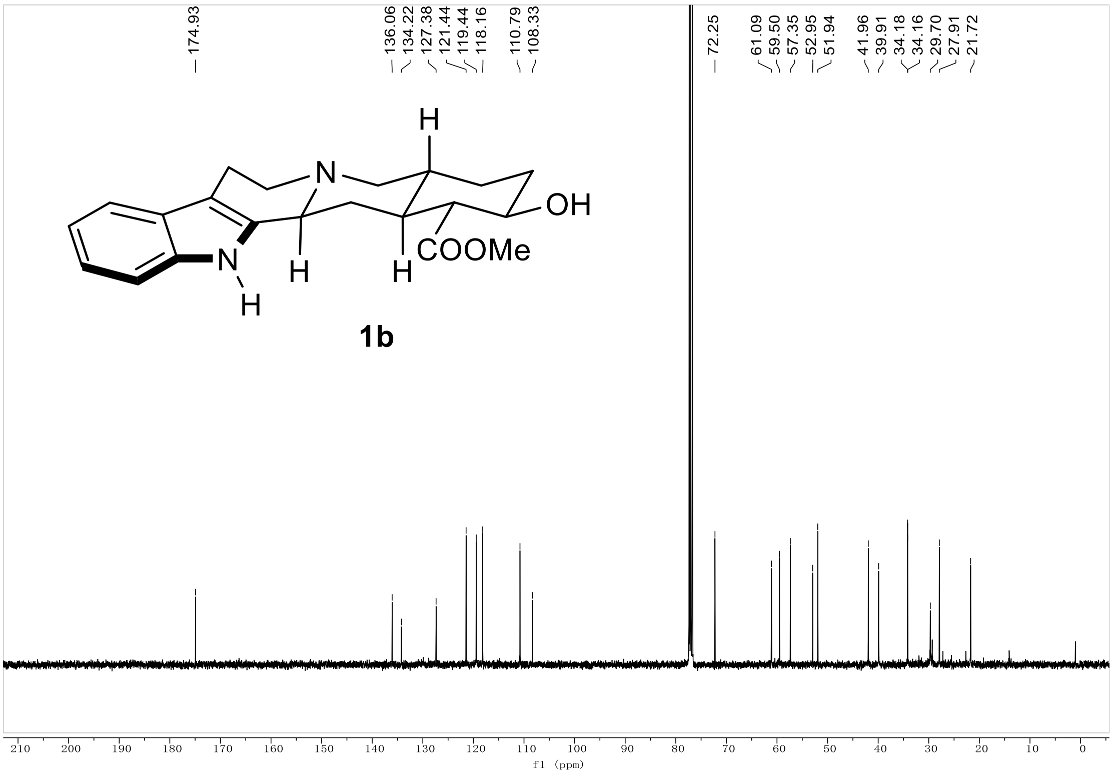

Supplementary Figure 48. ^{1}H NMR of 19 (400 MHz, $CDCl_3$)

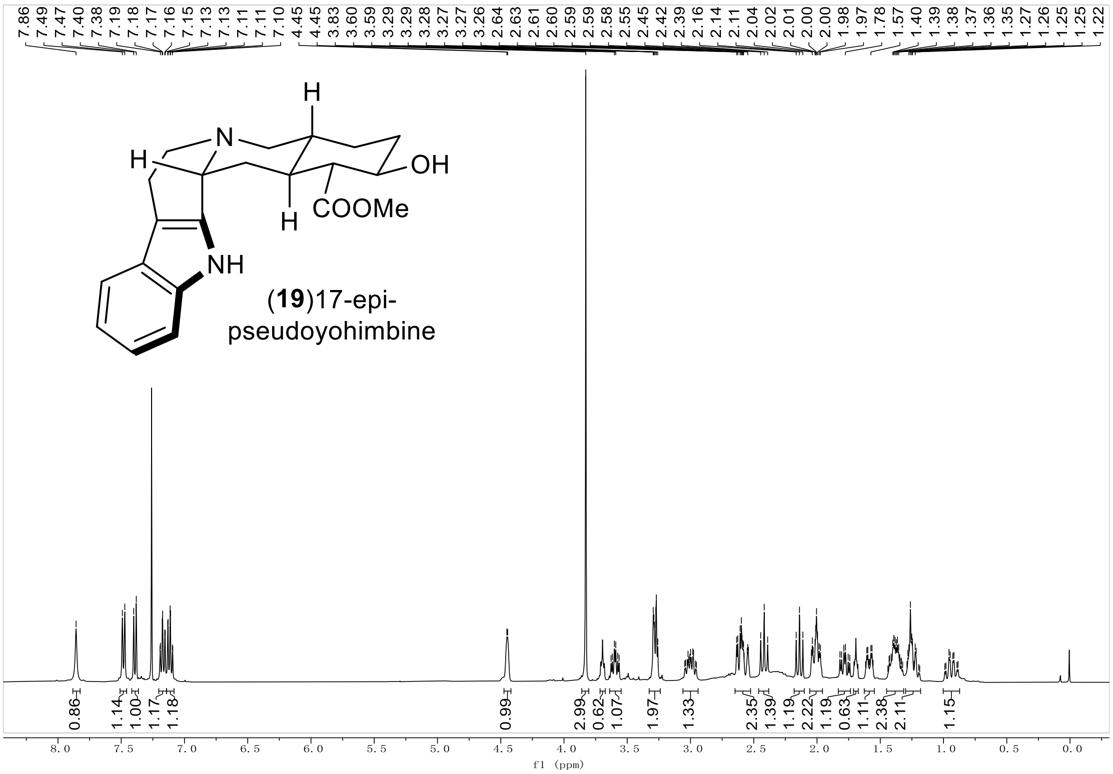

Supplementary Figure 49. ^{13}C NMR of 19 (100 MHz, $CDCl_3$)

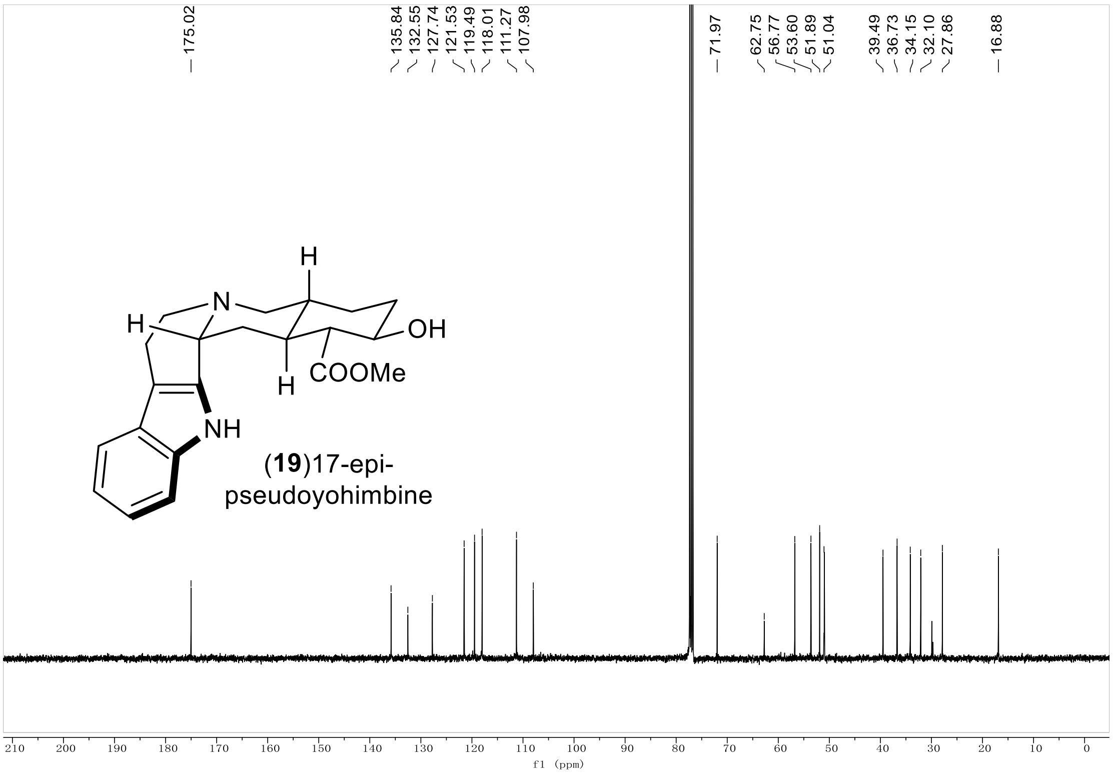

Supplementary Figure 50. ^{1}H NMR of 19’ (400 MHz, $CDCl_3$)

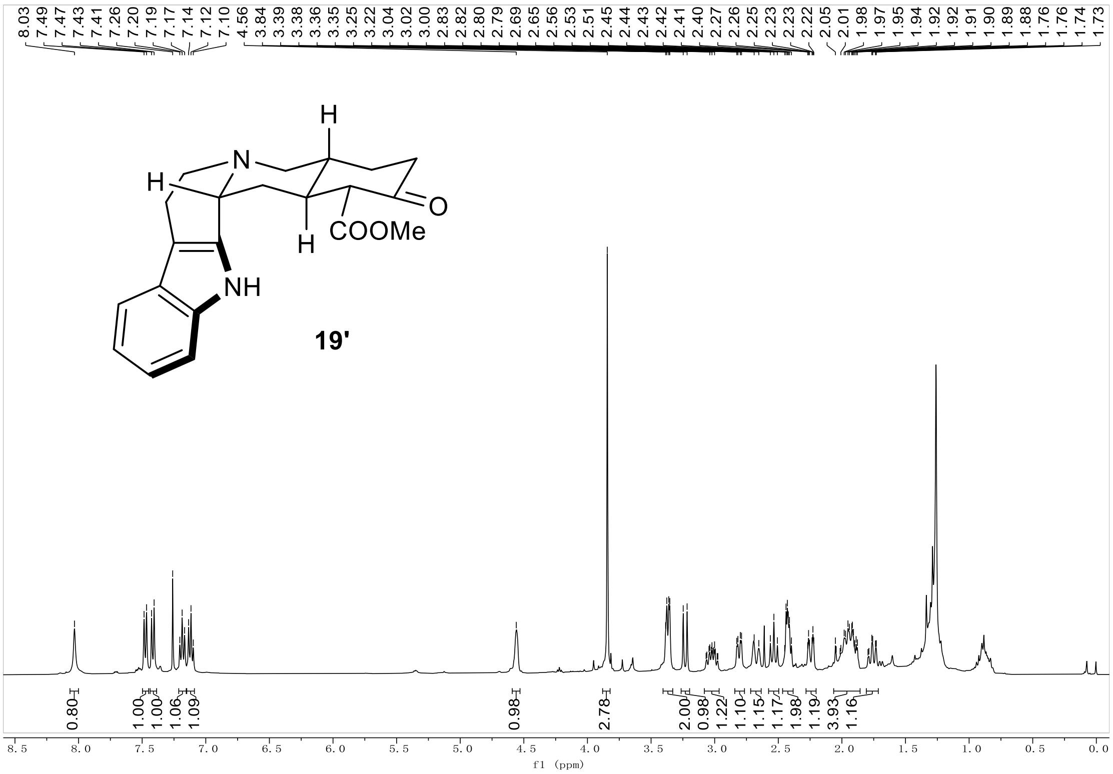

Supplementary Figure 51. ^{13}C NMR of 19’ (100 MHz, $CDCl_3$)

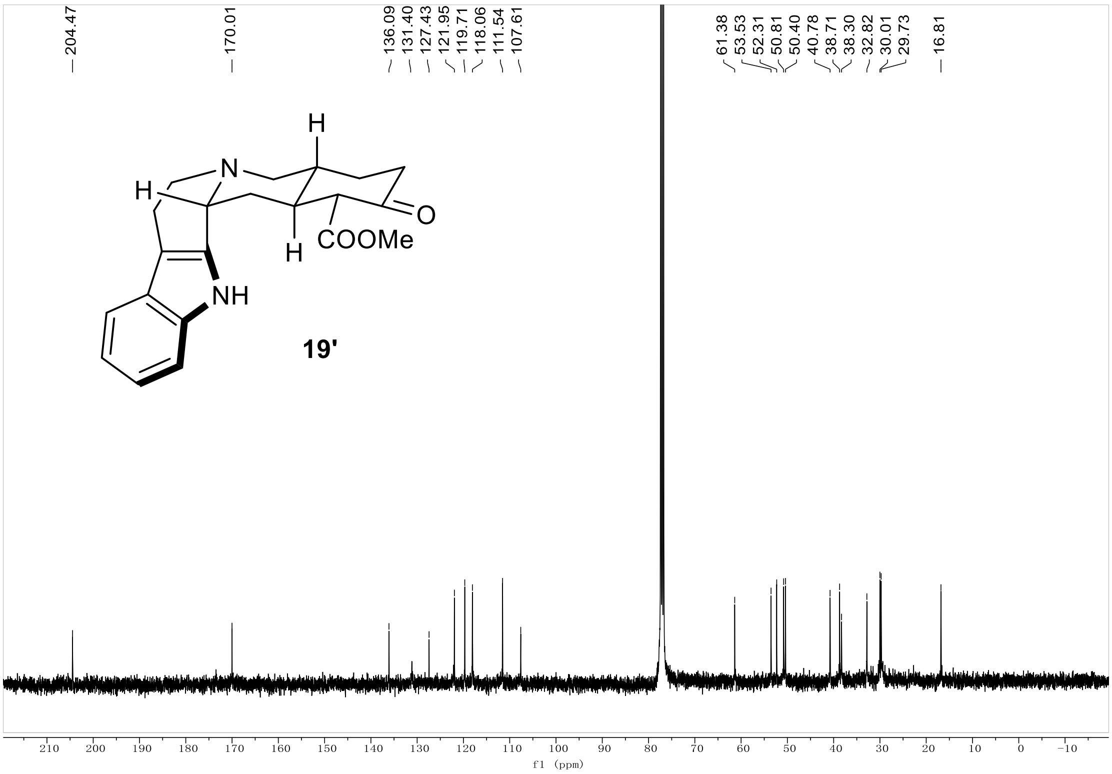

Supplementary Figure 52. ^{1}H NMR of 2 (400 MHz, $CDCl_3$)

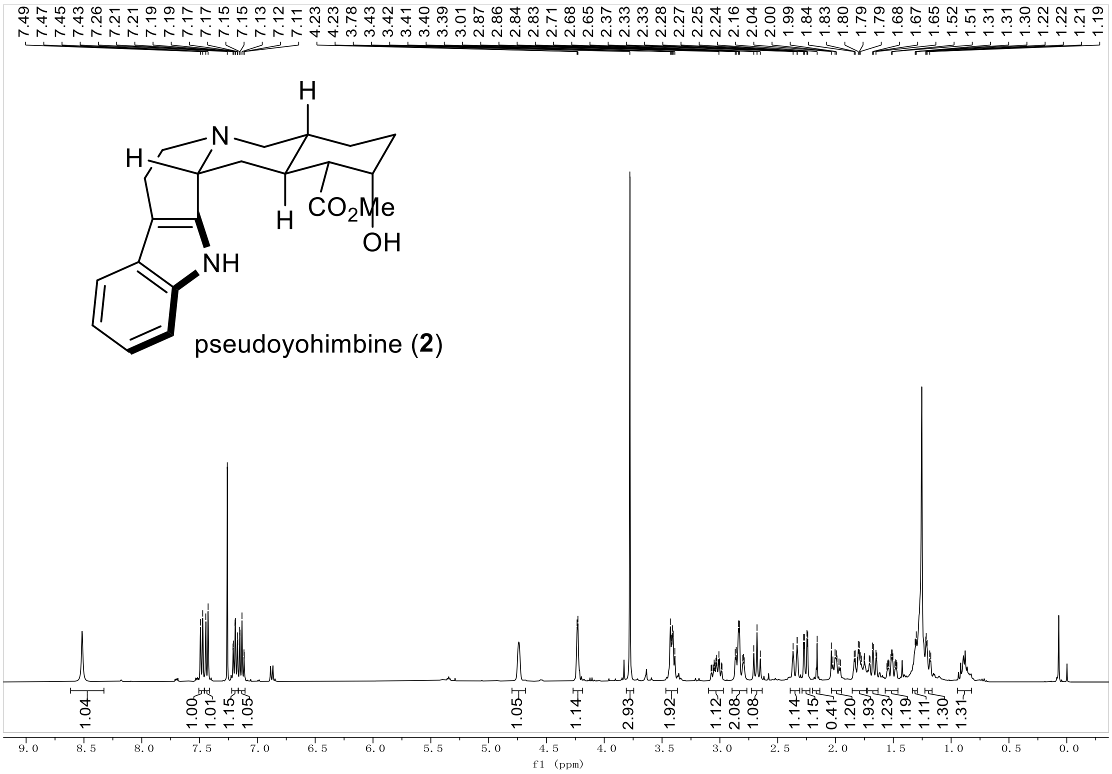

Supplementary Figure 53. ^{13}C NMR of 2 (100 MHz, $CDCl_3$)

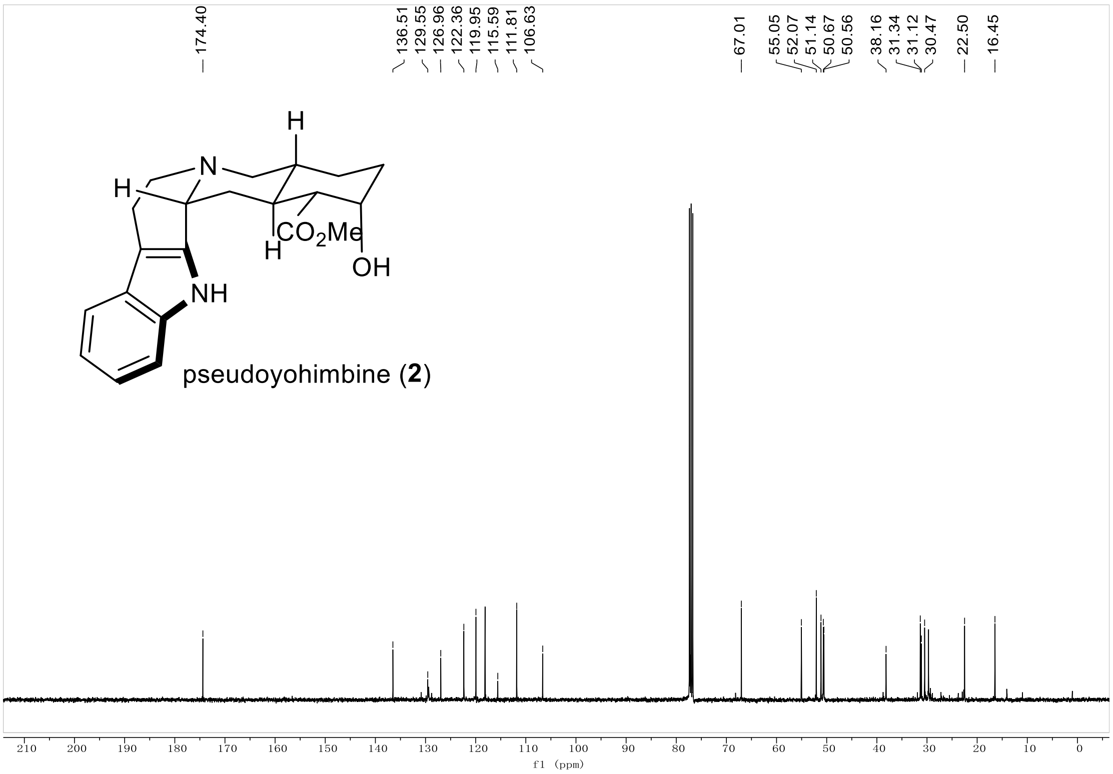

Supplementary Reference.

1. Williams, J. D., Otake, Y., Coussanes, G., Saridakis, I., Maulide, N., Kappe, C. O. Towards a scalable synthesis of 2-oxabicyclo[2.2.0]hex-5-en-3-one using flow photochemistry. *ChemPhotoChem* **3**, 229-232 (2019).
2. Lebold, T. P., Wood, J. L., Deitch, J., Lodewyk, M. W., Tantillo, D. J., Sarpong, R. A divergent approach to the synthesis of the yohimbine alkaloids venenatine and alstovenine. *Nat. Chem.* **5**, 126−131 (2013).
3. Wang, X., Xia, D., Qin, W., Zhou, R., Zhou, X., Zhou, Q., Liu, W., Dai, X., Wang, H., Wang, S., Tan, L., Zhang, D., Song, H., Liu, X.- Y., Qin, Y. A radical cascade enabling collective syntheses of natural products. *Chem* **2**, 803-816 (2017).
4. Brown, R. T., Pratt, S. B., Richards, P. Enantiospecific synthesis of (-)-3-iso-19,20-dehydro-β-yohimbine from secologanin: a route to normal and pseudo stereoisomers of yohimbine. *Tetrahedron Lett.* **41**, 5627-5630 (2000).
